# A Bioorthogonal Precision Tool for Human *N*-Acetylglucosaminyltransferase V

**DOI:** 10.1021/jacs.4c05955

**Published:** 2024-09-17

**Authors:** Yu Liu, Ganka Bineva-Todd, Richard W. Meek, Laura Mazo, Beatriz Piniello, Olga Moroz, Sean A. Burnap, Nadima Begum, André Ohara, Chloe Roustan, Sara Tomita, Svend Kjaer, Karen Polizzi, Weston B. Struwe, Carme Rovira, Gideon J. Davies, Benjamin Schumann

**Affiliations:** † Department of Chemistry, Imperial College London, London W12 0BZ, U.K.; ‡ Chemical Glycobiology Laboratory, The Francis Crick Institute, London NW1 1AT, U.K.; § York Structural Biology Laboratory, Department of Chemistry, University of York, Heslington, York YO10 5DD, U.K.; ∥ School of Biological Sciences, Faculty of Environmental and Life Sciences, University of Southampton, Southampton SO17 1BJ, U.K.; ⊥ Departament de Química Inorgànica i Orgànica (Secció de Química Orgànica) and Institut de Química Teòrica i Computacional (IQTCUB), Universitat de Barcelona, Martí i Franquès 1, 08028 Barcelona, Spain; # Structural Biology Science Technology Platform, The Francis Crick Institute, London NW1 1AT, U.K.; ∇ Department of Chemical Engineering and Imperial College Centre for Synthetic Biology, Imperial College London, London SW7 2AZ, U.K.; ○ Institució Catalana de Recerca i Estudis Avançats (ICREA), Passeig Lluís Companys 23, 08020 Barcelona, Spain; ◆ Department of Biochemistry, Dorothy Crowfoot Hodgkin Building, University of Oxford, South Parks Road, Oxford OX1 3QU, U.K.; ¶ The Kavli Institute for Nanoscience Discovery, Dorothy Crowfoot Hodgkin Building, University of Oxford, South Parks Road, Oxford OX1 3QU, U.K.

## Abstract

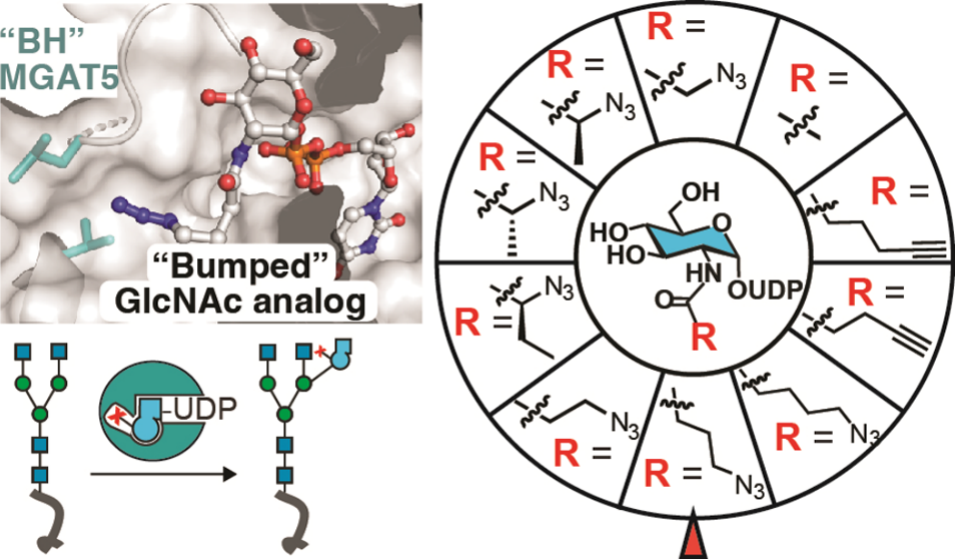

Correct elaboration of N-linked glycans in the secretory pathway of human cells is essential in physiology. Early N-glycan biosynthesis follows an assembly line principle before undergoing crucial elaboration points that feature the sequential incorporation of the sugar *N*-acetylglucosamine (GlcNAc). The activity of GlcNAc transferase V (MGAT5) primes the biosynthesis of an N-glycan antenna that is heavily upregulated in cancer. Still, the functional relevance and substrate choice of MGAT5 are ill-defined. Here, we employ protein engineering to develop a bioorthogonal substrate analog for the activity of MGAT5. Chemoenzymatic synthesis is used to produce a collection of nucleotide-sugar analogs with bulky, bioorthogonal acylamide side chains. We find that WT-MGAT5 displays considerable activity toward such substrate analogues. Protein engineering yields an MGAT5 variant that loses activity against the native nucleotide sugar and increases activity toward a 4-azidobutyramide-containing substrate analogue. By such restriction of substrate specificity, we show that the orthogonal enzyme–substrate pair is suitable to bioorthogonally tag glycoproteins. Through X-ray crystallography and molecular dynamics simulations, we establish the structural basis of MGAT5 engineering, informing the design rules for bioorthogonal precision chemical tools.

## Introduction

Glycans are essential modulators of human health. Asn (N)-linked glycans are found in abundance on proteins trafficking through the secretory pathway. Dysregulated biosynthesis of N-glycans is associated with congenital disorders of glycosylation that often manifest in neurological and developmental disabilities.^1^ N-glycans modulate folding, stability, and biochemical activity of proteins, with manifold interactions mediated by glycosylation that are relevant for signaling, trafficking, and immune function.^2−4^ Certain structural features of N-glycans, such as increased branching, have been related to tumor progression.^5^

**Figure 1:**
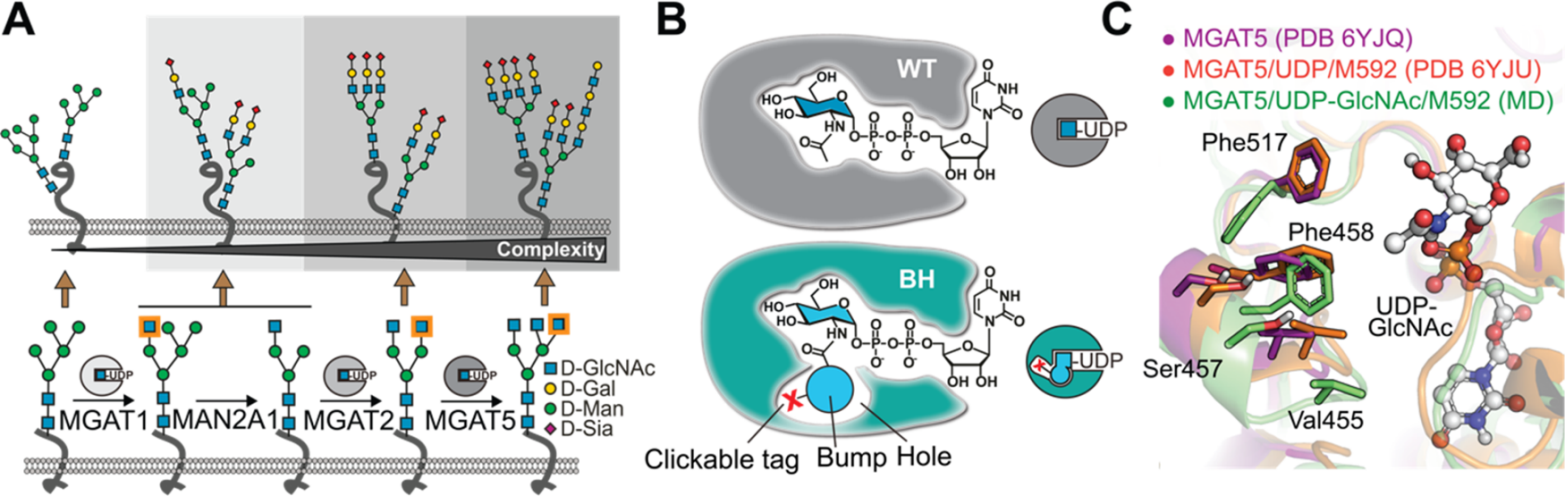
Principle of MGAT5 bump-and-hole engineering. (A) GlcNAc addition by MGAT1, MGAT2 and MGAT5 presents bifurcation points in the biosynthesis of N-glycans, with intermediate trimming by MAN2A1. New GlcNAc moieties added in each step are highlighted in orange. (B) Bump-and-hole (BH) engineering to generate a new MGAT5 enzyme–substrate pair that uses bumped, bioorthogonal UDP-GlcNAc analogs as substrates. (C) MGAT5 gatekeeper residues chosen by proximity to the UDP-GlcNAc acetamide in published crystal structures and our own molecular dynamics (MD) data.^20^

Unlike proteins and nucleic acids, glycans are refractory to simple manipulation with conventional methods of molecular biology. N-glycans are made in the secretory pathway in an enzymatic process reminiscent of an assembly line, with the glycosyltransferase (GT) repertoire in cells intricately influencing the structure of mature glycans.^3^ A common precursor rich in D-mannose (Man) and containing central Man-α-1-3-Man and Man-α-1-6-Man-linked arms is preassembled, transferred to polypeptides in the endoplasmic reticulum, and matured in the Golgi by the activity of glycosidases and GTs. Following initial trimming events, modification with the monosaccharide D-*N*-acetylglucosamine (GlcNAc) by the GlcNAc transferase MGAT1, trimming by Golgi α-mannosidase II MAN2A1 and subsequent GlcNAc introduction by MGAT2 and MGAT5, among others, determine the glycan subtype and the number of antennae found on the eventual mature glycan (Figure 1A).^6^ Among these modifications, introduction of GlcNAc to the Manα1-6Man arm to generate GlcNAc-α-1-6-Man is among the most frequent cancer-associated glycosylation events.^5,7^ Introduction of this 6-linked GlcNAc residue is mediated by the enzyme MGAT5, and ensuing elongation by other GTs can give rise to a long glycan chain that serves as a ligand to immunomodulatory galectins.^5,8−13^ Overexpression of *MGAT5* is frequently observed in a range of malignant tumors and generally associated with a worse prognosis.^5,14^

While the biosynthetic hierarchy of GlcNAc introduction into growing N-glycans has been recapitulated *in vitro*,^15−19^ it is not clear why different glycosylation sites carry structurally different N-glycans that can either display or lack the 6-GlcNAc antenna. Through a combination of crystallography and computational modeling, we have shown that MGAT5 undergoes substantial structural rearrangements upon binding the nucleotide-sugar donor uridine diphospho(UDP)-GlcNAc.^20^ Existing crystal structures highlight the binding mode of the natural heptasaccharide acceptor glycan termed NGA2, often by employing a truncated pentasaccharide termed M592 that lacks the terminal chitobiose core.^20,21^ These data have led to the hypothesis that steric access to glycoprotein substrate might determine MGAT5 engagement. However, investigating this hypothesis will require methods to systematically inform which N-glycans on a substrate protein are specifically modified by MGAT5.

Chemical tools have fundamentally transformed glycobiology. Considerable synthetic efforts have led to candidate inhibitors for MGAT5 based on analogs of UDP-GlcNAc,^22^ glycan acceptor,^23−29^ or hybrid structures,^21^ and have aided in understanding the complex biology of the enzyme. In turn, bioorthogonal sugars, including azides or alkynes usually as part of modified acylamides, serve as metabolic reporter tools for glycosylation.^30,31^ For instance, the sugar GlcNAz bearing a 2-azidoacetamide has been widely used in glycoproteome analysis.^30,32−34^ Cellular biosynthetic enzymes convert bioorthogonal sugars such as GlcNAz with small modifications to UDP-GlcNAc analogs that are then used by a range of GTs. The secretory pathway hosts a number of structurally diverse GlcNAc transferases that use such substrates. Some GlcNAc transferases, including MGAT2 and MGAT5, display significant promiscuity toward chemical modifications, including larger diazirine-containing acylamides.^34^ Therefore, no bioorthogonal reporter tools are available that are selective for individual GlcNAc transferases such as MGAT5.

Based on our structural data, we envisioned that a tactic termed bump-and-hole (BH) engineering could be applicable to generate a bioorthogonal reporter tool for MGAT5. In this tactic, an enzyme is engineered (to contain a “hole”) by replacing large hydrophobic residues with smaller amino acids, creating space for a ligand “bump” that is in this case the acylamide of a bioorthogonal UDP-GlcNAc analog (Figure 1B).^35−38^ In previous renditions of glycosyltransferase BH engineering, two structural aspects contributed to orthogonality of enzyme–substrate pairs. First, the WT enzyme often has little activity toward bumped substrate analogues. Second, due to the enlargement of the substrate binding site and the absence of hydrophobic interactions, the BH-enzyme loses affinity toward the native nucleotide-sugar.^36−38^ While both aspects are generally desirable, the precise features that contribute to a successful BH approach have not been mapped in detail. Furthermore, no comprehensive BH campaign has been undertaken for GlcNAc transferases in the secretory pathway as of yet.^39^

Here, we report the development of a BH strategy for MGAT5 as a GlcNAc transferase with outstanding biological relevance. Our approach features the structure-informed design of an MGAT5 BH enzyme–substrate pair, fueled by the chemoenzymatic synthesis of a collection of bumped, bioorthogonal UDP-GlcNAc analogs. We find that WT-MGAT5 displays promiscuity toward such analogues. Engineering the active site introduces substrate specificity mainly by restricting activity toward the native substrate UDP-GlcNAc, in addition to increasing acceptance of a 4-azidobutyramide-containing analog.

## Results and Discussion

### Structurally Informed Bump-and-Hole Mutagenesis of MGAT5

An essential prerequisite to BH engineering is the identification of hydrophobic residues termed “gatekeepers” in proximity to the substrate functionality to be chemically modified (i.e., the acetamide).^36^ Ideally, this process builds on a model of the complex between nucleotide-sugar and GT which can be challenging to obtain by crystallography. Our previous work enhanced a ternary MGAT5/UDP/M592 cocrystal structure, introducing UDP-GlcNAc by molecular dynamics (MD) and quantum mechanics/molecular mechanics (QM/MM).^20^ With this orientation of UDP-GlcNAc in the active site in hand, we commenced with identifying potential gatekeeper residues (Figure 1C). The residues Val455 and Phe458 are in immediate proximity to the GlcNAc acetamide, and are part of a short α-helix that recruits UDP-GlcNAc via a strong interaction of Lys454 with one of the UDP phosphates.^20^ Phe517 is also part of the hydrophobic pocket that accommodates the GlcNAc acetamide in the modeled MGAT5/UDP-GlcNAc/M592 structure (Figure 1C). Based on the synergy of crystallography and MD simulations, we chose Val455, Phe458, and Phe517 as suitable residues for protein engineering. We generated constructs of MGAT5 in which Phe458 was substituted with Ala, Gly or Val. These single mutants were further analyzed in combination with secondary mutants in Phe517 (Ala or Leu), Val455 (Ala) and Ser457 (Gly or Ala), rationalized by the additional space such changes would provide for chemical modifications. In total, 12 MGAT5 variants with rationally chosen amino acid substitutions were produced as soluble, His_6_-tagged constructs from insect cells for *in vitro* validation (Supporting Figure 1).

### Chemoenzymatic Synthesis of Bumped, Bioorthogonal UDP-GlcNAc Analogs

**Figure 2:**
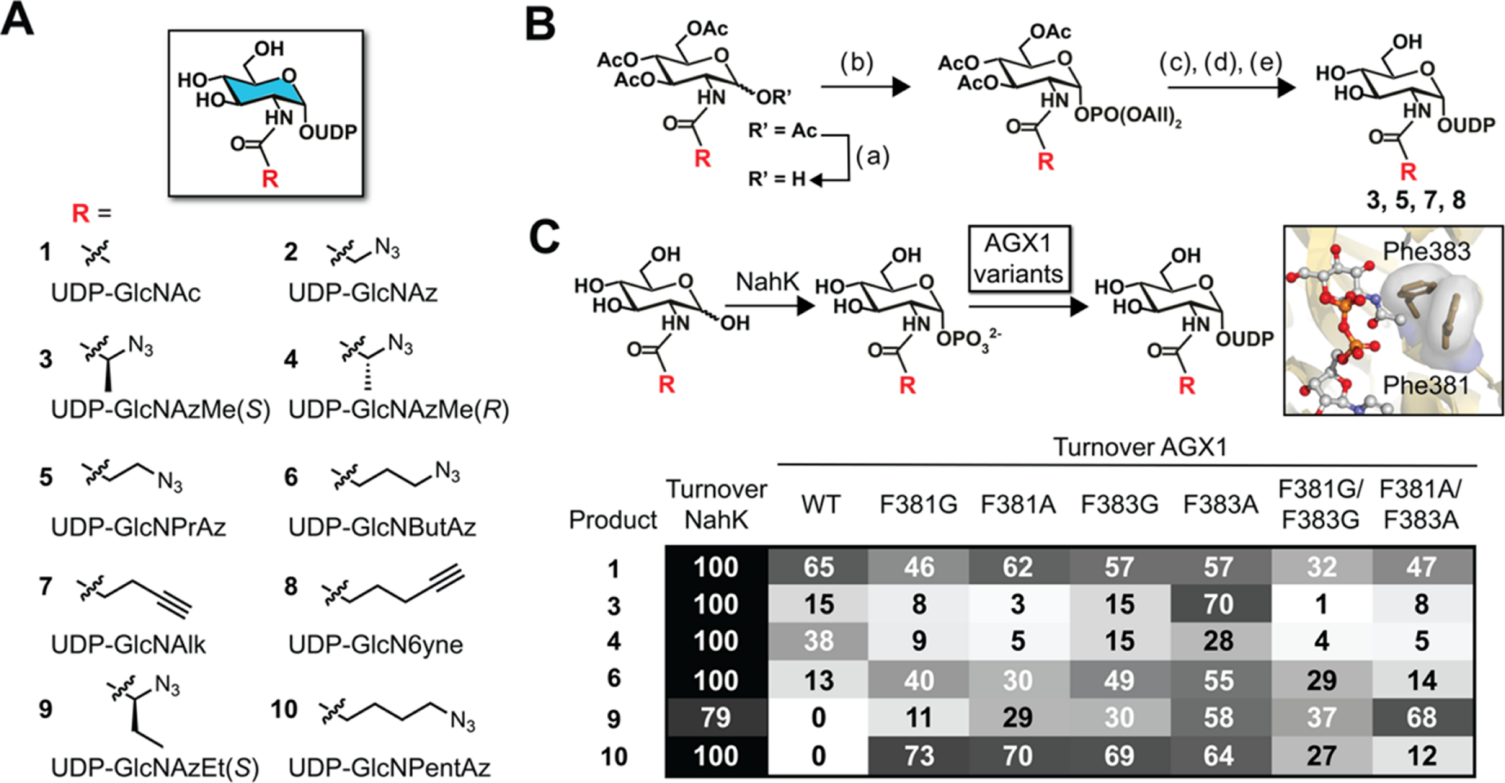
Synthesis of bumped, bioorthogonal UDP-GlcNAc analogs. (A) Overview of analogs. (B) Chemical synthesis to generate analogs **3**, **5**, **7**, and **8**. (C) Chemoenzymatic synthesis of UDP-GlcNAc **1** and analogs **3**, **4**, **6**, **9**, and **10** employing NahK and engineered variants of human AGX1. Insert: crystal structure of human AGX1 with UDP-GlcNAc (PDB 1JV1). Turnover was monitored by UPLC with UV absorption of UDP-sugars normalized to a standard curve, or by LC-MS with peak integration normalized to sugar-1-phosphate concentration for compound **9** since the NahK reaction did not proceed to completion. Data are means in single-enzyme assays (NahK) or coupled assays with NahK (AGX1 variants) from at least two independent replicates each. Reagents and conditions: (a) 3-(*N*,*N*-dimethylamino)-1-propylamine, THF, r.t., 2 h, 58–66%; (b) *i*Pr_2_NP(OAll)_2_, 1*H*-tetrazole, DCM, r.t, 30 min then *m*CPBA, – 40 °C, 30 min, 68–75%; (c) Pd(PH_3_)_4_, *p*TsSO_2_Na, THF, MeOH, r.t., 16 h; (d) Uridine monophosphomorpholidate, 1-methylimidazolium chloride, DMF, r.t., 16–32 h, 8.5% (over (c) and (d)); (e) Et_3_N, MeOH, H_2_O, r.t, 3–16 h, 58 or 6% over (c), (d), and (e).

Combinatorial evaluation of suitable BH enzyme–substrate pairs requires a collection of bumped, bioorthogonal substrate analogs. We targeted nine derivatives of UDP-GlcNAc **1** containing functionally diverse acylamide side chains based on our experience in engineering other glycosyltransferases.^36−38,40^ Analogs contained azides (**2**–**6**, **9**–**10**) or alkynes (**7**, **8**) as linear acylamides or with small (methyl, ethyl) branches (Figure 2A).^37,41−43^ Analogs **3**, **5**, **7** and **8** were synthesized employing phosphoramidite chemistry (Figure 2B).^40,42^ To streamline syntheses of other UDP-GlcNAc analogs, a chemoenzymatic workflow was then established using transferases from human or bacterial sources.^41,43−48^ A limitation of human biosynthetic enzymes is their low tolerance toward acetamide modifications larger than a simple azide.^37,41,45,49^ We have recently established a method to generate analogs of *N*-acetylhexosamines with sterically more demanding acylamides in cells,^41,42,45^ which was employed by Cappicciotti and colleagues in a one-pot multienzyme (OPME) procedure *in vitro*.^43,45^ The approach features the bacterial kinase NahK from *Bifidobacterium longum* to phosphorylate the anomeric hydroxyl group. Engineered variants of the human pyrophosphorylases AGX1 or AGX2 then convert sugar-1-phosphates to UDP-sugars.^41,43,50,51^ Supported by structural data, AGX1 variants commonly replace Phe383 with smaller amino acids to enlarge the active site and, in turn, accommodate bulkier sugar-1-phosphate analogs.^51,52^ While this OPME approach has been used to synthesize a small collection of chemically tagged UDP-sugar analogs,^41,43^ the substrate scope is currently not known. We first established that recombinant NahK catalyzes the initial phosphorylation step for all GlcNAc analogs in 79 to 100% conversion as monitored via ultraperformance liquid chromatography (UPLC) with mass spectrometry detection (Figure 2C).

We then used recombinant AGX1 variants for UDP-sugar synthesis.^45^ Alternatively to substituting Phe383 to Ala and Gly, we substituted the Phe381 residue that is also in proximity to the acetamide side chain, generating a total of six engineered AGX1 constructs with single and double substitutions.^42,52^ GlcNAc analogs were converted to UDP-sugars **1**, **3**, **4**, **6**, **9**, and **10** in 15 to 70% conversion by the combination of NahK with AGX1^F383A^ or AGX1^F383G^ (Figure 2C). The F383A variant displayed a slightly higher turnover for branched azides **4** and **9** than the F383G variant. Other AGX1 variants generally led to lower conversion, with notable exceptions such as the variant F381A/F383A which generated branched azide **9** in 68% conversion and the F381G variant that made linear azide **10** in 73% conversion. We concluded that NahK/AGX1^F383A^ is an efficient OPME system for the generation of a collection of bumped UDP-GlcNAc analogs. We employed suitable OPME conditions for the chemoenzymatic synthesis of UDP-GlcNAc analogs on preparative scale to fuel MGAT5 enzyme assays (Supporting Information).

Upon compound characterization by nuclear magnetic resonance spectroscopy (NMR), we noted that peaks corresponding to the vinylic protons in the uracil moiety (approximately δ = 7.85 and 5.95) appeared to distribute into several peaks that were not removed upon repeated purification efforts. We attributed these to either rotamers or tautomers.^53,54^ A variable temperature NMR experiment revealed that the lower-field peaks gradually disappeared when the temperature was increased from 30 to 70 °C, and reappeared upon cooling to room temperature (Supporting Information). These data confirmed rotamers or tautomers as the most likely sources for additional vinylic ^1^H NMR resonances.

### Development of an MGAT5 Bump-and-Hole Enzyme–Substrate Pair

**Figure 3:**
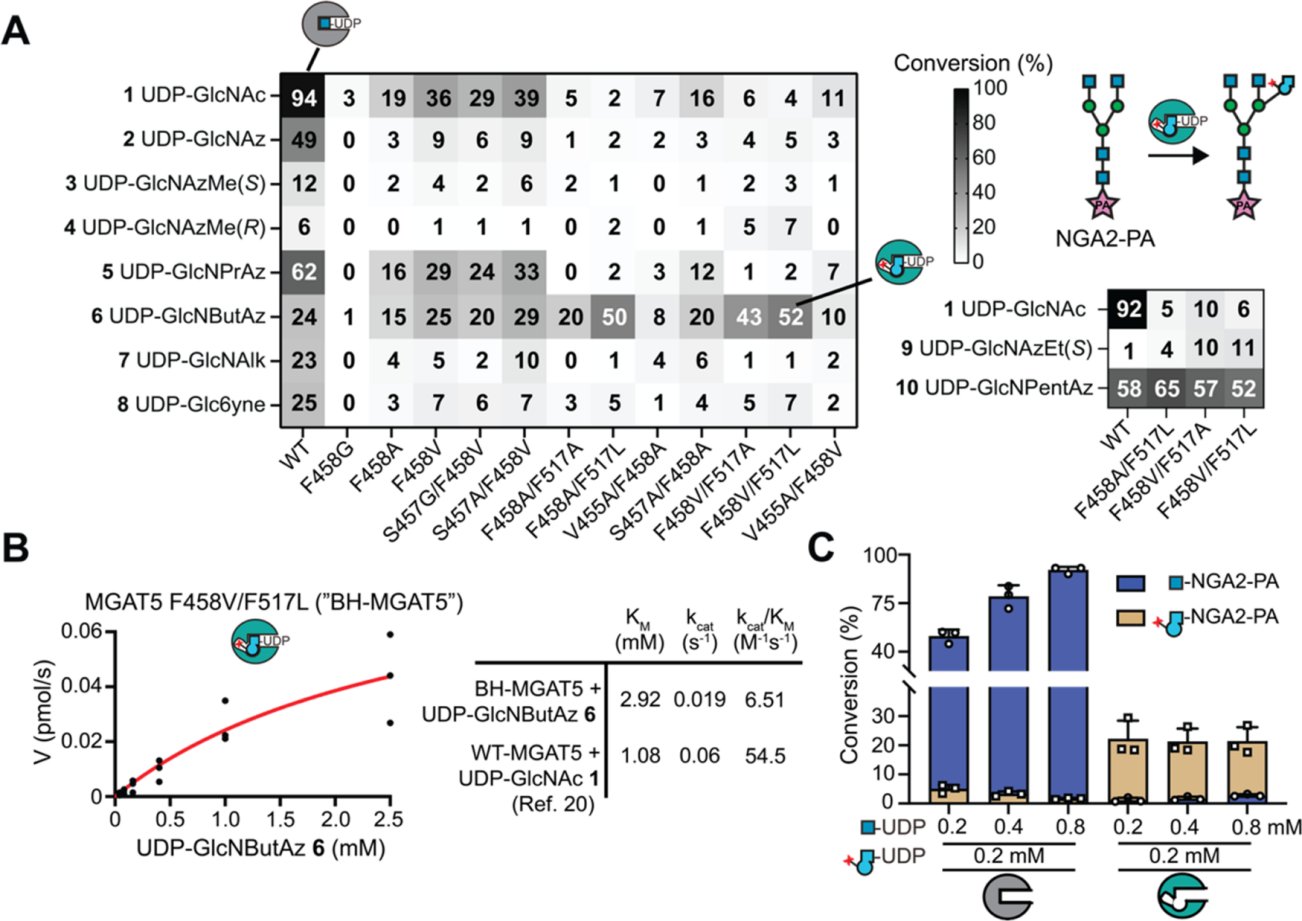
MGAT5 BH engineering. (A) MGAT5 variants were subjected to UDP-GlcNAc analogs in end point enzymatic assays, using procainamide (PA)-tagged NGA2 heptasaccharide as an acceptor substrate and analysis by UPLC with either UV absorption (**1** and analogs **2**, **6**, **8**, **10**) or MS detection (analogs **3**, **4**, **5**, **7**, **9**). Data are means from two independent replicates (see also Supporting Figure 3). (B) Michaelis–Menten enzyme kinetic experiment of BH-MGAT5 with UDP-GlcNButAz **6**. Constants were calculated based on three independent replicates per substrate concentration. (C) competition experiments employing both UDP-GlcNAc and UDP-GlcNButAz **6** in end point enzymatic experiments with WT- and BH-MGAT5. The reactions led to two octasaccharides that were individually quantified by UPLC with UV detection. Reaction mixtures contained 0.2 mM UDP-GlcNButAz **6** and either 0.2, 0.4, or 0.8 mM UDP-GlcNAc. Data are individual data points with means + SD from three independent replicates.

MGAT5 transfers GlcNAc to the NGA2 N-glycan intermediate in the secretory pathway. To assess whether engineered MGAT5 variants accepted synthetic UDP-sugar substrates in this reaction, we used procainamide (PA)-labeled NGA2 as an acceptor substrate in an *in vitro* enzymatic experiment with UPLC read-out.^15,19,22^ Employing a hydrophilic stationary phase, the resulting octasaccharides were detected by UV absorbance or MS integration, allowing turnover calculations (Supporting Figure 2). Two individual replicates were performed (Supporting Figure 3). WT-MGAT5 displayed a remarkable substrate promiscuity: In addition to using the natural substrate UDP-GlcNAc **1** (quantitative conversion) as well as the sterically nondemanding analog UDP-GlcNAz **2** (49%) as substrates, (Figure 3A), WT-MGAT5 accepted all other compounds **3**–**8** to varying (6–62%) degrees, especially UDP-GlcNAc analogs **5**–**8** with linear azide or alkyne side chains. In contrast, all engineered MGAT5 variants showed substantially lower conversion (2–40%) of UDP-GlcNAc **1** (max. 39%) and UDP-GlcNAz **2** (max. 9%), which we attribute to a lack of hydrophobic interactions between the acylamide side chains and the enlarged active sites. Gratifyingly, we observed a clear selectivity for acceptance of UDP-GlcNAc analog **6** termed UDP-GlcNButAz by MGAT5 variants with replacements of both Phe458 and Phe517 (Figure 3A). These variants typically showed low conversion (<10%) of all other compounds, especially the native substrate UDP-GlcNAc **1** (2–6%). The azide- and alkyne-tagged UDP-GlcNAc analogs **3**–**5** and **7**–**8** showed little acceptance by MGAT5 variants. Azide-tagged UDP-GlcNAc analogs **9**–**10** were only tried with the most successful MGAT5 variants with Phe458/Phe517 replacements, and displayed either low selectivity (**9**) or low conversion (**10**). Remarkably, WT-MGAT5 even accepted a 5-azidopentynamide modification in compound **10** as a substrate, confirming the substrate promiscuity of nonengineered enzyme. These data highlight an intriguing structure–activity relationship where substrate selectivity of BH-engineered MGAT5 is mainly driven by the loss of recognition of native substrate UDP-GlcNAc **1**. Furthermore, acceptance of the 4-azidobutyramide in **6** is somewhat enhanced from WT-MGAT5.

We further investigated the combination of UDP-GlcNButAz **6** with MGAT5^F458V/F517L^, now called BH-MGAT5, that showed the most favorable conversion among all putative BH-enzyme/substrate pairs. The Michaelis–Menten kinetic parameters *K*_M_ and *k*_cat_ of the BH-MGAT5/UDP-GlcNButAz **6** pair were 2- to 3-fold lower than for the WT-MGAT5/UDP-GlcNAc pair (Figure 3B). We concluded that BH-MGAT5 incorporates UDP-GlcNButAz **6** into acceptor glycans with turnover kinetics that are within an order of magnitude (8-fold reduction) of the WT-MGAT5 enzyme/substrate pair. Similar attributes have been found for successful BH approaches of other transferase families as well as other glycosyltransferases.^40,55,56^

Mindful that WT-MGAT5 accepted the bumped substrate UDP-GlcNButAz **6** to a certain degree in end point assays, we attempted to perform Michaelis–Menten analysis of this reaction but observed <4.5% conversion under the reaction conditions used for BH-MGAT5 even at the highest (2.5 mM) substrate concentration (Supporting Figure 4). A competition experiment was therefore designed in which WT- or BH-MGAT5 were subjected to different ratios of UDP-GlcNAc and UDP-GlcNButAz **6**, to mimic substrate exposure in the secretory pathway. After incorporation into acceptor substrate, both GlcNAc- and GlcNButAz-containing octasaccharides were detected. BH-MGAT5 strongly preferred UDP-GlcNButAz **6**, with an 8-fold higher incorporation of GlcNButAz into the acceptor even when UDP-GlcNAc was at a 4-fold excess. Strikingly, the inverse trend was observed for WT-MGAT5, accepting UDP-GlcNAc with 10-fold higher preference out of an equimolar mixture with UDP-GlcNButAz **6**. This ratio increased to 56-fold when UDP-GlcNAc was at a 4-fold excess, with barely measurable incorporation of GlcNButAz (Figure 3C). Taken together, BH-MGAT5/UDP-GlcNButAz **6** are a selective enzyme–substrate pair, mainly driven by the negligible activity of BH-MGAT5 toward UDP-GlcNAc **1** compared to WT-MGAT5.

### BH-MGAT5 Bioorthogonally Tags Substrate Glycoproteins

**Figure 4:**
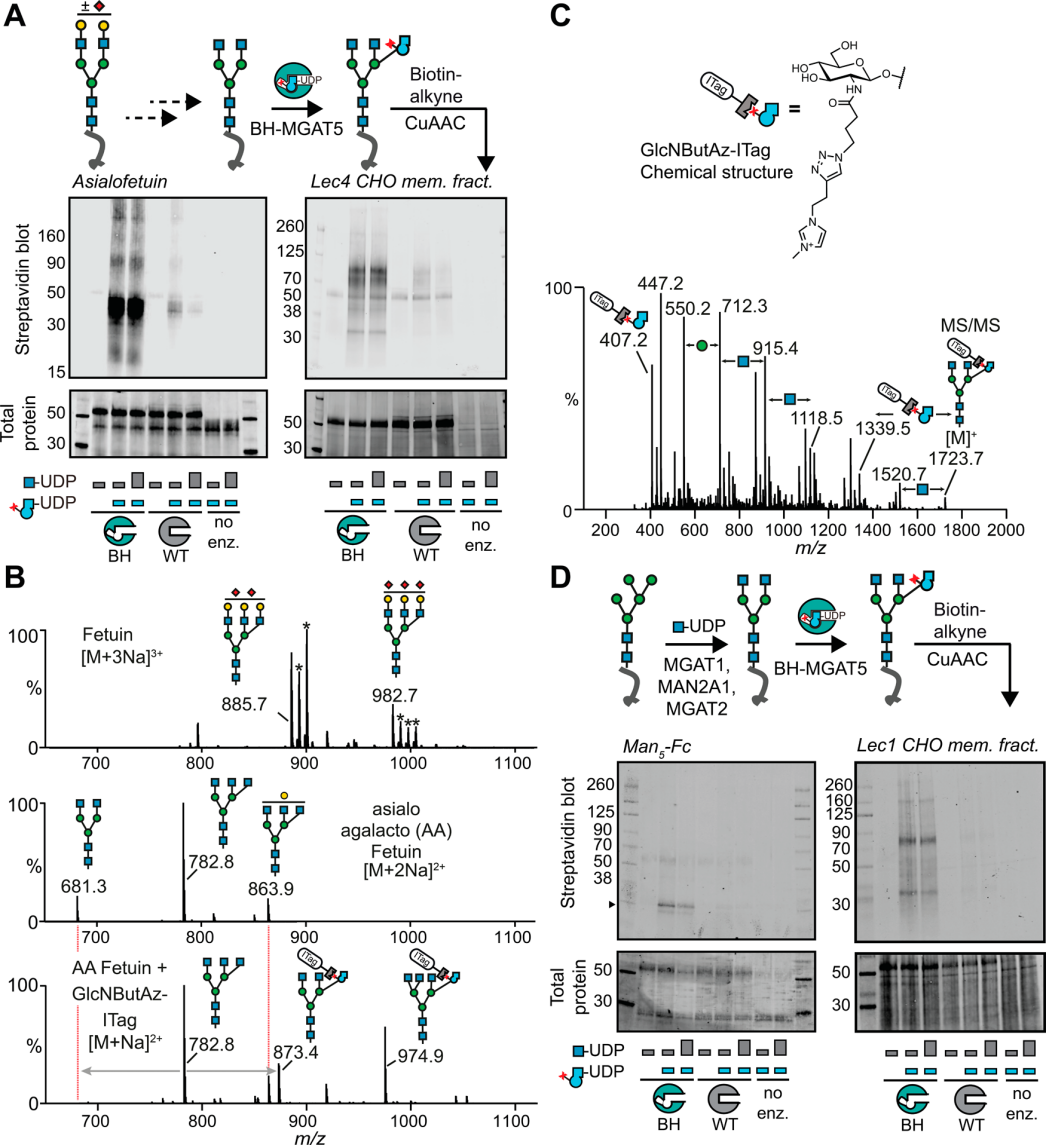
BH-MGAT5 bioorthogonally tags substrate glycoproteins. (A) *In vitro* glycosylation of glycoproteins containing remodeled complex N-glycans, as assessed by streptavidin blot after CuAAC with biotin-alkyne. Left, *in vitro* glycosylation of asialo-agalactofetuin. Right, *in vitro* glycosylation of a desialylated, degalactosylated membrane protein fraction of Lec4 CHO cells. Data are from one out of two independent replicates. (B) Released N-glycan MS spectra of fetuin (top), asialo-agalactofetuin (middle) and GlcNButAz-ITag click-reacted glycans (bottom). Asterisks denote an additional sodium. Data are from one experiment. (C) MS/MS fragmentation spectrum of modified N-glycan Man_3_GlcNAc_4_GlcNButAz-ITag (1723.7 *m*/*z*) showing incorporation of GlcNButAz-ITag. The 407.2 *m*/*z* fragment ion was diagnostic of the GlcNButAz-ITag and was only observed in the modified N-glycan MS/MS spectra (see also Supporting Figure 6C). Data are from one experiment. (D) *In vitro* glycosylation of glycoproteins containing remodeled pentamannosyl N-glycans, as assessed by streptavidin blot after CuAAC with biotin-alkyne. Left, *in vitro* glycosylation of a remodeled Man_5_-Fc protein (arrowhead).^66^ Right, *in vitro* glycosylation of a remodeled membrane protein fraction of Lec1 CHO cells. WT- or BH-MGAT5 reaction mixtures contained 0.2 mM UDP-GlcNButAz **6** and/or either 0.2 mM (small bar) or 0.8 mM (big bar) UDP-GlcNAc. Data are from one out of two independent experiments.

We next assessed whether BH-MGAT5 selectively incorporates GlcNButAz **6** into suitable glycoprotein acceptor substrates. Bovine fetuin contains three N-linked glycosylation sites at Asn99, Asn156, and Asn176, and each is occupied with either a biantennary or a triantennary glycan.^57^ Commercially available asialofetuin was initially employed as a model glycoprotein containing nonsialylated, galactose-terminating N-glycans. Treatment with β-galactosidase remodeled N-glycans into the NGA2 structure that served as an acceptor substrate for MGAT5.^58^ This asialo-agalactofetuin was incubated with BH-MGAT5/UDP-GlcNButAz **6** as well as increasing concentrations of UDP-GlcNAc to mimic the conditions in the secretory pathway (Figure 4A). Treatment with biotin-alkyne under copper-catalyzed azide–alkyne cycloaddition (CuAAC) conditions and streptavidin blot was used to assess BH-MGAT5 activity (Figure 4A, Left). Efficient incorporation of GlcNButAz into asialo-agalactofetuin by BH-MGAT5 was observed, independent of the concentration of UDP-GlcNAc in the reaction mixture. In contrast, WT-MGAT5 exhibited only trace incorporation when UDP-GlcNButAz **6** and UDP-GlcNAc were provided in a 1:1 ratio. A 4-fold excess of UDP-GlcNAc further reduced this signal, confirming that WT-MGAT5 prefers UDP-GlcNAc as a substrate. We then assessed incorporation in a more complex glycoprotein sample. The sialidase/β-galactosidase glycan remodeling regime was applied to a membrane protein fraction of the Lec4 Chinese hamster ovary (CHO) cell variant that lacks intrinsic MGAT5 activity.^59−61^ Glycosylation *in vitro* with BH-MGAT5/UDP-GlcNButAz **6**, subsequent CuAAC with biotin-alkyne and streptavidin blot revealed robust biotin labeling of a range of glycoproteins (Figure 4A, Right). An excess of UDP-GlcNAc did not abrogate this signal, highlighting the selectivity of BH-MGAT5 for UDP-GlcNButAz **6**. In contrast, as seen above, WT-MGAT5 showed residual biotin labeling with a 1:1 UDP-GlcNButAz **6**/UDP-GlcNAc ratio that was further substantially reduced when UDP-GlcNAc was at a 4-fold excess. We characterized chemically modified N-glycans released from fetuin preparations by mass spectrometry (MS, Figure 4B). Comparing complex-type N-glycans before (Figure 4B, top) and after desialylation and degalactosylation (Figure 4B, middle) indicated remodeling to both di- and triantennary GlcNAc-terminating core structures. Upon incubation with BH-MGAT5 and UDP-GlcNButAz, GlcNButAz incorporation was tracked by treatment with an alkyne-containing, permanently positively charged imidazolium tag (ITag) previously found by us and others to enhance detection by mass spectrometry.^38,62−64^ CuAAC with ITag-alkyne enabled detection of both tri- and tetra-antennary N-glycan structures containing the newly formed GlcNButAz-α-1–6-Man linkage (Figure 4B,C, Supporting Figure 6C). Treatment with WT-MGAT5 and UDP-GlcNAc instead produced the corresponding nontagged N-glycan core structures (Supporting Figure 5). Tandem MS by collision-induced dissociation produced a signature ion at 407.2 *m*/*z* corresponding to ITag-linked GlcNButAz, as well as N-glycan fragmentation patterns that differentiate tagged from nontagged GlcNAc moieties (Figure 4C, Supporting Figure 6). Taken together, we concluded that BH-MGAT5 selectively introduces GlcNButAz into glycoprotein substrates, and allows tracking of the newly formed GlcNButAz-α-1–6-Man linkage by MS.

We next showed that BH-MGAT5 can be employed to tag glycoproteins within an assembly line of N-glycan elaboration *in vitro*. A recombinant monomeric antibody Fc fragment containing a single pentamannosyl N-glycan per polypeptide was used as a substrate glycoprotein.^66^ Treatment with recombinantly expressed MGAT1, MAN2A1,^67^ and MGAT2 led to a substrate glycoprotein carrying the NGA2 structure that could be elaborated with BH-MGAT5/UDP-GlcNButAz **6** (Supporting Figure 7).

Subsequent CuAAC with biotin-alkyne followed by streptavidin blot indicated specific chemical tagging of the Fc protein by BH-MGAT5, but not WT-MGAT5 (Figure 4D, Left). The same remodeling approach was followed using a membrane protein fraction of the Lec1 CHO cell line that lacks MGAT1 activity and displays pentamannosylated N-glycans.^59,61^ Incorporation of GlcNButAz into glycoproteins by BH-MGAT5 was confirmed, which was not outcompeted by an excess of UDP-GlcNAc (Figure 4D, Right). The use of WT-MGAT5 led to a low background signal that was abrogated by an excess of UDP-GlcNAc in the reaction mixture. These data indicated that BH-MGAT5 recapitulates the activity of the WT enzyme within an assembly line of N-glycan elaboration.

### Structural Basis of MGAT5 BH Engineering

**Figure 5:**
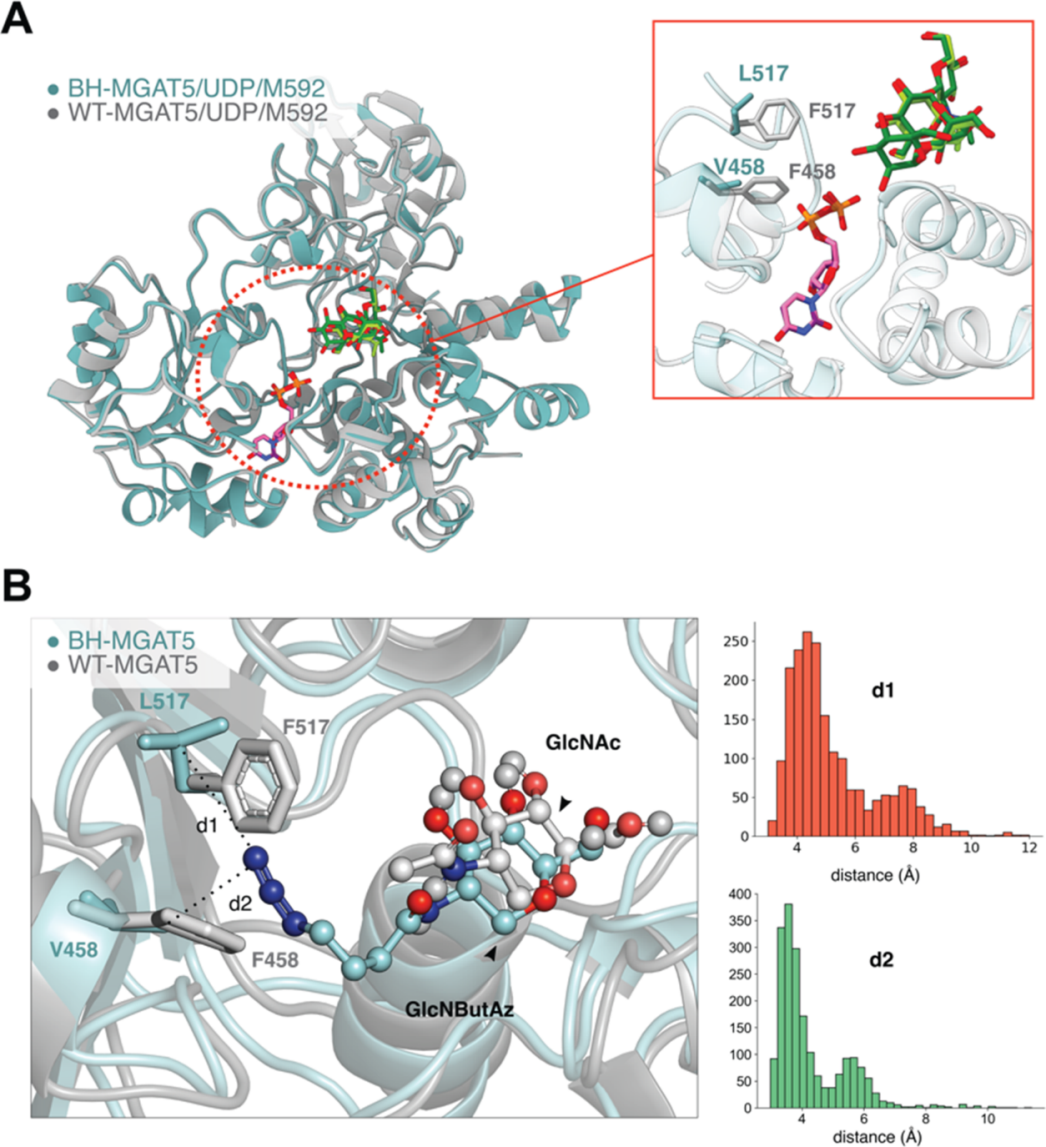
Structural basis of MGAT5 BH engineering. (A) crystal structure of BH-MGAT5 triple mutant^E297A/F458V/F517L^ ternary complex with UDP and M592 (teal) overlaid with WT-MGAT5/UDP/M592 (PDB 6YJU).^20^ Insert: active site and ligands. Gatekeeper residues in WT- and BH-MGAT5 are displayed. UDP bound to WT-MGAT5 and BH-MGAT5 is depicted in purple and pink, respectively. M592 ligand is shown in light green (WT-MGAT5) or dark green (BH-MGAT5). (B) MD simulation to display the active site of BH-MGAT5 in complex with UDP-GlcNButAz **6** and the acceptor M592 (teal backbone). The structure corresponds to the most populated cluster obtained in the MD simulation. The structure of MGAT5 in complex with UDP-GlcNAc and M592 (gray backbone, PDB 6YJU) is shown for comparison. Distribution of the distances d1 and d2 between the terminal nitrogen atom of the ButAz group and the C_γ_ atom of Leu517 and Val458, respectively, in BH-MGAT5, obtained from the MD simulation are shown.

Prior attempts by our group to generate MGAT5/UDP-GlcNAc crystal complexes proved unproductive.^20^ Toward capturing a BH-MGAT5/UDP-GlcNButAz **6**/M592 crystal complex, we crystallized a variant of BH-MGAT5 bearing a substitution of the catalytic Glu297 to Ala to prevent UDP-GlcNButAz **6** turnover without altering the fold of the enzyme. However, soaking experiments using UDP-GlcNButAz **6** ± M592 failed to yield any structures of the binary or ternary complexes. To understand how the BH mutations are positioned within the active site, we solved a crystal structure of the triple MGAT5 variant E297A/F458V/F517L in complex with UDP and M592 (Supporting Information). This structure closely aligned with the corresponding WT-MGAT5 ternary complex (Figure 5A), with a root-mean-square deviation of 0.49 Å over the entire protein (988 aligned residues for chains A and B, superposed by Cα atoms). The gatekeeper residues in WT-MGAT5, Phe458 and Phe517 overlaid directly with the BH-MGAT5 variant residues, Val458 and Leu517 (Figure 5A, insert). While the latter amino acid side chains adopted a similar spatial trajectory as the parental Phe side chains, the space occupied is substantially smaller, enlarging the active site as a defining feature of BH engineering.

Our previous WT-MGAT5 structural campaign revealed flexibility of the enzyme especially upon sequential substrate binding. To assess the basis for UDP-sugar binding, we employed computational modeling to place UDP-GlcNButAz **6** into the active site of BH-MGAT5. MD simulations (500 ns) were performed to assess the structural flexibility in BH-MGAT5 (Supporting Information). We found that the azide group is preferentially oriented toward F458V and F517L, well positioned in the cavity created by the BH substitutions (Figure 5B). To further investigate the conformations adopted by the 4-azidobutyramide group, we analyzed the MD trajectory with a clustering algorithm. The 4-azidobutyramide is oriented toward the variant residues in the most populated clusters. The average distance between the terminal nitrogen and the γ-C atom of Leu517 (d1) and Val458 (d2) is 4.64 and 3.50 Å, respectively (Figure 5B). In contrast, a minor population presents the azide chain facing away from Val458 (Supporting Figures 8 and 9). This conformation of the 4-azidobutyramide might fit in the active site of WT-MGAT5, which would explain why the WT enzyme displays some affinity for UDP-GlcNButAz **6**. A combination of X-ray crystallography and computational modeling thus obtained valuable molecular insight into the structural basis of MGAT5 bump-and-hole engineering.

## Conclusions

Understanding the roles of individual GTs in physiology is hampered by the complex biosynthetic dependencies in the secretory pathway. Genome engineering has produced a large panel of knockout cell lines for the genes encoding GTs including *MGAT5*, revealing implications on the role of the 6-GlcNAc antenna in skin growth, viral infection and chimeric antigen receptor function.^68−71^ Through biochemical studies, the importance of individual MGAT5 domains for substrate binding has been elucidated.^72^ While methods and instrumentation in mass spectrometry have rapidly advanced to produce comprehensive N-glycoprotein data sets, detecting and distinguishing complex N-glycans such as those modified by MGAT5 is still challenging. Chemical, bioorthogonal tools are suitable to enrich glycoproteins for ensuing mass spectrometry but classically display little glycan specificity. Through bump-and-hole engineering, we have previously tailored such tools to be specific reporters of GT activity for Ser/Thr (O)-linked glycans.^36−38,64,73^ Applying the tactic to GTs biosynthesizing N-linked glycans was out of reach until recently because of the absence of structural data depicting the presence of UDP-GlcNAc in their active sites, and because of a lack of a suitably sized collection of bumped UDP-GlcNAc analogs.

Innovations in both the structural biology of MGAT5 and the chemoenzymatic assembly of nucleotide-sugars were crucial prerequisites for success. Our work featured the use of promiscuous biosynthetic enzymes NahK and AGX1^F383A^ as a highly useful OPME system. Design of the BH-engineered active site was aided by structural considerations in a process that highlights the importance of both crystallography and computational simulations. We anticipate that the structural investigation of UDP-sugar binding in the BH-MGAT5 variant will aid future bump-and-hole campaigns for other glycosyltransferases.

In our previous glycosyltransferase BH work, selectivity in an orthogonal enzyme–substrate pair was usually driven by the incompatibility of the WT enzyme with chemically modified UDP-sugar.^36−38^ In addition, we have frequently observed that engineered glycosyltransferases lose activity toward the smaller, native substrate, presumably due to the loss of hydrophobic interactions in the enlarged active site.^38,40^ In contrast, seminal work by Qasba, Hsieh-Wilson and colleagues to engineer the galactosyltransferase B4GALT1 largely retained acceptance of the native nucleotide-sugar.^74,75^ From *in vitro* end point enzymatic assays, we discovered that WT-MGAT5 displays considerable catalytic activity toward donor analogs with various modifications at the acetamide position, including linear azides and alkynes. These data are consistent with the finding that WT-MGAT5 accepted a diazirine-containing UDP-GlcNAc analog in cells by Kohler and colleagues.^34^ Our MD simulations posit that WT-MGAT5 binds such analogs in a conformation in which the acylamide side chain points away from the residues Phe458 and Phe517. While enabling enzymatic activity, this substrate conformation appears to be catalytically less productive than the conformation facing those residues, leading to reduced conversion *in vitro*. In contrast, our BH-MGAT5 variant displayed increased acceptance of UDP-GlcNButAz **6** while completely losing activity against UDP-GlcNAc **1**.

We are mindful that the terminology of “gatekeeper” residues that is normally used in BH engineering may not be applicable in the classical sense herein, since engineering appears to restrict rather than expand the substrate profile. However, the term is used since engineering does improve acceptance of bumped substrate **6** in end point assays and kinetic measurements. Furthermore, WT-MGAT5 did not accept UDP-GlcNButAz **6** to a notable degree in the presence of the better substrate UDP-GlcNAc, suggesting specificity of the BH system in a biological setting.

We note that the affinity of WT-MGAT5 for its substrate UDP-GlcNAc is likely among the lowest of the human GlcNAc transferases, with a *K*_*M*_ in the millimolar range.^20,22^ This low affinity is replicated in our BH system, which introduces the challenge in cellular application that background incorporation of GlcNButAz by other transferases may be observed. Our choice of MGAT5 as a target transferase was inspired by the possibilities offered through extensive structural data, despite the inherently low acceptance of UDP-GlcNAc. Exoenzymatic glycan remodeling has been successfully applied to install chemical functionality into cell surface glycans, as a potential application of BH-MGAT5 that does not involve cellular delivery of the enzyme.^76,77^ However, the BH approach is also uniquely suited to tailoring a substrate to the active site of an enzyme, which can lead to a further increase of affinity over the native enzyme–substrate pair.^40^ We expect that our foundational work on a functional BH system will allow us to address the implications of substrate affinity and related questions on the physiological activity of MGAT5. We have gained valuable insights into engineering GlcNAc transferases that will be translatable to similar enzymes in the expansion of our glycan-based chemical toolbox.

## Data Availability

The crystal structure has been submitted to the Protein Data Bank under accession code 9F5H. Data for this manuscript are available in the main text and Supporting Information. Raw data underlying the manuscript include enzymatic conversion data, raw data of gels and blots, and compound characterization data. These are securely deposited at the Francis Crick Institute and available upon request.

## Supporting Information Available

Additional experimental details, data, and figures and tables for chemical synthesis and compound characterization, *in vitro* glycosylation assays, X-ray crystallography, analysis of protein atom root-mean-square displacements during the MD simulation, expression and purification of recombinant enzymes, HPLC and NMR spectra, and supporting references (PDF)

## References

[ref1] Sosicka P.; Ng B. G.; Freeze H. H. Congenital Disorders of Glycosylation. Compr. Glycosci. 2021, 6 (24), 294–334. 10.1016/B978-0-12-819475-1.00013-4.

[ref2] Ye Z.; Marth J. D. N-Glycan Branching Requirement in Neuronal and Postnatal Viability. Glycobiology 2004, 14 (6), 547–558. 10.1093/glycob/cwh069. 15044398

[ref3] Stanley P.; Moremen K. W.; Lewis N. E.; Taniguchi N.; Aebi M. N-Glycans. In Essentials of Glycobiology, 4th ed.; Cold Spring Harbor Laboratory Press, 2022; pp 487–494 10.1101/GLYCOBIOLOGY.4E.9.

[ref4] Reily C.; Stewart T. J.; Renfrow M. B.; Novak J. Glycosylation in Health and Disease. Nat. Rev. Nephrol. 2019, 15 (6), 346–366. 10.1038/s41581-019-0129-4. 30858582 PMC6590709

[ref5] de-Souza-Ferreira M.; Ferreira É. E.; de-Freitas-Junior J. C. M. Aberrant N-Glycosylation in Cancer: MGAT5 and β1,6-GlcNAc Branched N-Glycans as Critical Regulators of Tumor Development and Progression. Cell Oncol. 2023, 46 (3), 481–501. 10.1007/s13402-023-00770-4. PMC12974675 36689079

[ref6] Mendelsohn R.; Cheung P.; Berger L.; Partridge E.; Lau K.; Datti A.; Pawling J.; Dennis J. W. Complex N-Glycan and Metabolic Control in Tumor Cells. Cancer Res. 2007, 67 (20), 9771–9780. 10.1158/0008-5472.CAN-06-4580. 17942907

[ref7] Granovsky M.; Fata J.; Pawling J.; Muller W. J.; Khokha R.; Dennis J. W. Suppression of Tumor Growth and Metastasis in Mgat5-Deficient Mice. Nat. Med. 2000, 6 (3), 306–312. 10.1038/73163. 10700233

[ref8] Dimitroff C. J. I-Branched Carbohydrates as Emerging Effectors of Malignant Progression. Proc. Natl. Acad. Sci. U.S.A. 2019, 116 (28), 13729–13737. 10.1073/pnas.1900268116. 31213534 PMC6628663

[ref9] Scott D. A.; Casadonte R.; Cardinali B.; Spruill L.; Mehta A. S.; Carli F.; Simone N.; Kriegsmann M.; Del Mastro L.; Kriegsmann J.; Drake R. R. Increases in Tumor N-Glycan Polylactosamines Associated with Advanced HER2-Positive and Triple-Negative Breast Cancer Tissues. Proteomics: Clin. Appl. 2019, 13 (1), e1800014 10.1002/prca.201800014. 30592377 PMC8913074

[ref10] Lagana A.; Goetz J. G.; Cheung P.; Raz A.; Dennis J. W.; Nabi I. R. Galectin Binding to Mgat5-Modified N-Glycans Regulates Fibronectin Matrix Remodeling in Tumor Cells. Mol. Cell. Biol. 2006, 26 (8), 3181–3193. 10.1128/MCB.26.8.3181-3193.2006. 16581792 PMC1446937

[ref11] Dennis J. W.; Taniguchi N.; Pierce M. Mannosyl (Alpha-1,6-)-Glycoprotein Beta-1,6-N-Acetyl-Glucosaminyltransferase (MGAT5). Handb. Glycosyltransferases Relat. Genes 2014, 1, 233–246. 10.1007/978-4-431-54240-7_15/FIGURES/2.

[ref12] Cummings R. D.; Trowbridge I. S.; Kornfeld S. A Mouse Lymphoma Cell Line Resistant to the Leukoagglutinating Lectin from Phaseolus Vulgaris Is Deficient in UDP-GlcNAc: Alpha-D-Mannoside Beta 1,6 N-Acetylglucosaminyltransferase. J. Biol. Chem. 1982, 257 (22), 13421–13427. 10.1016/S0021-9258(18)33465-3. 6216250

[ref13] Brockhausen I.; Narasimhan S.; Schachter H. The Biosynthesis of Highly Branched N-Glycans: Studies on the Sequential Pathway and Functional Role of N-Actylglucosaminyltransferases I, II, III, IV, V and VI. Biochimie 1988, 70 (11), 1521–1533. 10.1016/0300-9084(88)90289-1. 2977290

[ref14] Murata K.; Miyoshi E.; Kameyama M.; Ishikawa O.; Kabuto T.; Sasaki Y.; Hiratsuka M.; Ohigashi H.; Ishiguro S.; Ito S.; Honda H.; Takemura F.; Taniguchi N.; Imaoka S. Expression of N-Acetylglucosaminyltransferase V in Colorectal Cancer Correlates with Metastasis and Poor Prognosis. Clin. Cancer Res. 2000, 6 (5), 1772–1777. 10815896

[ref15] Nishikawa A.; Gu J.; Fujii S.; Taniguchi N. Determination of N-Acetylglucosaminyltransferases III, IV and V in Normal and Hepatoma Tissues of Rats. Biochim. Biophys. Acta 1990, 1035 (3), 313–318. 10.1016/0304-4165(90)90094-D. 2145037

[ref16] Palcic M. M.; Heerze L. D.; Pierce M.; Hindsgaul O. The Use of Hydrophobic Synthetic Glycosides as Acceptors in Glycosyltransferase Assays. Glycoconj. J. 1988, 5 (1), 49–63. 10.1007/BF01048331.

[ref17] Palcic M. M.; Ripka J.; Kaur K. J.; Shoreibah M.; Hindsgaul O.; Pierce M. Regulation of N-Acetylglucosaminyltransferase V Activity: Kinetic Comparisons of Parental, Rous Sarcoma Virus-Transformed Bhk, and L-Phytohemagglutinin-Resistant BHK Cells Using Synthetic Substrates and an Inhibitory Substrate Analog. J. Biol. Chem. 1990, 265 (12), 6759–6769. 10.1016/S0021-9258(19)39214-2. 2157709

[ref18] Pierce M.; Arango J.; Tahir S. H.; Hindsgaul O. Activity of UDP-GlcNAc: α-Mannoside β(1,6)N-Acetylglucosaminyltransferase (GnT V) in Cultured Cells Using a Synthetic Trisaccharide Acceptor. Biochem. Biophys. Res. Commun. 1987, 146 (2), 679–684. 10.1016/0006-291X(87)90582-1. 2956949

[ref19] Brockhausen I.; Carver J. P.; Schachter H. Control of Glycoprotein Synthesis. The Use of Oligosaccharide Substrates and HPLC to Study the Sequential Pathway for N-Acetylglucosaminyltransferases I, II, III, IV, V, and VI in the Biosynthesis of Highly Branched N-Glycans by Hen Oviduct Membranes. Biochem. Cell Biol. 1988, 66 (10), 1134–1151. 10.1139/o88-131. 2975180

[ref20] Darby J. F.; Gilio A. K.; Piniello B.; Roth C.; Blagova E.; Hubbard R. E.; Rovira C.; Davies G. J.; Wu L. Substrate Engagement and Catalytic Mechanisms of N-Acetylglucosaminyltransferase V. ACS Catal. 2020, 10 (15), 8590–8596. 10.1021/acscatal.0c02222.

[ref21] Nagae M.; Kizuka Y.; Mihara E.; Kitago Y.; Hanashima S.; Ito Y.; Takagi J.; Taniguchi N.; Yamaguchi Y. Structure and Mechanism of Cancer-Associated N-Acetylglucosaminyltransferase-V. Nat. Commun. 2018, 9 (1), 3380 10.1038/s41467-018-05931-w. 30140003 PMC6107550

[ref22] Vibhute A. M.; Tanaka H. nori.; Mishra S. K.; Osuka R. F.; Nagae M.; Yonekawa C.; Korekane H.; Doerksen R. J.; Ando H.; Kizuka Y. Structure-Based Design of UDP-GlcNAc Analogs as Candidate GnT-V Inhibitors. Biochim. Biophys. Acta, Gen. Subj. 2022, 1866 (6), 130118 10.1016/J.BBAGEN.2022.130118. 35248671 PMC9947920

[ref23] Brockhausen I.; Reck F.; Kuhns W.; Khan S.; Matta K. L.; Meinjohanns E.; Paulsen H.; Shah R. N.; Baker M. A.; Schachter H. Substrate Specificity and Inhibition of UDP-GlcNAc:GlcNAcβ1–2Manα1–6R B1,6-N-Acetylglucosaminyltransferase V Using Synthetic Substrate Analogues. Glycoconj. J. 1995, 12 (3), 371–379. 10.1007/BF00731340/METRICS. 7496152

[ref24] Reck F.; Meinjohanns E.; Tan J.; Grey A. A.; Paulsen H.; Schachter H. Synthesis of Pentasaccharide Analogues of the N-Glycan Substrates of N-Acetylglucosaminyltransferases III, IV and V Using Tetrasaccharide Precursors and Recombinant β-(1 → 2)-N-Acetylglucosaminyltransferase II. Carbohydr. Res. 1995, 275 (2), 221–229. 10.1016/0008-6215(95)00091-7. 8529222

[ref25] Brockhausen I.; Möller G.; Yang J. M.; Khan S. H.; Matta K. L.; Paulsen H.; Grey A. A.; Shah R. N.; Schachter H. Control of Glycoprotein Synthesis. Characterization of (1 → 4)-N-Acetyl-β-d-Glucosaminyltransferases Acting on the α-D-(1 → 3)- and α-D-(1 → 6)-Linked Arms of N-Linked Oligosaccharides. Carbohydr. Res. 1992, 236 (C), 281–299. 10.1016/0008-6215(92)85022-R. 1291052

[ref26] Khan S. H.; Abbas S. A.; Matta K. L. Synthesis of Some Oligosaccharides Containing the O-(2-Acetamido-2-Deoxy-β-D-Glucopyranosyl)-(1→2)-O-α-D-Mannopyranosyl Unit. Potential Substrates for UDP-GlcNAc: α-D-Mannopyranosyl-(1→6)-N-Acetyl-β-D-Glucosaminyl-Transferase (GnT-V). Carbohydr. Res. 1989, 193 (C), 125–139. 10.1016/0008-6215(89)85112-2. 2532953

[ref27] Khan S. H.; Matta K. L. Synthesis of 2-Acetamido-2-Deoxy-β-D-Glucopyranosyl-(1 → 2)-α-D-Mannopyranosyl (1 → 6)-β-D-Mannopyranosyl-(1 → 4)-2-Acetamido-2-Deoxy-D-Glucopyranose. Acceptor-Substrate Recognition by N-Acetylglucosaminyltransferase-V (GnT-V). Carbohydr. Res. 1995, 278 (2), 351–362. 10.1016/0008-6215(95)00257-X. 8590449

[ref28] Khan S. H.; Matta K. L. Synthesis of 4-Nitrophenyl O-(2-Acetamido-2-Deoxy-β-D-Glucopyranosyl)-(1 → 2)-O-(6-O-Methyl-α-D-Mannopyranosyl)-(1 → 6)-β-D-Glucopyranoside and Its 4′,6′-Di-O-Methyl Analog. Potential Inhibitors of N-Acetylglucosaminyl-Transferase V (GnT-V). Carbohydr. Res. 1993, 243 (1), 29–42. 10.1016/0008-6215(93)84079-L. 8324765

[ref29] Khan S. H.; Abbas S. A.; Matta K. L. Synthesis of 4-NitrophenylO-(2-Acetamido-2-Deoxy-β-D-Glucopyranosyl)-(1 → 2)-O-(4-O-Methyl-α-D-Mannopyranosyl)-(1 → 6)-β-D-Glucopyranoside. A Potential Specific Acceptor-Substrate ForN-Acetylglucosaminyltransferase-V (GnT V). Carbohydr. Res. 1990, 205 (C), 385–397. 10.1016/0008-6215(90)80156-W. 2148907

[ref30] Parker C. G.; Pratt M. R. Click Chemistry in Proteomic Investigations. Cell 2020, 180, 605–632. 10.1016/j.cell.2020.01.025. 32059777 PMC7087397

[ref31] Zol-Hanlon M. I.; Schumann B. Open Questions in Chemical Glycobiology. Commun. Chem. 2020, 3 (1), 102 10.1038/s42004-020-00337-6. 33748433 PMC7610353

[ref32] Vocadlo D. J.; Hang H. C.; Kim E. J.; Hanover J. A.; Bertozzi C. R. A Chemical Approach for Identifying O-GlcNAc-Modified Proteins in Cells. Proc. Natl. Acad. Sci. U.S.A. 2003, 100 (16), 9116–9121. 10.1073/pnas.1632821100. 12874386 PMC171382

[ref33] Kufleitner M.; Haiber L. M.; Wittmann V. Metabolic Glycoengineering – Exploring Glycosylation with Bioorthogonal Chemistry. Chem. Soc. Rev. 2023, 52 (2), 510–535. 10.1039/D2CS00764A. 36537135

[ref34] Wu H.; Shajahan A.; Yang J. Y.; Capota E.; Wands A. M.; Arthur C. M.; Stowell S. R.; Moremen K. W.; Azadi P.; Kohler J. J. A Photo-Cross-Linking GlcNAc Analog Enables Covalent Capture of N-linked Glycoprotein-Binding Partners on the Cell Surface. Cell Chem. Biol. 2022, 29 (1), 84–97.e8. 10.1016/j.chembiol.2021.07.007. 34331854 PMC8792112

[ref35] Islam K. The Bump-and-Hole Tactic: Expanding the Scope of Chemical Genetics. Cell Chem. Biol. 2018, 25 (10), 1171–1184. 10.1016/j.chembiol.2018.07.001. 30078633 PMC6195450

[ref36] Cioce A.; Malaker S. A.; Schumann B. Generating Orthogonal Glycosyltransferase and Nucleotide Sugar Pairs as Next-Generation Glycobiology Tools. Curr. Opin. Chem. Biol. 2021, 60, 66–78. 10.1016/j.cbpa.2020.09.001. 33125942 PMC7955280

[ref37] Schumann B.; Malaker S. A.; Wisnovsky S. P.; Debets M. F.; Agbay A. J.; Fernandez D.; Wagner L. J. S.; Lin L.; Li Z.; Choi J.; Fox D. M.; Peh J.; Gray M. A.; Pedram K.; Kohler J. J.; Mrksich M.; Bertozzi C. R. Bump-and-Hole Engineering Identifies Specific Substrates of Glycosyltransferases in Living Cells. Mol. Cell 2020, 78 (5), 824–834.e15. 10.1016/j.molcel.2020.03.030. 32325029 PMC7276986

[ref38] Li Z.; Vagno L. Di.; Cheallaigh A. N.; Sammon D.; Briggs D. C.; Chung N.; Chang V.; Mahoney K. E.; Cioce A.; Murphy L. D.; Chen Y.-H.; Narimatsu Y.; Miller R. L.; Willems L. I.; Malaker S. A.; Miller G. J.; Hohenester E.; Schumann B.; et al. Xylosyltransferase Bump-and-Hole Engineering to Chemically Manipulate Proteoglycans in Mammalian Cells. bioRxiv 2023, 12, 20 10.1101/2023.12.20.572522.

[ref39] Rodriguez A. C.; Yu S. H.; Li B.; Zegzouti H.; Kohler J. J. Enhanced Transfer of a Photocross-Linking N-Acetylglucosamine (GlcNAc) Analog by an O-GlcNAc Transferase Mutant with Converted Substrate Specificity. J. Biol. Chem. 2015, 290 (37), 22638 10.1074/jbc.M115.667006. 26240142 PMC4566237

[ref40] Choi J.; Wagner L. J. S.; Timmermans S. B. P. E.; Malaker S. A.; Schumann B.; Gray M. A.; Debets M. F.; Takashima M.; Gehring J.; Bertozzi C. R. Engineering Orthogonal Polypeptide GalNAc-Transferase and UDP-Sugar Pairs. J. Am. Chem. Soc. 2019, 141 (34), 13442–13453. 10.1021/jacs.9b04695. 31373799 PMC6813768

[ref41] Cioce A.; Bineva-Todd G.; Agbay A. J.; Choi J.; Wood T. M.; Debets M. F.; Browne W. M.; Douglas H. L.; Roustan C.; Tastan O. Y.; Kjaer S.; Bush J. T.; Bertozzi C. R.; Schumann B. Optimization of Metabolic Oligosaccharide Engineering with Ac4GalNAlk and Ac4GlcNAlk by an Engineered Pyrophosphorylase. ACS Chem. Biol. 2021, 16 (10), 1961–1967. 10.1021/acschembio.1c00034. 33835779 PMC8501146

[ref42] Debets M. F.; Tastan O. Y.; Wisnovsky S. P.; Malaker S. A.; Angelis N.; Moeckl L. K. R.; Choi J.; Flynn H.; Wagner L. J. S.; Bineva-Todd G.; Antonopoulos A.; Cioce A.; Browne W. M.; Li Z.; Briggs D. C.; Douglas H. L.; Hess G. T.; Agbay A. J.; Roustan C.; Kjaer S.; Haslam S. M.; Snijders A. P.; Bassik M. C.; Moerner W. E.; Li V. S. W.; Bertozzi C. R.; Schumann B. Metabolic Precision Labeling Enables Selective Probing of O-Linked N-Acetylgalactosamine Glycosylation. Proc. Natl. Acad. Sci. U.S.A. 2020, 117 (41), 25293–25301. 10.1073/pnas.2007297117. 32989128 PMC7568240

[ref43] González F. V. D. L.; Boddington M. E.; Prindl M. I.; Capicciotti C. J. Glyco-Engineering Cell Surfaces by Exo-Enzymatic Installation of GlcNAz and LacNAz Motifs. bioRxiv 2023, 08, 28 10.1101/2023.08.28.554597. 38394345

[ref44] Guan W.; Cai L.; Wang P. G. Highly Efficient Synthesis of UDP-GalNAc/GlcNAc Analogues with Promiscuous Recombinant Human UDP-GalNAc Pyrophosphorylase AGX1. Chemistry 2010, 16 (45), 13343–13345. 10.1002/chem.201002315. 21031374 PMC3441830

[ref45] Cioce A.; Calle B.; Rizou T.; Lowery S. C.; Bridgeman V. L.; Mahoney K. E.; Marchesi A.; Bineva-Todd G.; Flynn H.; Li Z.; Tastan O. Y.; Roustan C.; Soro-Barrio P.; Rafiee M. R.; Garza-Garcia A.; Antonopoulos A.; Wood T. M.; Keenan T.; Both P.; Huang K.; Parmeggian F.; Snijders A. P.; Skehel M.; Kjær S.; Fascione M. A.; Bertozzi C. R.; Haslam S. M.; Flitsch S. L.; Malaker S. A.; Malanchi I.; Schumann B. Cell-Specific Bioorthogonal Tagging of Glycoproteins. Nat. Commun. 2022, 13 (1), 6237 10.1038/s41467-022-33854-0. 36284108 PMC9596482

[ref46] Jackson E. G.; Cutolo G.; Yang B.; Yarravarapu N.; Burns M. W. N.; Bineva-Todd G.; Roustan C.; Thoden J. B.; Lin-Jones H. M.; van Kuppevelt T. H.; Holden H. M.; Schumann B.; Kohler J. J.; Woo C. M.; Pratt M. R. 4-Deoxy-4-Fluoro-GalNAz (4FGalNAz) Is a Metabolic Chemical Reporter of O-GlcNAc Modifications, Highlighting the Notable Substrate Flexibility of O-GlcNAc Transferase. ACS Chem. Biol. 2022, 17 (1), 159–170. 10.1021/acschembio.1c00818. 34931806 PMC8787749

[ref47] Wen L.; Gadi M. R.; Zheng Y.; Gibbons C.; Kondengaden S. M.; Zhang J.; Wang P. G. Chemoenzymatic Synthesis of Unnatural Nucleotide Sugars for Enzymatic Bioorthogonal Labeling. ACS Catal. 2018, 8 (8), 7659–7666. 10.1021/acscatal.8b02081.

[ref48] Muthana M. M.; Qu J.; Li Y.; Zhang L.; Yu H.; Ding L.; Malekan H.; Chen X. Efficient One-Pot Multienzyme Synthesis of UDP-Sugars Using a Promiscuous UDP-Sugar Pyrophosphorylase from *Bifidobacterium Longum* (BLUSP). Chem. Commun. 2012, 48 (21), 2728–2730. 10.1039/c2cc17577k. 22306833

[ref49] Batt A. R.; Zaro B. W.; Navarro M. X.; Pratt M. R. Metabolic Chemical Reporters of Glycans Exhibit Cell-Type-Selective Metabolism and Glycoprotein Labeling. ChemBioChem 2017, 18 (13), 1177–1182. 10.1002/cbic.201700020. 28231413 PMC5580397

[ref50] Fan X.; Song Q.; Sun D. en.; Hao Y.; Wang J.; Wang C.; Chen X. Cell-Type-Specific Labeling and Profiling of Glycans in Living Mice. Nat. Chem. Biol. 2022, 18 (6), 625–633. 10.1038/s41589-022-01016-4. 35513511

[ref51] Yu S. H.; Boyce M.; Wands A. M.; Bond M. R.; Bertozzi C. R.; Kohler J. J. Metabolic Labeling Enables Selective Photocrosslinking of O-GlcNAc-Modified Proteins to Their Binding Partners. Proc. Natl. Acad. Sci. U.S.A. 2012, 109 (13), 4834–4839. 10.1073/pnas.1114356109. 22411826 PMC3323966

[ref52] Peneff C.; Ferrari P.; Charrier V.; Taburet Y.; Monnier C.; Zamboni V.; Winter J.; Harnois M.; Fassy F.; Bourne Y. Crystal Structures of Two Human Pyrophosphorylase Isoforms in Complexes with UDPGlc(Gal)NAc: Role of the Alternatively Spliced Insert in the Enzyme Oligomeric Assembly and Active Site Architecture. EMBO J. 2001, 20 (22), 6191–6202. 10.1093/emboj/20.22.6191. 11707391 PMC125729

[ref53] Delchev V. B. Computational (DFT and TD DFT) Study of the Electron Structure of the Tautomers/Conformers of Uridine and Deoxyuridine and the Processes of Intramolecular Proton Transfers. J. Mol. Model. 2010, 16 (4), 749–757. 10.1007/s00894-009-0593-z. 19820971

[ref54] Fedeles B. I.; Li D.; Singh V. Structural Insights Into Tautomeric Dynamics in Nucleic Acids and in Antiviral Nucleoside Analogs. Front. Mol. Biosci. 2022, 8, 823253 10.3389/fmolb.2021.823253. 35145998 PMC8822119

[ref55] Islam K.; Chen Y.; Wu H.; Bothwell I. R.; Blum G. J.; Zeng H.; Dong A.; Zheng W.; Min J.; Deng H.; Luo M. Defining efficient enzyme-cofactor pairs for bioorthogonal profiling of protein methylation. Proc. Natl. Acad. Sci. U.S.A. 2013, 110 (42), 16778–16783. 10.1073/pnas.1216365110. 24082136 PMC3801003

[ref56] Gibson B. A.; Zhang Y.; Jiang H.; Hussey K. M.; Shrimp J. H.; Lin H.; Schwede F.; Yu Y.; Kraus W. L. Chemical genetic discovery of PARP targets reveals a role for PARP-1 in transcription elongation. Science 2016, 353 (6294), 45–50. 10.1126/science.aaf7865. 27256882 PMC5540732

[ref57] Lin Y. H.; Franc V.; Heck A. J. R. Similar Albeit Not the Same: In-Depth Analysis of Proteoforms of Human Serum, Bovine Serum, and Recombinant Human Fetuin. J. Proteome Res. 2018, 17 (8), 2861–2869. 10.1021/acs.jproteome.8b00318. 29966421 PMC6079914

[ref58] Mutoh T.; Naoi M.; Sobue I.; Kiuchi K.; Nagatsu T. A Microassay for Acid β-Galactosidase Activity toward Asialofetuin. Clin. Chim. Acta 1985, 152 (3), 307–314. 10.1016/0009-8981(85)90106-8. 2415273

[ref59] Stanley P.; Caillibot V.; Siminovitch L. Selection and Characterization of Eight Phenotypically Distinct Lines of Lectin-Resistant Chinese Hamster Ovary Cells. Cell 1975, 6 (2), 121–128. 10.1016/0092-8674(75)90002-1. 1182798

[ref60] Weinstein J.; Sundaram S.; Wang X.; Delgado D.; Basil R.; Stanley P. A Point Mutation Causes Mistargeting of Golgi GlcNAc-TV in the Lec4A Chinese Hamster Ovary Glycosylation Mutant. J. Biol. Chem. 1996, 271 (44), 27462–27469. 10.1074/JBC.271.44.27462. 8910328

[ref61] North S. J.; Huang H. H.; Sundaram S.; Jang-Lee J.; Etienne A. T.; Trollope A.; Chalabi S.; Dell A.; Stanley P.; Haslam S. M. Glycomics Profiling of Chinese Hamster Ovary Cell Glycosylation Mutants Reveals N-Glycans of a Novel Size and Complexity. J. Biol. Chem. 2010, 285 (8), 5759–5775. 10.1074/jbc.M109.068353. 19951948 PMC2820803

[ref62] Galan M. C.; Tran A. T.; Bernard C. Ionic-Liquid-Based Catch and Release Mass Spectroscopy Tags for Enzyme Monitoring. Chem. Commun. 2010, 46 (47), 8698–8970. 10.1039/C0CC04224B. 20976336

[ref63] Calle B.; Bineva-Todd G.; Marchesi A.; Flynn H.; Ghirardello M.; Tastan O. Y.; Roustan C.; Choi J.; Galan M. C.; Schumann B.; Malaker S. A. Benefits of Chemical Sugar Modifications Introduced by Click Chemistry for Glycoproteomic Analyses. J. Am. Soc. Mass Spectrom. 2021, 32 (9), 2366–2375. 10.1021/jasms.1c00084. 33871988 PMC7611619

[ref64] Gonzalez-Rodriguez E.; Zol-Hanlon M.; Bineva-Todd G.; Marchesi A.; Skehel M.; Mahoney K. E.; Roustan C.; Borg A.; Di Vagno L.; Kjær S.; Wrobel A. G.; Benton D. J.; Nawrath P.; Flitsch S. L.; Joshi D.; González-Ramírez A. M.; Wilkinson K. A.; Wilkinson R. J.; Wall E. C.; Hurtado-Guerrero R.; Malaker S. A.; Schumann B. O-Linked Sialoglycans Modulate the Proteolysis of SARS-CoV-2 Spike and Likely Contribute to the Mutational Trajectory in Variants of Concern. ACS Cent. Sci. 2023, 9 (3), 393–404. 10.1021/acscentsci.2c01349. 36968546 PMC10037455

[ref66] Makrydaki E.; Donini R.; Krueger A.; Royle K.; Moya Ramirez I.; Kuntz D. A.; Rose D. R.; Haslam S. M.; Polizzi K. M.; Kontoravdi C. Immobilized Enzyme Cascade for Targeted Glycosylation. Nat. Chem. Biol. 2024, 20, 732–741. 10.1038/s41589-023-01539-4. 38321209 PMC11142912

[ref67] Armstrong Z.; Kuo C. L.; Lahav D.; Liu B.; Johnson R.; Beenakker T. J. M.; De Boer C.; Wong C. S.; Van Rijssel E. R.; Debets M. F.; Florea B. I.; Hissink C.; Boot R. G.; Geurink P. P.; Ovaa H.; Van Der Stelt M.; Van Der Marel G. M.; Codée J. D. C.; Aerts J. M. F. G.; Wu L.; Overkleeft H. S.; Davies G. J. Manno- Epi-Cyclophellitols Enable Activity-Based Protein Profiling of Human α-Mannosidases and Discovery of New Golgi Mannosidase II Inhibitors. J. Am. Chem. Soc. 2020, 142 (30), 13021–13029. 10.1021/jacs.0c03880. 32605368

[ref68] Dabelsteen S.; Pallesen E. M. H.; Marinova I. N.; Nielsen M. I.; Adamopoulou M.; Rømer T. B.; Levann A.; Andersen M. M.; Ye Z.; Thein D.; Bennett E. P.; Büll C.; Moons S. J.; Boltje T.; Clausen H.; Vakhrushev S. Y.; Bagdonaite I.; Wandall H. H. Essential Functions of Glycans in Human Epithelia Dissected by a CRISPR-Cas9-Engineered Human Organotypic Skin Model. Dev. Cell 2020, 54 (5), 669 10.1016/j.devcel.2020.06.039. 32710848 PMC7497784

[ref69] Bagdonaite I.; Marinova I. N.; Rudjord-Levann A. M.; Pallesen E. M. H.; King-Smith S. L.; Karlsson R.; Rømer T. B.; Chen Y. H.; Miller R. L.; Olofsson S.; Nordén R.; Bergström T.; Dabelsteen S.; Wandall H. H. Glycoengineered Keratinocyte Library Reveals Essential Functions of Specific Glycans for All Stages of HSV-1 Infection. Nat. Commun. 2023, 14 (1), 7000 10.1038/s41467-023-42669-6. 37919266 PMC10622544

[ref70] Yang Z.; Wang S.; Halim A.; Schulz M. A.; Frodin M.; Rahman S. H.; Vester-Christensen M. B.; Behrens C.; Kristensen C.; Vakhrushev S. Y.; Bennett E. P.; Wandall H. H.; Clausen H. Engineered CHO Cells for Production of Diverse, Homogeneous Glycoproteins. Nat. Biotechnol. 2015, 33 (8), 842–844. 10.1038/nbt.3280. 26192319

[ref71] De Bousser E.; De; Festjens N.; Meuris L.; Plets E.; Hecke A.; Van; Wyseure E.; Munter S. De.; Vandekerckhove B.; Callewaert N. N-Glycosylation Engineering in Chimeric Antigen Receptor T Cells Enhances Anti-Tumor Activity. bioRxiv 2023, 2023.01.23 10.1101/2023.01.23.525164.

[ref72] Osuka R. F.; Hirata T.; Nagae M.; Nakano M.; Shibata H.; Okamoto R.; Kizuka Y. N-Acetylglucosaminyltransferase-V Requires a Specific Noncatalytic Luminal Domain for Its Activity toward Glycoprotein Substrates. J. Biol. Chem. 2022, 298 (3), 101666 10.1016/j.jbc.2022.101666. 35104505 PMC8889256

[ref73] Scott E.; Hodgson K.; Calle B.; Turner H.; Cheung K.; Bermudez A.; Marques F. J. G.; Pye H.; Yo E. C.; Islam K.; Oo H. Z.; McClurg U. L.; Wilson L.; Thomas H.; Frame F. M.; Orozco-Moreno M.; Bastian K.; Arredondo H. M.; Roustan C.; Gray M. A.; Kelly L.; Tolson A.; Mellor E.; Hysenaj G.; Goode E. A.; Garnham R.; Duxfield A.; Heavey S.; Stopka-Farooqui U.; Haider A.; Freeman A.; Singh S.; Johnston E. W.; Punwani S.; Knight B.; McCullagh P.; McGrath J.; Crundwell M.; Harries L.; Bogdan D.; Westaby D.; Fowler G.; Flohr P.; Yuan W.; Sharp A.; de Bono J.; Maitland N. J.; Wisnovsky S.; Bertozzi C. R.; Heer R.; Guerrero R. H.; Daugaard M.; Leivo J.; Whitaker H.; Pitteri S.; Wang N.; Elliott D. J.; Schumann B.; Munkley J. Upregulation of GALNT7 in Prostate Cancer Modifies O-Glycosylation and Promotes Tumour Growth. Oncogene 2023, 42 (12), 926–937. 10.1038/s41388-023-02604-x. 36725887 PMC10020086

[ref74] Ramakrishnan B.; Qasba P. K. Structure-based Design of β1,4-Galactosyltransferase I (β4Gal-T1) with Equally Efficient N-Acetylgalactosaminyltransferase Activity. J. Biol. Chem. 2002, 277 (23), 20833–20839. 10.1074/jbc.M111183200. 11916963

[ref75] Khidekel N.; Arndt S.; Lamarre-Vincent N.; Lippert A.; Poulin-Kerstien K. G.; Ramakrishnan B.; Qasba P. K.; Hsieh-Wilson L. C. A Chemoenzymatic Approach toward the Rapid and Sensitive Detection of O-GlcNAc Posttranslational Modifications. J. Am. Chem. Soc. 2003, 125 (52), 16162–16163. 10.1021/ja038545r. 14692737

[ref76] Sun Y.; Tang H.; Chen K.; Hu L.; Yao J.; Shaik S.; Chen H. Two-State Reactivity in Low-Valent Iron-Mediated C-H Activation and the Implications for Other First-Row Transition Metals. J. Am. Chem. Soc. 2016, 138 (11), 3715–3730. 10.1021/jacs.5b12150. 26907535

[ref77] De León González F. V.; Boddington M. E.; Kofsky J. M.; Prindl M. I.; Capicciotti C. J. (2024). Glyco-Engineering Cell Surfaces by Exo-Enzymatic Installation of GlcNAz and LacNAz Motifs. ACS Chem. Biol. 2024, 19 (3), 629–640. 10.1021/acschembio.3c00542. 38394345

[ref78] Moremen K. W.; Ramiah A.; Stuart M.; Steel J.; Meng L.; Forouhar F.; Moniz H. A.; Gahlay G.; Gao Z.; Chapla D.; Wang S.; Yang J. Y.; Prabhakar P. K.; Johnson R.; Rosa M. Dela.; Geisler C.; Nairn A. V.; Seetharaman J.; Wu S. C.; Tong L.; Gilbert H. J.; Labaer J.; Jarvis D. L. Expression System for Structural and Functional Studies of Human Glycosylation Enzymes. Nat. Chem. Biol. 2018, 14 (2), 156–162. 10.1038/nchembio.2539. 29251719 PMC5774587

