# Supplementary material for: A Bioorthogonal Precision Tool for Human N-Acetylglucosaminyltransferase V
Source: J Am Chem Soc. 2024 Sep 17;146(39):26707–18. doi: 10.1021/jacs.4c05955 (PMC11450819; doi:10.1021/jacs.4c05955)
Supplement: Supplementary file 1 — ja4c05955_si_001.pdf [file ja4c05955_si_001.pdf]

Supporting Information

A Bioorthogonal Precision Tool for human *N*-acetylglucosaminyltransferase V

Yu Liu[a,b§], Ganka Bineva-Todd[b§], Richard W. Meek[c,d], Laura Mazo[e], Beatriz Piniello[e], Olga Moroz[c], Sean A. Burnap[i,j], Nadima Begum[a], André Ohara[g], Chloe Roustan[f], Sara Tomita[a], Svend Kjaer[f], Karen Polizzi[g], Weston B. Struwe[i,j], Carme Rovira[e,h], Gideon J. Davies[c], Benjamin Schumann[a,b]*

[a] Department of Chemistry, Imperial College London, London W12 0BZ, U.K.

[b] Chemical Glycobiology Laboratory, The Francis Crick Institute, London NW1 1AT, U.K.

[c] York Structural Biology Laboratory, Department of Chemistry, University of York, Heslington, York YO10 5DD, U.K.

[d] School of Biological Sciences, Faculty of Environmental and Life Sciences, University of Southampton, Southampton SO17 1BJ, U.K.

[e] Departament de Química Inorgànica i Orgànica (Secció de Química Orgànica) and Institut de Química Teòrica i Computacional (IQTCUB), Universitat de Barcelona, Martí i Franquès 1, 08028 Barcelona, Spain.

[f] Structural Biology Science Technology Platform, The Francis Crick Institute, London NW1 1AT, U.K.

[g] Department of Chemical Engineering and Imperial College Centre for Synthetic Biology, Imperial College London, London SW7 2AZ, U.K.

[h] Institució Catalana de Recerca i Estudis Avançats (ICREA), Passeig Lluís Companys 23, 08020 Barcelona, Spain.

[i] Department of Biochemistry, Dorothy Crowfoot Hodgkin Building, University of Oxford, South Parks Road, Oxford OX1 3QU, U.K.

[j] The Kavli Institute for Nanoscience Discovery, Dorothy Crowfoot Hodgkin Building, South Parks Road, Oxford OX1 3QU, UK

[§] Contributed equally

* Correspondence: b.schumann@imperial.ac.uk

Supporting Figures

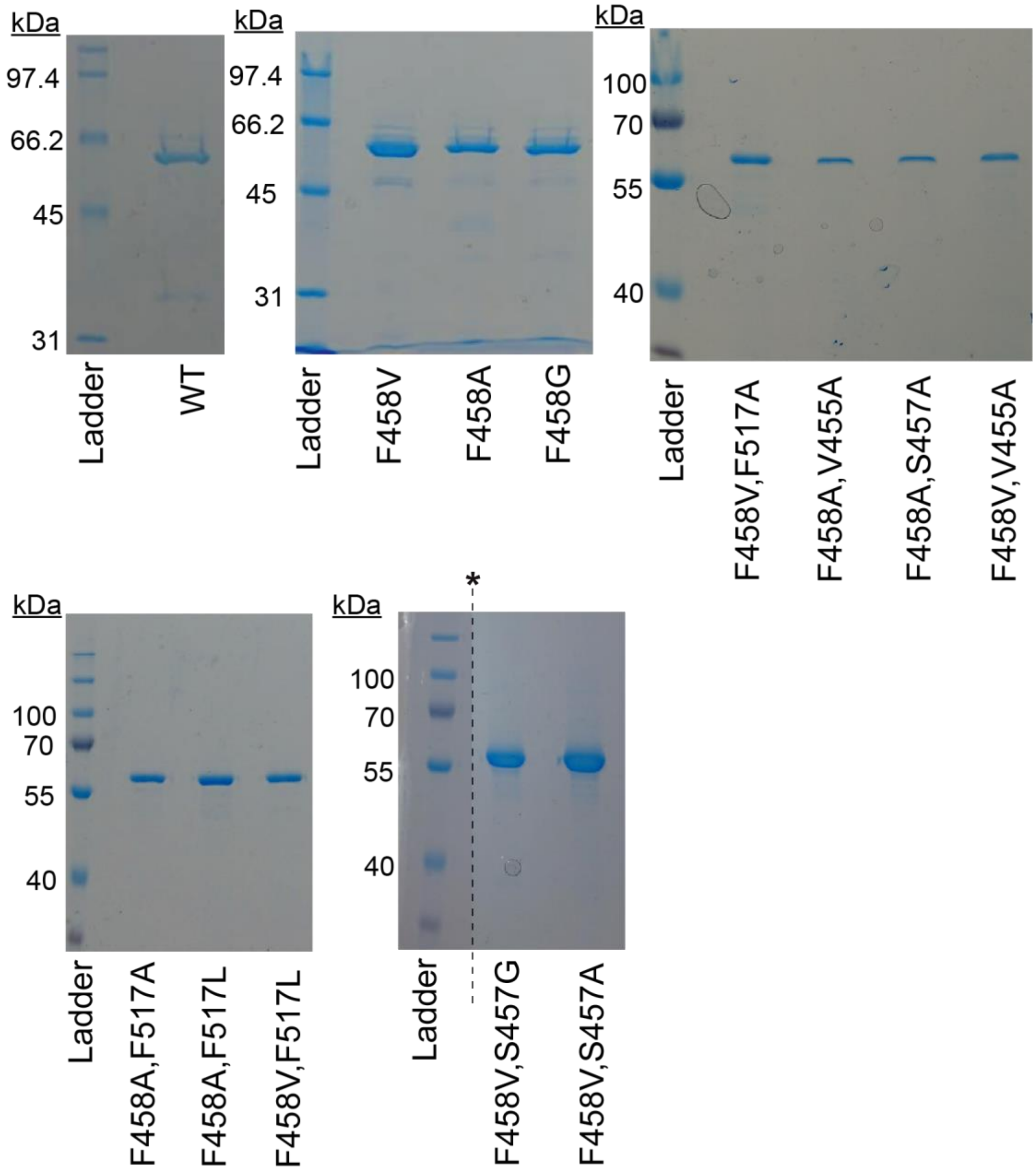

Supporting Fig. 1: Recombinant MGAT5 variants as assessed by SDS-PAGE and Coomassie staining. Asterisk and dotted line indicate position where the gel was spliced to remove lanes with unrelated proteins.

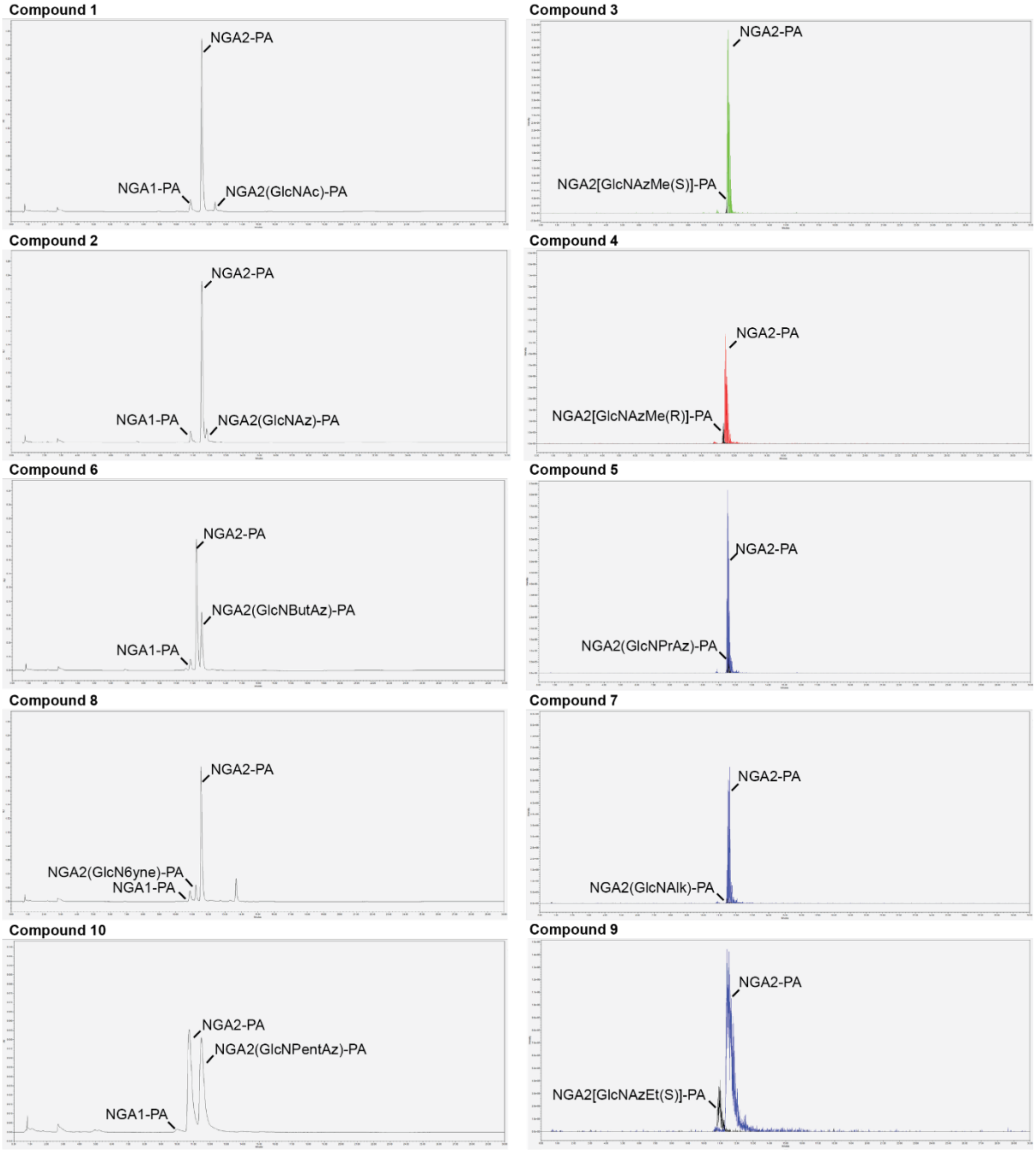

Supporting Fig. 2: Representative traces for incorporation of GlcNAc analogs into of NGA2-PA. Reactions were carried out using 1 mM UDP-GlcNAc or UDP-GlcNAc analogs, 0.2 mM NGA2-PA, and 800 nM BH-MGAT5. Turnover of UDP-GlcNAc **1** and analogs **2, 6, 8,** and **10** into the corresponding product glycan was measured by the near-UV trace (302 nm absorption) of procainamide in the acceptor substrate and the product glycans. Due to co-elution of acceptor substrate and product glycans, the conversion of analogs **3, 4, 5, 7,** and **9** was measured by MS.

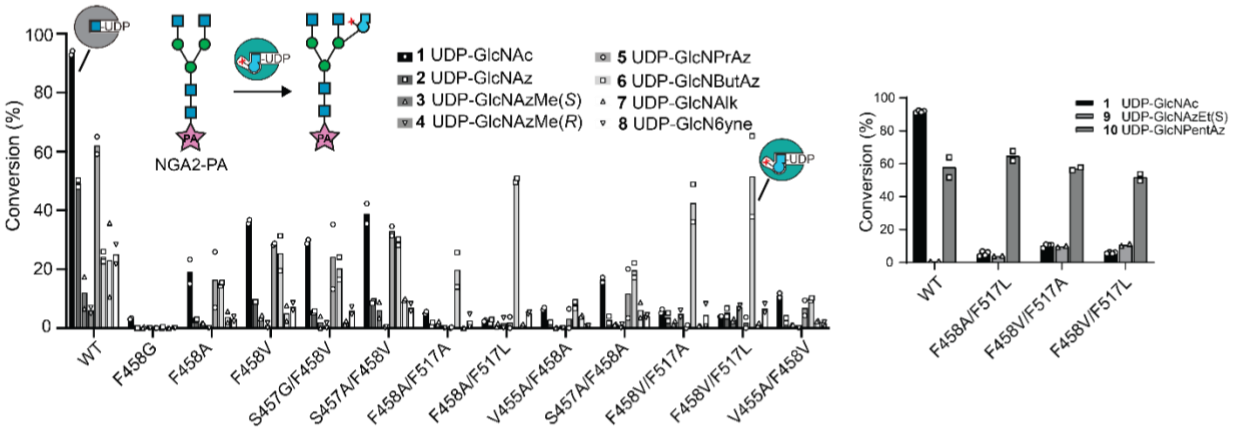

Supporting Fig. 3: MGAT5 variants were subjected to UDP-GlcNAc analogs in endpoint enzymatic assays, using a procainamide (PA)-tagged heptasaccharide as an acceptor substrate and analysis by UPLC with either UV absorption (**1** and analogs **2**, **6**, **8**, **10**) or MS detection (analogs **3**, **4**, **5**, **7**, **9**). Data are individual data points and means from two independent replicates.

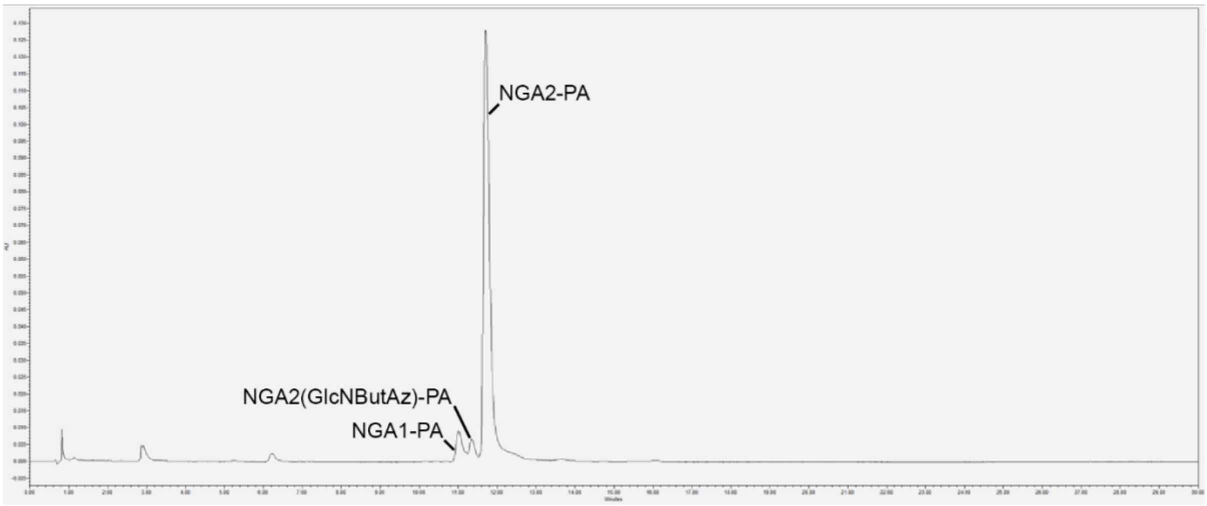

Supporting Fig. 4: HPLC trace for the reaction of 0.2 mM NGA2-PA with 2.5 mM UDP-GlcNButAz **6** using 1 µM WT-MGAT5.

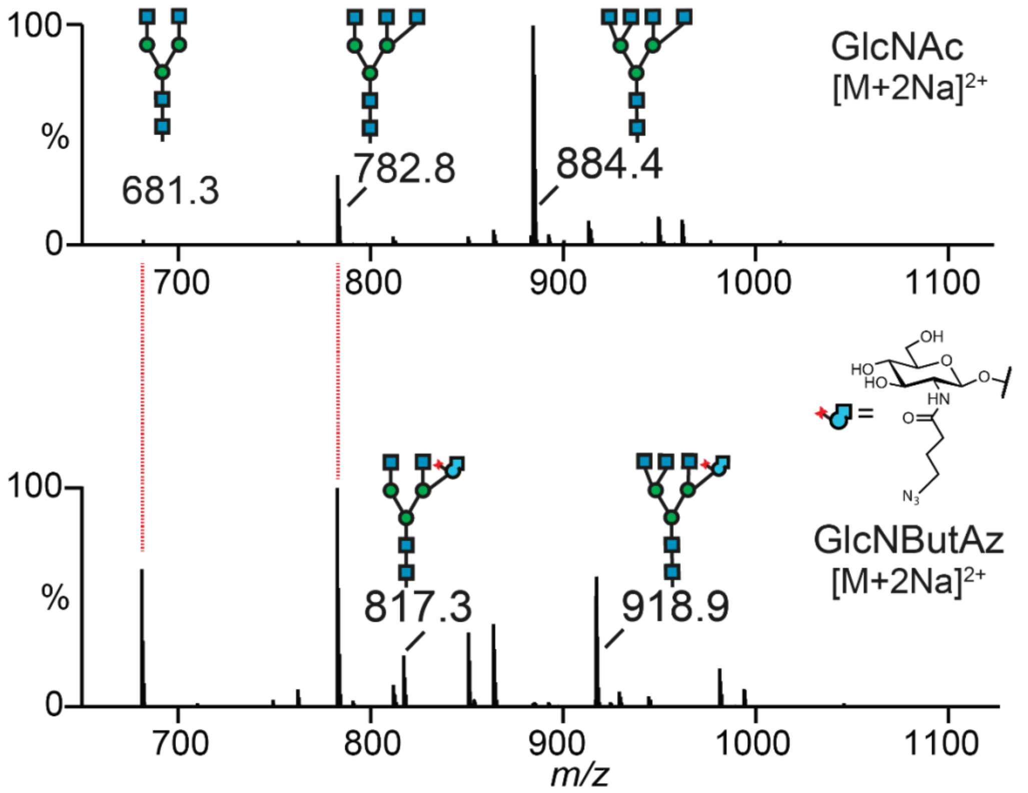

Supporting Fig. 5: MS spectra of released N-glycans showing enzymatic addition of GlcNAc (top) or GlcNButAz (bottom) to asialo-agalactofetuin N-glycans with WT- or BH-MGAT5, respectively.

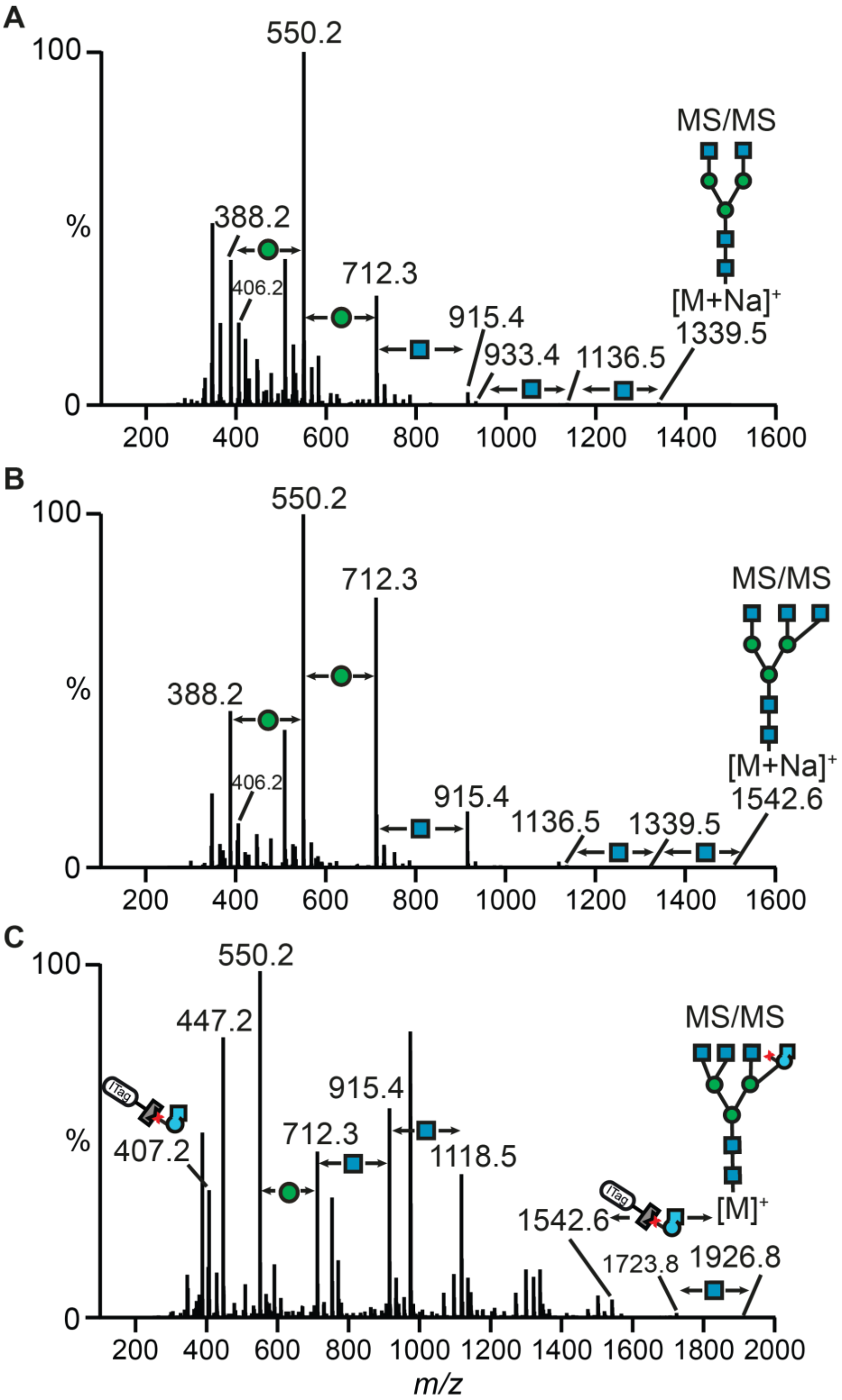

Supporting Fig. 6: MS/MS fragmentation spectra of two unmodified fetuin N-glycans. *A*, $Man_3GlcNAc_4$ (1339.5 *m/z*) and *B*, $Man_3GlcNAc_5$ (1542.6 *m/z*) showing neutral loss of mannose (green circle) and N-acetylglucosamine (blue square). *C*, MS/MS spectrum of modified N-glycan $Man_3GlcNAc_5$GlcNButAz-ITag (1926.8 *m/z*) showing incorporation of GlcNButAz-ITag. The 407.2 *m/z* fragment ion was diagnostic of the GlcNButAz-ITag and was only observed in the modified N-glycan MS/MS spectra (see also Fig.4C).

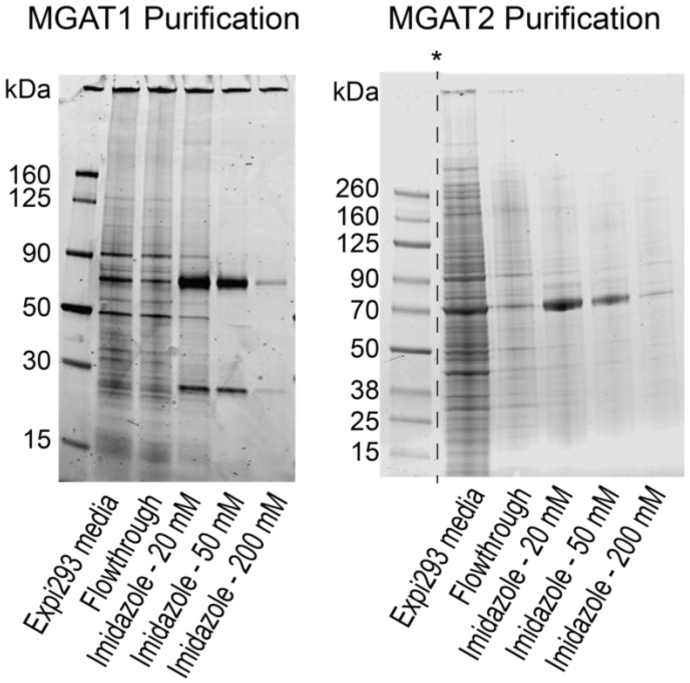

Supporting Fig. 7: Ni-NTA-purified recombinant MGAT1 and recombinant MGAT2 were assessed by SDS-PAGE followed by Coomassie staining. Asterisk and dotted line indicate position where the gel was cropped to remove lanes containing unrelated samples.

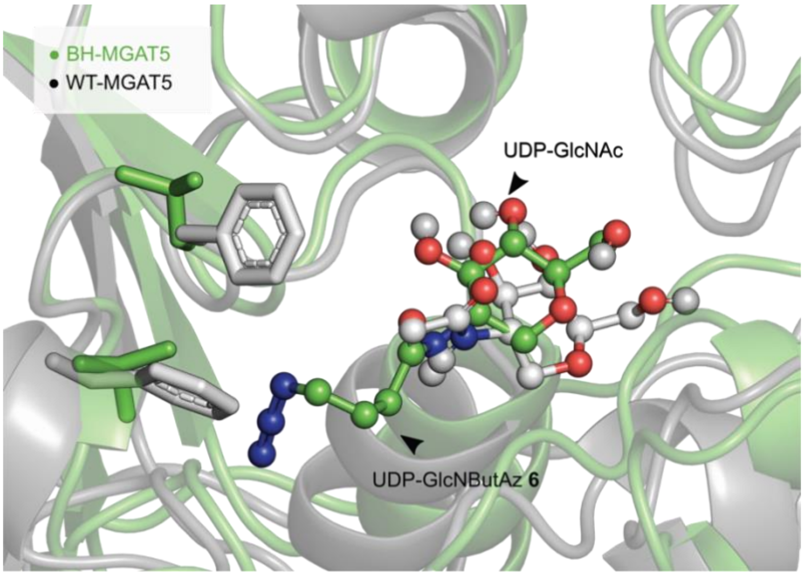

Supporting Fig. 8: Least populated cluster obtained in the MD simulation of BH-MGAT5 in complex with UDP-GlcNButAz **6** and the acceptor M592 (green backbone). The structure of WT-MGAT5 in complex with UDP-GlcNAc and M592 (grey backbone) is shown for comparison.

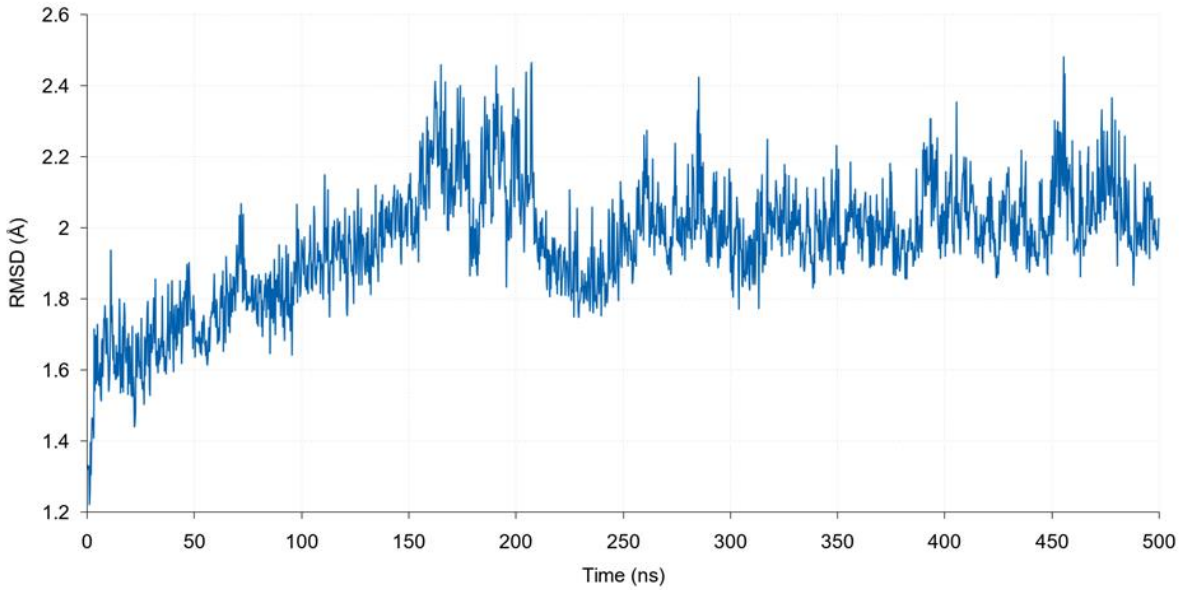

Supporting Fig. 9: Root mean square deviation (RMSD) of backbone atoms during the classical MD simulation.

Experimentals

Table S1: List of key resources.

REAGENT or RESOURCE	SOURCE	IDENTIFIER
**Chemicals, peptides, and recombinant proteins**		
$Ac_4GlcNAz$	Jena Bioscience (Jena, Germany)	Cat# CLK-1085-25
$Ac_4ManNAz$	Jena Bioscience (Jena, Germany)	Cat# CLK-1084-5
Asialofetuin	Sigma-Aldrich (Gillingham, UK)	Sigma-Aldrich Cat# A4781
Benzonase® Nuclease	Merck (Darmstadt, Germany)	Merck Cat# E1014-5KU; CAS No. 9025-65-4
Biotin-alkyne	Biotium (Fremont, CA)	Biotium Cat# 92168
β1-4 Galactosidase	New England Biolabs (Ipswich, USA)	New England Biolabs, Cat# P0745S
BTTAA	Jena Bioscience (Jena, Germany)	Jena Bioscience Cat# CLK-067-25; CAS No. 1334179-85-9
CF680 alkyne	Biotium (Fremont, USA)	Biotium Cat# 96005
Fetuin	Sigma-Aldrich (Gillingham, UK)	Sigma-Aldrich Cat# F3385
Gibco™ MEM α, nucleosides	Gibco	Fisher Scientific UK Ltd Cat#15430584
Gibco™ Penicillin-Streptomycin	Thermo Fisher Scientific	Thermo Fisher Scientific Cat# 15140122
Gibco Express Five ™ SFM media	Thermo Fisher Scientific	Thermo Fisher Scientific Cat# 10486025
Insect-XPRESS™ Protein-free Insect Cell Medium	Lonza	Lonza Cat# BELN12-730Q
L-Glutamine (200 mM)	Thermo Fisher Scientific	Thermo Fisher Scientific Cat# 25030081
FuGENE® 4K Transfection Reagent	Promega	Promega Cat# E5911
Halt™ Protease Inhibitor Cocktail (100X)	Thermo Fisher Scientific	Thermo Fisher Scientific Cat# 1861279
IRDye® 800CW Streptavidin	LI-COR Biosciences	LI-COR Biosciences Cat# 926-32230
Lipofectamine™ LTX Reagent with PLUS™ Reagent	Thermo Fisher Scientific	Thermo Fisher Scientific Cat# 15338100
Nuclease Free Water	Thermo Fisher Scientific	Thermo Fischer Scientific Cat# AM9937
Opti-MEM® I Reduced Serum Medium	Thermo Fisher Scientific	Thermo Fischer Scientific Cat# 31985062
PROC Labelled A2 Glycan	Ludger Ltd (Abingdon, UK)	Cat# CPROC-A2-SPBULK
PROC Labelled MAN5 Glycan	Ludger Ltd (Abingdon, UK)	Cat# CPROC-MAN5-SPBULK
SafeBlue Protein Stain	NBS Biologicals (Huntingdon, UK)	NBS Biologicals Cat# NBS-SB1L

SfiI Restriction Enzyme	New England Biolabs (Ipswich, USA)	New England Biolabs Cat# R0123S
SialEXO Lyophilized	Genovis (Kävlinge Sweden)	Genovis Cat# G1-SM1-020
**Critical commercial assays**		
ExpiFectamine™ 293 Transfection Kit	Thermo Fischer Scientific	Thermo Fischer Scientific Cat# A14525
In-Fusion HD Cloning Kit	Takara Bio	Takara Bio Cat# 102518
NucleoSpin® Gel and PCR Clean-up Kit	Macherey-Nagel (Düren, Germany)	Macherey-Nagel Cat# 740609.50
Pierce™ BCA Protein Assay Kit	Thermo Fischer Scientific	Thermo Fischer Scientific Cat# 23227
QIAprep Spin Miniprep Kit	Qiagen	Qiagen Cat# 27106
Revert™ 700 Total Protein Stain Kit	LI-COR Bioscience	LI-COR Bioscience Cat# 926-11016
Trans-Blot Turbo RTA Midi 0.2 µm Nitrocellulose Transfer Kit	Bio-Rad Laboratories (Hercules, USA)	Bio-Rad Laboratories Cat# 1704271
ZymoPURE™ II Plasmid Maxiprep Kit	Zymo Research (Irvine, USA)	Zymo Research Cat# D4203
**Deposited data**		
Crystal structure BH-MGAT5/M592/UDP-GlcNButAz **6**		PDB: 9F5H
Gel and blot full images		
**Experimental models: Cell lines**		
Expi293F™ cells	Thermo Fischer Scientific	Thermo Fischer Scientific Cat# A14527
CHO Lec1	Prof. Pamela Stanley, Albert Einstein College Medicine	RRID: CVCL_3440
CHO Lec4	Prof. Pamela Stanley, Albert Einstein College Medicine	RRID: CVCL_IP61
High Five™ Cells in Express Five™ Medium	Thermo Fisher Scientific	Thermo Fisher Scientific Cat# B85502
SF9 cells in Sf-900™ III SFM media	Thermo Fisher Scientific	Thermo Fisher Scientific Cat# 12659017
Stellar™ Competent Cells	Takara Bio	Takara Bio Cat# 636763
**Recombinant DNA**		
pGEn2-DEST-hMGAT1	Moremen et al., 2018	DNASU clone# HsCD00413193
pGEn2-DEST-hMGAT2	Moremen et al., 2018	DNASU clone# HsCD00413203
**Software and algorithms**		
CCP4 software package	Winn et al., 2010	https://www.ccp4.ac.uk/
Coot	Emsley et al., 2010	https://www2.mrc-lmb.cam.ac.uk/personal/pemsley/coot/
GraphPad Prism	Dotmatics (Boston, USA)	N/A

H++	Gordon, J. C. et al., 2005	http://biophysics.cs.vt.edu/H++
Illustrator 2023	Adobe (San Jose, USA)	N/A
Image Studio Pro	LI-COR Biosciences (Lincoln, USA)	N/A
The PyMOL Molecular Graphics System	Schrödinger (NewYork, USA).	N/A
SnapGene	Dotmatics	N/A
**Other**		
4–20% Criterion™ TGX™ Precast Midi Protein Gel, 12+2 well, 45 µL	Bio-Rad Laboratories	Bio-Rad Laboratories Cat# 5671093
Amicon Ultra-0.5 Centrifugal Filters (3 kDa MWCO)	Merck Millipore	Merck Cat# UFC500396
HisTrap excel 5ml columns	Cytiva	Cytiva Cat# 17371206
Nalgene™ 0.2 µM PES syringe filter	Thermo Fisher Scientific	Thermo Fisher Scientific Cat# 720-1320
Ni-NTA Agarose	QIAGEN (Manchester, UK)	QIAGEN Cat# 30230
Odyssey CLx	LI-COR Bioscience	LI-COR Bioscience Model # 9140
Subcellular Protein Fractionation Kit for Cultured Cells	Pierce Biotechnology	Thermo Fisher Scientific Cat# 78840

Expression and purification of recombinant AGX1 variants

Recombinant WT-AGX1 and $AGX1^{F383A}$ proteins have been expressed before.[1] The expression constructs for $AGX1^{F381G}$, $AGX1^{F383G}$, $AGX1^{F381A}$, $AGX1^{F381G/F383G}$ and $AGX1^{F381A/F383A}$ were generated as described before,[1] using coding sequences of mutant AGX1 in pIRES-puro plasmids as templates.[2] AGX1 fragments were cloned into BamHI/BglII-digested pTriEX with an N-terminal GST-3c cleavage site and a C-terminal FLAG-tag by using the In-Fusion HD Cloning Kit (Takara, Tokyo, Japan) and primers CCCTAAGCTTGGATCCCATGAACATTAATGACCTCAAACTCACG and GCTCGGTACCAGATCTTCACTTGTCGTCATCGTCTTTGTAGTCAA as described.[1] Plasmids were transfected into Sf21 cells (ATCC CRL-1711) and AGX1 constructs expressed using the Baculovirus system with a transfer plasmid method and the *flash*BAC™ system (Oxford Expression Technologies, Oxford, UK) exactly as described before for WT-AGX1 and $AGX1^{F383A}$.[1] On average, approx. 2 mg recombinant protein was obtained from 2 million cells through this protocol, with no discernible difference in expression levels between AGX1 constructs.

Expression and purification of MGAT1 and MGAT2

A construct encoding the soluble version of MGAT1 comprising N-terminal His_8 tag, N-terminal GFP tag, followed by amino acids 30-445 of human MGAT1 in pGEn2-DEST was from DNASU (HsCD00413193) and originally made by Moremen and colleagues.[3] A construct encoding the soluble version of MGAT2 comprising N-terminal His_8 tag, N-terminal GFP tag, followed by amino acids 30-447 of human MGAT2 in pGEn2-DEST was from DNASU (HsCD00413203) and originally made by Moremen and colleagues.[3] For expression, 50 µg of endotoxin-free maxiprep DNA (ZymoPURE™ II Plasmid Maxiprep Kit, Zymo Research, US) was transfected into 50 mL of 3.5×10^6 cells/mL Expi293F® cells (Gibco) using ExpiFectamine™ 293 Transfection Kit (Thermo Fisher) and following the manufacturer's protocol.

After 4 days incubation at 37 °C, the media was collected by centrifugation at 500 × g for 5 min. The recombinant protein was purified via its His_8 tag by incubating the media with 100 µL of Ni-NTA Agarose beads (QIAGEN) pre-equilibrated in wash buffer A (25 mM Tris-HCl pH 7.5 with 150 mM NaCl, 20 mM imidazole) for 16 h at 4 °C with mixing. Flowthrough was collected by centrifugation at 500 × g for 10 min, beads were washed twice with 1 mL of wash buffer A, twice with 1 mL of washing buffer B (25 mM Tris-HCl pH 7.5 with 150 mM NaCl, 50 mM imidazole), and protein was eluted in 2 × 1 mL of elution buffer (25 mM Tris-HCl pH 7.5 with 150 mM NaCl, 200 mM imidazole). For collection of each wash or eluted fraction, sample was centrifugated at 2,000 × g for 5 min at 4 °C. All fractions were checked by SDS-PAGE (Supporting Fig.7). Protein concentrations were determined by fitting band intensity to a linear curve produced by serially diluted BSA samples that were separated on the same SDS-gel. A concentration of 0.227 mg/mL was determined for MGAT1 and a concentration of 0.264 mg/mL was determined for MGAT2. Enzyme fractions were buffer exchanged to 20 mM HEPES pH 7.5 with 200 mM NaCl, 1 mM DTT and 20% Glycerol, aliquoted, flash-frozen in liquid nitrogen and stored at -80 °C.

Construction of wild-type and mutant MGAT5 expressing baculovirus

Soluble MGAT5 constructs comprising the amino acids 214-741 of human MGAT5 were used for enzyme assays, as described previously.[4] To aid crystallography studies, a triple mutant (Glu297Ala, Phe458Val, and Phe517Leu) with a truncation between Lys329 and Ile345 was generated.[4] An adapted QuikChange site-directed mutagenesis protocol was used to introduce mutations into previously described MGAT5 constructs within pOMNIBac plasmids.[4] Mutations were verified by Sanger sequencing. Recombinant bacmids were produced using a Tn7 transposition protocol in DH10EMBacY cells (Geneva Biotech,

Pregny-Chambésy, Switzerland).[5,6] Bacmids were purified using the PureLink HiPure Plasmid Miniprep kit (Invitogen, Waltham, USA) using manufacturer's instructions. V1 baculovirus stocks were generated by statically incubating 6 × 2 mL of 0.45 × 10^6 cells/mL SF9 cells with 180 µL of transfection master mix (1,050 µL Insect-XPRESS media (Lonza, Basel, Switzerland), 38 µL of bacmid at 40-150 ng/µL, and 31.5 µL FuGENE HD transfection reagent (Promega, Madison, USA)) at 28 °C until cells were 95% fluorescent (approximately 2 days). V1 baculovirus stocks were clarified from cells and debris by centrifugation at 200 × g for 5 min and supplemented with fetal bovine serum to a final concentration of 2%. To generate V2 baculovirus stocks, 50 mL of SF9 cells at 1 × 10^6 grown in Insect-XPRESS media (Lonza) were incubated with 1 mL of V1 stock at 28 °C, shaking, until cells were 95% fluorescent. V2 stocks were clarified by centrifugation for 5 min at 200 × g, supplemented with 2% fetal bovine serum, and stored at 4 °C until use.

Protein expression and purification of wildtype and mutant variants of MGAT5

Purification procedures are adapted from protocols previously described.[4] High Five cells (*Trichoplusia ni*) at ~2 x 10^6 grown in Gibco Express Five SFM media supplemented with 18 mM L-Glutamine were transfected with 1 mL of V2 baculovirus stock per 600 mL culture. For kinetics analyses, 600 mL of cells were transfected for each construct, for crystallography 3.9 L of cells were transfected. At ~72 hours post-transfection cells were examined for viability (>90%) and fluorescence. Conditioned media was clarified from cells and debris by a 2-step 4 °C centrifugation, the first step at 200 × g for 15 min and a second step at 4,000 × g for 60 min (media transferred to clean centrifuge bottles between steps). Clarified conditioned media was supplemented with 1 mM DTT and 0.2 mM AEBSF and loaded onto a 5 mL HisTrap Excel Column (Cytiva, Marlborough, USA) pre-equilibrated in Buffer A (20 mM Tris pH 8.0, 500 mM NaCl, 20 mM Imidazole, 1 mM DTT). Column was washed with 5 column volumes (CV) of buffer A before protein was eluted with a linear gradient of 0-100% Buffer B (20 mM Tris pH 8.0, 500 mM NaCl, 1 M Imidazole, 1 mM DTT) in Buffer A over 20 CV.

Fractions containing MGAT5 protein were pooled and diluted 10x with Buffer C (20 mM MES pH 6.0, 1 mM DTT) and loaded onto a 1 mL HiTrap SP HP column (Cytiva) pre-equilibrated in Buffer C for ion-exchange (IEX) chromatography. The column was washed with 10 CV of Buffer C, before protein was eluted with a linear gradient of 0-100% Buffer D (20 mM MES pH 6.0, 1 M NaCl, 1 mM DTT) in Buffer C over 20 CV. Fractions containing MGAT5 protein were pooled, corrected to pH 8.0 with 1M Tris-HCl pH 8.0 and incubated overnight with 6xHis-3C protease at a mass ratio of 1:100 at ambient temperature. This step removed the

melittin signal sequence and His-tag. For MGAT5 constructs F458V/S457G and F458V/S457A, a low A280 absorbance indicated considerably less protein expressed compared to other constructs, subsequently protein concentrations were too low for the IEX chromatography step. These constructs were instead diluted 1:20 with 20 mM Tris-HCl pH 8.0, 500 mM NaCl and 1 mM DTT and incubated with 6xHis-3C protease as above.

Digest reactions were passed over a 1 mL HisTrap HP column (Cytiva) pre-equilibrated in Buffer A followed by 20 CV of Buffer A. Flowthrough was collected and concentrated to <2 mL with a 10 KDa MWCO Vivaspin centrifugal concentrator (Sartorius, Göttingen, Germany). Samples destined for kinetic analyses were diluted 20x in Buffer E (20 mM HEPES pH 7.4, 200 mM NaCl, 1 mM DTT) and concentrated to < 2 mL. This step was repeated twice. For samples destined for crystallography, the initial concentrated protein was size excluded on a Superdex S75 16/600 column (Cytiva) pre-equilibrated in Buffer E. Samples containing MGAT5 protein were pooled and concentrated as before. All protein samples were quantified by A_{280} absorbance, aliquoted, flash frozen in liquid nitrogen, and stored at -80 °C until use. Protein purity was confirmed by SDS-PAGE (Supporting Fig.1)

In vitro glycosylation assay using synthetic glycan acceptor substrate

Commercial procainamide-labelled A2 glycan (NGA2-PA or PROC-A2; Ludger Ltd, Abingdon, UK) was used as an acceptor substrate to assess conversion by a near UV (302 nm) absorbance-based assay. Reactions were carried out using 1 mM UDP-GlcNAc or UDP-GlcNAc analogs, 0.2 mM NGA2-PA, and 800 nM MGAT5 constructs, in a total volume of 5 µL Reaction Buffer V (50 mM MES pH 6.25, 25 mM NaCl, 0.1 % v/v Triton X-100, and 0.1 % w/v BSA) at 37 °C for 16 h. For competition experiments, reactions were performed using 0.1 mM NGA2-PA, 0.2 mM UDP-GlcNAc analog and either 0.2 mM, 0.4 mM or 0.8 mM UDP-GlcNAc, in a total volume of 10 µL Reaction Buffer V at 37 °C for 16 h in the presence of 800 nM recombinantly expressed WT MGAT5 or BH MGAT5. Reactions were stopped by addition of an equal volume of acetonitrile, followed by centrifugation at 13,000 × g, 4 °C for 30 min. Of each sample, 9 µL of the supernatants were injected onto an Acquity H-Class PLUS QDa UPLC-MS (Waters, Milford, USA) equipped with an ACQUITY UPLC® Glycan BEH Amide column (130 Å, 1.7 µm, 2.1 x 100 mm, Waters). Samples were run at flow rate of 0.35 mL/min using Buffer A (10 mM Ammonium formate at pH 4.5); Buffer B (10 mM Ammonium formate in ACN: water 90:10 (v/v)) with a gradient of 90-55% buffer B over 17 min. The percentage of turnover of procainamide-labelled substrate glycan into the corresponding product glycan was calculated by integration of the near-UV trace (302 nm absorption) of the acceptor substrate and the product glycan or by integration of the MS

peaks in the scenario where acceptor substrate and the product glycan co-eluted, and determined as Turnover % = Peak Area of Product Glycan / Peak Area of (Substrate Glycan + Product Glycan) %.

Enzyme kinetics

Recombinant BH-MGAT5 at a concentration of 1 µM was incubated for 1 h with serially diluted UDP-GlcNButAz **6** from 0.04 to 2.5 mM and 0.2 mM NGA2-PA in Reaction Buffer V in a total volume of 5 µL, and turnover measured by UPLC as described above. To quantify the formation of product, standard curves were employed by plotting concentrations of serially diluted NGA2-PA against the integrated peak area under the near-UV trace (302 nm absorption). The kinetics curve was plotted with Prism 10 (GraphPad, San Diego, USA) and fitted with a Michaelis Menten function to calculate k_{cat}, K_M and V_{max}.

Bioorthogonal labelling of asialo-agalactofetuin by BH-MGAT5

Asialofetuin (Sigma-Aldrich, Gillingham, UK) was dissolved in PBS to a concentration of 2 mg/mL. Degalactosylation of 10 µg asialofetuin was performed in the presence of 1.25 U/µL β1-4 galactosidase (New England Biolabs, Ipswich, USA), 5 mM $CaCl_2$ and 50 mM MES 6.25 in 50 µL reaction volume and incubated for 16 h at 37 °C. Reaction was heat-inactivated at 95 °C for 20 s and cooled to 4 °C.

In vitro glycosylation reactions were performed using 1 µg agalactofetiun fraction in 25 µL reaction volume containing 50 mM MES pH 6.25, 25 mM NaCl, 0.2 mM UDP-GlcNAc analog and either 200 or 800 µM UDP-GlcNAc at 37 °C for 12 h in the presence of 1 µM recombinantly expressed WT-MGAT5 or BH-MGAT5. Reactions were heat-inactivated at 95 °C for 20 s and cooled to 4 °C. Then, azide-containing reaction mixtures were sequentially treated with one-third volume of 400 µM biotin-alkyne (Biotium, Fremont, USA), 4800 µM BTTAA (Click Chemistry Tools, Scottsdale, USA), 1200 µM $CuSO_4$, 20 mM aminoguanidinium chloride and 20 mM sodium ascorbate (final concentrations 100 µM biotin probe, 1200 µM BTTAA, 300 µM $CuSO_4$, 5 mM aminoguanidinium chloride and 5 mM sodium ascorbate). Reaction mixtures were then subjected to SDS-PAGE and blotted on nitrocellulose membranes. The total protein amount was assessed using the REVERT protein staining kit (LI-COR Biosciences, Lincoln, USA), and biotinylation was detected using IRDye 800CW Streptavidin (LI-COR Biosciences) according to the manufacturer's instructions.

For preparation of glycomic analysis samples, 1 mg fetuin (Sigma-Aldrich, Gillingham, UK) was dissolved in PBS to a concentration of 50 mg/mL. Desialylation was performed in the presence of 20 U/µL SialEXO (Genovis, Kävlinge, Sweden) at 37 °C for 16 h. Degalactosylation was performed using 800 µg desialylated fetuin in 80 µL reaction volume containing 2 U/µL β1-4 galactosidase (New England Biolabs, Ipswich, USA), 5 mM $CaCl_2$ and 50 mM MES pH 6.25, and incubated for 16 h at 37 °C. *In vitro* glycosylation reactions were performed using 200 µg of newly prepared asialo-agalactofetiun fraction in 25 µL reaction volume containing 50 mM MES pH 6.25, 25 mM NaCl, 1.5 mM UDP-GlcNButAz in the presence of 500 nM recombinant BH-MGAT5 or 1.5 mM UDP-GlcNAc in the presence of 500 nM recombinant WT-MGAT5 at 37 °C for 12 h. Reactions were heat-inactivated at 95 °C for 10 s and cooled to 4 °C. Unreacted azide was removed by placing the reaction mixture in Amicon Ultra-0.5 Centrifugal Filter (3 kDa cut-off; Merck Millipore) and rinsing twice with 10x volume of PBS. Then, azide-containing reaction mixtures were sequentially treated with one-third volume of 400 µM ITag-alkyne, 4800 µM BTTAA (Click Chemistry Tools, Scottsdale, USA), 1200 µM $CuSO_4$, 20 mM aminoguanidinium chloride and 20 mM sodium ascorbate (final concentrations 100 µM ITag-alkyne, 1200 µM BTTAA, 300 µM $CuSO_4$, 5 mM aminoguanidinium chloride and 5 mM sodium ascorbate). Unreacted click reagents were removed by placing the reaction mixture in Amicon Ultra-0.5 Centrifugal Filter (3 kDa cut-off; Merck Millipore) and rinsing twice with 10x volume of PBS.

Glycan release and ion mobility (IM)–MS

N-glycans were released from 50 µg of protein with 2 µg PNGaseF (produced in-house) at 37 °C for 16 hours. Released N-glycans were desalted using a 0.6 µL C18 ZipTip (Millepore) overlaid with porous graphitized carbon resin (HyperSep™ Hypercarb™ ThermoFisher Scientific). De-salted N-glycans were lyophilized and resuspended in 50% methanol immediately prior to IM-MS analysis. IM-MS measurements were performed on a SELECT SERIES Cyclic IMS instrument (Waters, Manchester, UK). For each sample analysis, 2 µL of N-glycan sample material was ionized by nano-electrospray ionization (nano-ESI) from gold-coated borosilicate glass capillaries prepared in-house[25]. Data was acquired in positive ionisation mode with the settings as follows: capillary voltage 1.4 kV, cone voltage 160 V, source offset voltage 10 V, source temperature 100 °C. A single-pass cyclic control ion mobility method was used, with an injection time of 10 ms, separation time 5 ms and eject and acquire time 34 ms. Collision-induced dissociation was performed in the trap with argon as the collision gas with a collision energy (CE) between 80-120 V. Data acquisition and processing were carried out using the Waters MassLynx™ (version 4.2).

Bioorthogonal tagging of a cellular asialo-agalactoglycoprotein fraction by BH-MGAT5

The Lec4 Chinese hamster ovary (CHO) cell line was a kind gift from Prof. Pamela Stanley (Albert Einstein College of Medicine, New York, USA).[7,8] One 75-cm^2 flask of cells were grown to confluency in MEMα medium (Fisher Scientific UK Ltd, Loughborough, UK) containing 10% (v/v) fetal bovine serum, 100 U/mL penicillin-streptomycin (Thermo Fisher Scientific). Cells were rinsed three times with phosphate-buffered saline, harvested at 500 × g for 5 min. Pelleted cells were resuspended in ice-cold PBS and transferred to a 1.5 mL microcentrifuge tube, centrifuged at 500 × g, 4 °C, for 3 min and supernatant removed. The Subcellular Protein Fractionation Kit for Cultured Cells (Thermo Fisher Scientific) was followed to obtain a membrane fraction. Protein concentration was determined using the Rapid Gold BCA Protein Assay Kit (Thermo Fisher Scientific). The membrane fraction was concentrated to 12 mg/mL using an Amicon Ultra-0.5 centrifugal filter (Merck Millipore). Desialylation was performed by incubating 600 µg of Lec4 membrane fraction with 10 U SialEXO (dissolved in PBS; Genovis) in 50 µL membrane fraction for 16 h at 37 °C. The reaction was heat-inactivated at 95 °C for 20 s and cooled to 4 °C. Degalactosylation of all the desialylated Lec4 membrane fraction from the previous step was performed in the presence of 0.4 U/µL β1-4 galactosidase (New England Biolabs, Ipswich, USA), 5 mM $CaCl_2$ and 50 mM MES pH 6.25 in 50 µL reaction volume, and incubated for 16 h at 37 °C. The reaction was heat-inactivated at 95 °C for 20 s and cooled to 4 °C.

In vitro glycosylation reactions were performed using 20 µg asialo-agalacto-Lec4 membrane fraction in 10 µL reaction volume containing 50 mM MES pH 6.25, 25 mM NaCl, 200 µM UDP-GlcNButAz **6** and either 200 µM or 800 µM UDP-GlcNAc **1** at 37 °C for 12 h in the presence of 500 nM recombinantly expressed WT-MGAT5 or BH-MGAT5. Reactions were heat-inactivated at 95 °C for 20 s and cooled to 4 °C. Then, azide-containing reaction mixtures were sequentially treated with one-third volume of 400 µM biotin-alkyne (Biotium, Fremont, USA), 4800 µM BTTAA (Click Chemistry Tools, Scottsdale, USA), 1200 µM $CuSO_4$, 20 mM aminoguanidinium chloride and 20 mM sodium ascorbate (final concentrations 100 µM biotin probe, 1200 µM BTTAA, 300 µM $CuSO_4$, 5 mM aminoguanidinium chloride and 5 mM sodium ascorbate). Reaction mixtures were then subjected to SDS-PAGE and blotted on nitrocellulose membranes. The total protein amount was assessed using the REVERT protein staining kit (LI-COR Biosciences, Lincoln, USA), and biotinylation was detected using IRDye 800CW Streptavidin (LI-COR Biosciences) according to the manufacturer's instructions.

In vitro N-glycan elaboration of a Man5-containing model glycoprotein by WT-MGAT1, MAN2A1, and WT-MGAT2, followed by bioorthogonal tagging by BH-MGAT5

A construct of monomeric antibody Fc containing a single Man5 N-glycan per polypeptide was expressed in the *P. pastoris* SuperMan5 (Biogrammatics) as described previously.[9]

In vitro N-glycan elaboration was performed using 30 µg Fc-Man5 in 60 µL reaction volume containing 200 mM MES pH 6.25, 25 mM NaCl, 5 mM $MnCl_2$, 1 mM $ZnCl_2$, 1 mM UDP-GlcNAc at 37 °C for 16 h in the presence of 500 nM WT-MGAT1, 150 nM drosophila MAN2A1, and 250 nM WT-MGAT2. Reactions were heat-inactivated at 95 °C for 20 s and cooled to 4 °C.

In vitro glycosylation reactions were performed using 1 µg N-glycan-elaborated Fc protein in 10 µL reaction volume containing 50 mM MES pH 6.25, 25 mM NaCl, 200 µM UDP-GlcNAc analog and either 200 µM or 800 µM UDP-GlcNAc at 37 °C for 12 h in the presence of 500 nM recombinantly expressed WT-MGAT5 or BH-MGAT5. Reactions were heat-inactivated at 95 °C for 20 s and cooled to 4 °C. Then, azide-containing reaction mixtures were sequentially treated with one-third volume of 400 µM biotin-alkyne (Biotium, Fremont, USA), 4800 µM BTTAA (Click Chemistry Tools, Scottsdale, USA), 1200 µM $CuSO_4$, 20 mM aminoguanidinium chloride and 20 mM sodium ascorbate (final concentrations 100 µM biotin probe, 1200 µM BTTAA, 300 µM $CuSO_4$, 5 mM aminoguanidinium chloride and 5 mM sodium ascorbate). Reaction mixtures were then subjected to SDS-PAGE and blotted on nitrocellulose membranes. The total protein amount was assessed using the REVERT protein staining kit (LI-COR Biosciences, Lincoln, USA), and biotinylation was detected using IRDye 800CW Streptavidin (LI-COR Biosciences) according to the manufacturer's instructions.

In vitro N-glycan elaboration of Man5-containing cellular fraction by WT-MGAT1, MAN2A1, and WT-MGAT2, followed by bioorthogonal tagging by BH-MGAT5

The Lec1 Chinese hamster ovary (CHO) cell line was a kind gift from Prof. Pamela Stanley (Albert Einstein College of Medicine, New York, USA).[7,8,10] One 75-cm^2 flask of cells were grown to confluency in MEMα medium (Fisher Scientific UK Ltd, Loughborough, UK) containing 10% (v/v) fetal bovine serum, 100 U/mL penicillin-streptomycin (Thermo Fisher Scientific). Cells were rinsed three times with phosphate-buffered saline, harvested at 500 × g for 5 min. Pelleted cells were resuspended in ice-cold PBS and transferred to a 1.5 mL microcentrifuge tube, centrifuged at 500 × g, 4 °C, for 3 min and supernatant removed. The Subcellular Protein Fractionation Kit for Cultured Cells (Thermo Fisher Scientific) was

followed to obtain the membrane fraction. Protein concentration was determined using the Rapid Gold BCA Protein Assay Kit (Thermo Fisher Scientific). The membrane fraction was concentrated to 16 mg/mL using an Amicon Ultra-0.5 centrifugal filter (Merck Millipore).

In vitro N-glycan elaboration was performed using 30 µg Lec1 membrane fraction in 60 µL reaction volume containing 200 mM MES pH 6.25, 25 mM NaCl, 5 mM $MnCl_2$, 1 mM $ZnCl_2$, 1 mM UDP-GlcNAc at 37 °C for 16 h in the presence of 500 nM WT-MGAT1, 150 nM drosophila MAN2A1, and 250 nM WT-MGAT2. Reactions were heat-inactivated at 95 °C for 20 s and cooled to 4 °C.

In vitro glycosylation reactions were performed using 1 µg N-glycan-elaborated Lec1 membrane fraction in 10 µL reaction volume containing 50 mM MES pH 6.25, 25 mM NaCl, 200 µM UDP-GlcNButAz **6** and either 200 µM or 800 µM UDP-GlcNAc **1** at 37 °C for 12 h in the presence of 500 nM recombinantly expressed WT-MGAT5 or BH-MGAT5. Reactions were heat-inactivated at 95 °C for 20 s and cooled to 4 °C. Then, azide-containing reaction mixtures were sequentially treated with one-third volume of 400 µM biotin-alkyne (Biotium, Fremont, USA), 4800 µM BTTAA (Click Chemistry Tools, Scottsdale, USA), 1200 µM $CuSO_4$, 20 mM aminoguanidinium chloride and 20 mM sodium ascorbate (final concentrations 100 µM biotin probe, 1200 µM BTTAA, 300 µM $CuSO_4$, 5 mM aminoguanidinium chloride and 5 mM sodium ascorbate). Reaction mixtures were then subjected to SDS-PAGE and blotted on nitrocellulose membranes. The total protein amount was assessed using the REVERT protein staining kit (LI-COR Biosciences, Lincoln, USA), and biotinylation was detected using IRDye 800CW Streptavidin (LI-COR Biosciences) according to the manufacturer's instructions.

Crystallization and structure determination of BH-MGAT5

The initial crystallization of BH-MGAT5 was set up as optimizations based on the conditions reported,[4] namely 0.1 M HEPES pH 8.0, 0.3 M Li_2SO_4, 30 % (w/v) PEG 3350, 10 % (v/v) ethylene glycol, with 150 nL protein solution plus 150 nL reservoir solution in 96-well format plates (MRC 2-well crystallization microplate, Swissci, Switzerland) equilibrated against 54 µL reservoir solution**.** After the initial hits had been obtained, seeding stock was prepared and microseed matrix screening (MMS) was carried out in similar optimizations as well as several commercial screens. Briefly, crystals were transferred onto a glass slide, crushed and collected in a Seed Bead (Hampton Research) with 30 µL well solution added, vortexed for 1 min and used as an initial seeding stock. Unused seeding stocks were stored at -20° C for later experiments. For MMS experiments, the finals drops contained 150 nL of protein solution (or protein-ligand complex), with 50 nL seeding stock and 100 nL reservoir solution

in an upper drop of the MRC 2-well plate, all simultaneously dispensed by a three-bore microtip of Oryx robot (Douglas instruments), and 150 nL sample /150 nL reservoir for the lower drop, allowing carry-over seeds to act instead of a highly diluted seeding stock, which is a useful feature of the Oryx robot that was used for all crystallization experiments.

Initially, crystallizations were set up for the apoprotein, with soaking in UDP-GlcNButAz **6** for different time lengths before fishing the crystals for data collection. Several data sets were collected but the ligand was not bound. Further experiments were changed to co-crystallization of pre-mixed complexes of MGAT5 triple mutant with UDP-GlcNButAz **6** (10 mM), UDP-GlcNButAz **6** (10 mM) and M592 (1-10 mM), and UDP (10 mM) + M592 (1 mM). Final crystallization conditions for the complexes were in the range of 15-20% PEG3350, 0.1 M Tris-HCl pH 8-8.5, 0.3 M Li_2SO_4, 0-10% ethylene glycol.

Data were collected at the Diamond Light Source and structures were determined for all potential complexes using unliganded MGAT5 (PDB entry 6YJR) as the molecular replacement model, however, no bound ligand could be seen for UDP-GlcNButAz **6** only complex, while not very well ordered M592 was present in UDP-GlcNButAz **6**/M592 complex. Both UDP and M592 were present in the corresponding complex structure, and bump-and-hole mutations were visible - this structure has been refined and deposited to PDB.

All computations were carried out using programs from the CCP4 suite,[11] unless otherwise stated. Data collection and processing and final refinement statistics are given in Table 1**.** Data were processed using DIALS within the Xia2 pipeline and scaled with Aimless.[12–14] The structure was refined with REFMAC alternating with interactive model correction in COOT.[15,16] The quality of the final model was validated using Molprobity.[17] Structural parameters are indicated in Table S2.

Table S2: Structure solution and refinement. Values for the outer shell are given in parentheses.

Beamline	I03
Wavelength (Å)	0.727
Date	15/09/2023
Space group	P1
Unit cell	a=46.51 (Å), b=69.07(Å), c=90.99(Å), α=108.21°, β=92.09°, γ =106.75°
Resolution range (Å)	44.10 - 1.97 (2.01 – 1.97)
Total reflections	258321 (16891)
Unique reflections	68392 (4432)
Wilson B-factor	31.36
Completeness (%)	94.4 (90.8)
Multiplicity	3.8 (3.8)
R_{meas}	0.17 (2.20)
R_{pim}	0.09 (1.11)
$<I / \sigma(I)>$	6.6 (0.6)
$CC_{1/2}$	0.994 (0.270)
Reflections in refinement	68300
Reflections in free set	3266
R_{work}	0.2000
R_{free}	0.2420
RMSD bonds	0.0119
RMSD angles	2.139
Ramachandran favored (%)	96.9
Ramachandran allowed (%)	3.1
Ramachandran outliers (%)	0.0
Rotamer outliers	3.5
Clash score	4.1
MolProbity score	1.79
PDB ID	9F5H

Molecular dynamics simulations of BH-MGAT5

The model used for the simulations was based on the triple mutant (Glu297Ala, Phe458Val, and Phe517Leu) X-ray structure. The Glu297Ala mutation was reversed, as it was meant for trapping the ternary complex and is not necessary for bump-and-hole engineering. To the pre-existing UDP structure, GlcNButAz was added by placement following a superposition with the structures from Darby *et al.* with PyMOL (Schrödinger, NewYork, USA).[4] Moreover, the partially resolved acceptor M592 was completed to better compare with the previous results in which M592 was shortened to four sugar moieties due to a lack of resolution on the terminal sugar. Other missing parts such as missing loops and side chains were modeled with Modeller,[18] included in the program Chimera (UCSF).

The protonation states of the charged residues were predicted according to the environment by the software H++,[19] using a pH of 7.00. The system was solvated using a cubic solvation box and adding 29373 water molecules, and 6 Cl^- ions for neutralization. The resulting simulation cell dimensions were 105.18 × 104.73 × 105.18 $Å^3$ (x × y × z). The force field used to model the protein and water molecules is FF14SB and TIP3P, respectively.[20] The GlcNButAz was parametrized using GAFF2 force field,[21] UDP with FF14SB, and the acceptor with GLYCAM06.[22] The molecular dynamics simulations were run using Amber20.[23]

Before the molecular dynamics simulation, energy minimization was performed with 4000 steps (2000 with steepest descent minimization followed by 2000 steps of conjugate gradient minimization). The molecular dynamics simulation then started by heating the system until 298.0 K with constant volume (in the NVT ensemble). The next step was equilibrating the density of the system by letting the volume change and restricting the pressure around 1 atm and temperature (NPT ensemble). Finally, the simulation was extended 0.5 μs more (Supporting Fig.9). The timestep of the simulation was 0.002 ps, and the SHAKE algorithm was used.

Due to the fact that the acceptor is highly exposed to the solvent, low restraints (force constant of 5.0 kcal/(mol·$Å^2$)) had to be introduced on three distances in order to hold the oligosaccharide in place: C1—O6 (donor – acceptor), H6O—OE1 (acceptor – catalytic base), and H1—OE1 (donor – base).

The clustering k-means analysis was performed with the cpptraj utility of AmberTools.[23]

Chemical synthesis

Solvents and reagents were of synthesis and HPLC grade from Sigma Aldrich unless specified otherwise. Anhydrous conditions under inert gas were used unless specified otherwise. Thin layer chromatography was performed on DC-Fertigfolie Polygram SIL G/UV254 pre-coated with silica polyester sheets (0.2 mm thickness) (Macherey-Nagel, Germany). Spots were developed with sugar stain (0.1% (v/v) 3-methoxyphenol, 2.5% (v/v) sulfuric acid in EtOH) dipping solution. Solvents were removed under reduced pressure using a rotary evaporator and high vacuum. Medium pressure chromatography was performed on an Isolera system (Biotage, Uppsala, Sweden).

^{1}H, ^{13}C and ^{31}P NMR spectra were measured with Bruker Avance-400, 600 or 700 MHz spectrometers at 298 K. Chemical shifts (σ) are reported in parts per million (ppm) relative to the respective residual solvent peaks ($CDCl_3$: σ 7.26 in ^{1}H and 77.16 in ^{13}C NMR; CD_3OH: σ 3.31 in ^{1}H and 49.00 in ^{13}C NMR; D_2O: σ 4.79 in ^{1}H). The following abbreviations are used to indicate peak multiplicities: s singlet; d doublet; dd doublet of doublets; dt doublet of triplets; td triple of doublets; m multiplet. Coupling constants (*J*) are reported in Hertz (Hz). NMR data was processed with MestReNova 14.2.2 developed by Mestrelab Research S.L. Low resolution mass spectrometry by electrospray ionization (ESI-LRMS) was performed on an Acquity UPLC-MS (Waters) equipped with either ACQUITY UPLC® BEH C18 or ACQUITY UPLC® Glycan BEH Amide column.

Compounds **3** and **5** were made previously.[2] Compounds **1** and **2** (UDP-GlcNAc and UDP-GlcNAz) are commercially available (Sigma-Aldrich, Chemily Glycoscience).

Compound characterization

2-[(*R*)-2-Azidopropionamido]-2-deoxy-D-glucopyranose (4a)

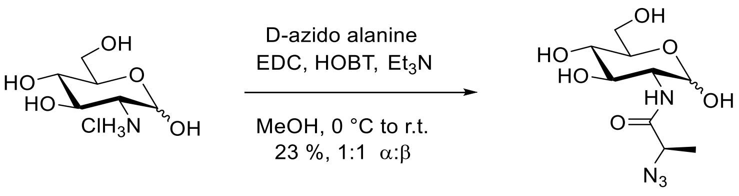

4a

To a stirred solution of D-azido alanine[24] (147 mg, 1.1 mmol, 2.4 eq.) in anhydrous methanol (5 mL) were added D-glucosamine hydrochloride (100 mg, 0.47 mmol, 1 eq.) and Et_3N (175 µL, 1.25 mmol, 2.67 eq.). The solution was cooled to 0 °C and EDC (195 mg, 1.25 mmol, 2.67 eq.) and HOBT (76 mg, 0.56 mmol, 1.2 eq.) were added. The reaction was warmed to

r.t. and stirred for 16 h. On completion, the solvent was evaporated, and the crude mixture purified on medium pressure flash chromatography (Sfär Silica D Duo 25 g cartridge (Biotage); A: DCM, B: methanol; 20 column volumes (CV) linear gradient from 0% to 30% B) to give a yellow solid that still contained Et_3N and HOBT. The mixture was dissolved in a minimal amount of water and passed through a pre-equilibrated C18 Sep Pak column (5g, Waters, USA) twice. The product was eluted with 1:4 (v/v) acetonitrile: water and fractions containing sugar were collected and lyophilized to give **4a** (29.4 mg, 0.11 mmol, 23 %, 1:1 α:β) as a white powder. ^{1}H NMR (400 MHz, D_2O) δ 5.22 (d, J = 3.5 Hz, 0.5H), 4.76 (d, J = 5.3 Hz, 0.5H), 4.21 (m, J = 7.0, 4.8 Hz, 1H), 3.95 – 3.89 (m, 1H), 3.89 – 3.84 (m, 1H), 3.83 – 3.67 (m, 2H), 3.64 – 3.40 (m, 2H), 1.48 (dd, J = 7.0, 1.1 Hz, 3H); ^{13}C NMR (151 MHz, D_2O) δ 174.2, 173.9, 94.7, 90.7, 75.9, 73.5, 71.6, 70.5, 70.0, 69.9, 60.7, 60.6, 58.8, 58.4, 56.7, 54.1, 16.7, 16.6. MS (ESI) calcd. for $C_9H_{16}N_4O_6$ (M-H$^+$) 275.11 found 275.01 *m/z*.

2-(4-Azidobutanamido)-2-deoxy-D-glucopyranose (6a)

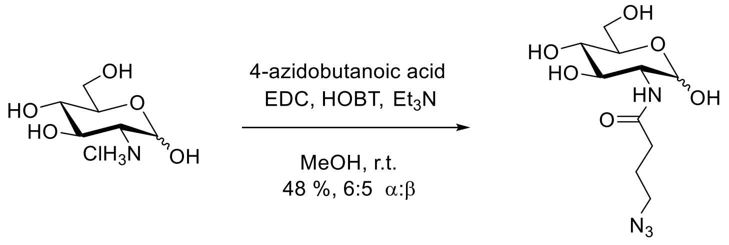

6a

To a stirred solution of 4-azidobutanoic acid (Fluorochem Ltd, UK) (146 mg, 1.1 mmol, 2.4 eq.) in anhydrous methanol (2.5 mL) were added EDC (195 mg, 1.25 mmol, 2.67 eq.) and HOBT (76 mg, 0.56 mmol, 1.2 eq.) and left to activate for 10 min at r.t. A solution of D-glucosamine hydrochloride (100 mg, 0.47 mmol, 1 eq.) and Et_3N (175 µL, 1.25 mmol, 2.67 eq.) in anhydrous methanol (2.5 mL) was added and the final solution was stirred at r.t. for 16 h. On completion, the solvent was evaporated, the dried residue dissolved in a minimal amount of water and passed through a pre-equilibrated C18 Sep Pak column twice. Product was eluted with acetonitrile: water 1:4 (v/v) and fractions containing sugar were collected and lyophilized to give **6a** (65 mg, 0.22 mmol, 48 %, 6:5: α:β) as a white powder. ^{1}H NMR (400 MHz, D_2O) δ 5.21 (d, J = 3.5 Hz, 0.55H), 4.72 (d, J = 8.4 Hz, 0.45H), 3.93 – 3.82 (m, 2H), 3.82 – 3.67 (m, 2H), 3.58 – 3.43 (m, 2H), 3.38 (td, J = 6.8, 1.6 Hz, 2H), 2.40 (td, J = 7.3, 5.3 Hz, 2H), 1.90 (m, J = 7.0 Hz, 2H); ^{13}C NMR (100 MHz, D_2O) δ 176.4, 176.2, 94.9, 90.8, 75.9, 73.8, 71.5, 70.6, 70.1, 69.9, 60.7, 60.6, 56.5, 54.0, 50.4, 50.2, 33.1, 32.8, 24.5, 24.5. MS (ESI) calcd. for $C_{10}H_{18}N_4O_6$ (M-H$^+$) 289.12 found 289.05 *m/z*.

2-[(*S*)-2-Azidobutanamido]-2-deoxy-1,3,4,6-tetra-*O*-acetyl-D-glucopyranose (9b)

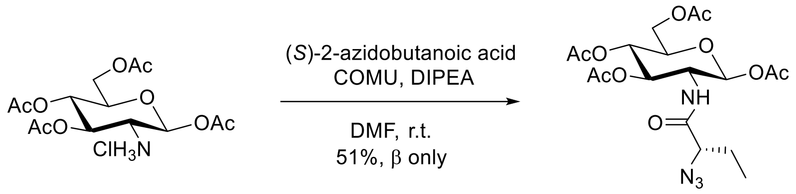

SI-1 **9b**

To a stirred solution of **SI-1** (160 mg, 0.4 mmol) in DMF (3 mL) were added (*S*)-2-azidobutanoic acid[24] (57 mg, 0.44 mmol, 1.1. eq.), DIPEA (209 µL, 1.2 mmol, 3 eq.) and COMU (171 mg, 0.4 mmol, 1 eq.). The solution was left to stir at r.t. for 16 h. After completion, the reaction mixture was diluted with EtOAc (50 mL) and washed with 0.1 M HCl (2x40 mL), sat. aq. $NaHCO_3$ (2x 40 mL) and brine. The organic layer was dried, concentrated, and purified on medium pressure flash chromatography (Sfär Silica D Duo 10 g cartridge; A: cyclohexane, B: EtOAc; 10 CV linear gradient from 0% to 40% B) to give **9b** (94 mg, 0.21 mmol, 51 %, β-anomer only) as a yellow oil. ^{1}H NMR (400 MHz, $CDCl_3$) δ 6.42 (d, *J* = 9.4 Hz, 1H), 5.74 (d, *J* = 8.7 Hz, 1H), 5.23 (dd, *J* = 10.5, 9.3 Hz, 1H), 5.14 (t, *J* = 9.5 Hz, 1H), 4.31 – 4.19 (m, 2H), 4.13 (dd, *J* = 12.5, 2.3 Hz, 1H), 3.91 (dd, *J* = 6.6, 4.9 Hz, 1H), 3.82 (m, *J* = 9.8, 4.6, 2.3 Hz, 1H), 2.09 (d, *J* = 1.9 Hz, 6H), 2.04 (d, *J* = 2.8 Hz, 6H), 1.94 – 1.77 (m, 2H), 0.95 (t, *J* = 7.4 Hz, 3H); ^{13}C NMR (100 MHz, $CDCl_3$) δ 171.0, 170.8, 169.7, 169.4, 169.3, 92.4, 73.1, 72.2, 67.8, 65.6, 61.7, 53.2, 25.4, 20.9, 20.9, 20.7, 20.7, 9.5. MS (ESI) calcd. for $C_{16}H_{23}N_4O_8^+$ (oxocarbenium ion) 399.15 found 399.08 *m/z*.

2-[(*S*)-2-Azidobutanamido]-2-deoxy-D-glucopyranose (9a)

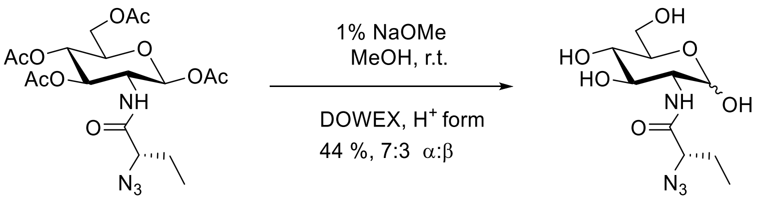

9b **9a**

Ester **9b** (50 mg, 0.11 mmol) was dissolved in 1% (w/v) sodium methoxide in methanol (1.5 mL) and stirred at r.t for 5 h. On completion, the reaction mixture was added to DOWEX 50W8 (H^+ form, 50-100mesh, Serva, Germany), left for 10 min and filtered. The filtrate was concentrated, lyophilized and purified by flash chromatography (Sfär Silica D Duo 5 g cartridge (Biotage); A: DCM, B: methanol; 10 CV linear gradient from 0% to 30% B) to give **9a** (13.9 mg, 0.05 mmol, 44 %, 7: 3 α:β) as a white powder. ^{1}H NMR (400 MHz, CD_3OD) δ 5.12 (d, *J* = 3.5 Hz, 0.7H), 4.65 (d, *J* = 8.3 Hz, 0.3H), 4.06 – 3.41 (m, 6H), 3.40 – 3.34 (m,

1H), 2.01 – 1.71 (m, 2H), 1.02 (td, J = 7.4, 5.2 Hz, 3H); ^{13}C NMR (100 MHz, CD_3OD) δ 172.6, 96.8, 92.5, 78.0, 75.8, 73.2, 72.6, 72.5, 66.0, 65.5, 62.8, 58.8, 55.9, 26.3, 26.1, 10.2, 10.2. MS (ESI) calcd. for $C_{10}H_{18}N_4O_6$ (M-H^+) 289.12 found 289.1 *m/z*.

2-(4-Azidopentanamido)-2-deoxy-D-glucopyranose (10a)

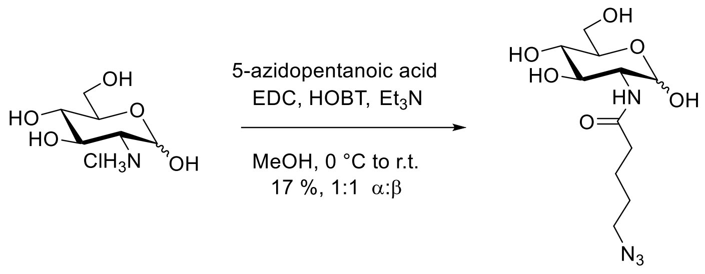

10a

To a stirred solution of 5-azidopentanoic acid (186 mg, 1.3 mmol, 1.4 eq.) in anhydrous methanol (10 mL) were added D-glucosamine hydrochloride (200 mg, 0.93 mmol, 1 eq.) and Et_3N (335 µL, 2.4 mmol, 2.78 eq.). The solution was cooled to 0 °C and EDC (173 mg, 1.1. mmol, 1.2 eq.) and HOBT (149 mg, 1.1 mmol, 1.2 eq.) were added. The reaction was warmed to r.t. and stirred for 16 h. On completion, the solvent was evaporated, and the crude mixture purified on medium pressure flash chromatography and pre-packed silica column (Sfär Silica D Duo 10 g cartridge; A: DCM, B: methanol; 20 CV linear gradient from 0% to 50% B) to give the amide as yellow solid that still contained Et_3N and HOBT. The mixture was further purified on Agilent 1260 Infinity II MDAP system (Agilent Technologies, UK) equipped with a 5 Prep-C18 (100Å, 5 µm, 21.2 mm x 50 mm, Agilent) column and buffers: A: water containing 0.1% formic acid; B: acetonitrile containing 0.1% formic acid. Fractions containing pure product were collected and lyophilized to give **10a** (47.6 mg, 0.156 mmol, 17 %, 0.55:0.45 α:β) as a white powder. ^1H NMR (400 MHz, D_2O) δ 5.21 (d, J = 3.5 Hz, 0.55H), 4.71 (d, J = 8.4 Hz, 0.45H), 3.94 – 3.83 (m, 2H), 3.81 – 3.66 (m, 2H), 3.58 – 3.41 (m, 2H), 3.35 (td, J = 6.6, 1.1 Hz, 2H), 2.39 – 2.32 (m, 2H), 1.74 – 1.59 (m, 4H); ^{13}C NMR (100 MHz, D_2O) δ 177.2, 176.9, 94.9, 90.8, 75.9, 73.8, 71.5, 70.5, 70.1, 69.9, 60.7, 60.6, 56.5, 54.0, 50.7 (d), 35.4, 35.0, 27.3, 27.3, 22.5 (d). MS (ESI) calcd. for $C_{11}H_{20}N_4O_6$ (M-H^+) 303.14 found 303.0 *m/z*.

2-(4-Pentynamido)-2-deoxy-1,3,4,6-tetra-*O*-acetyl-D-glucopyranose (SI-2)

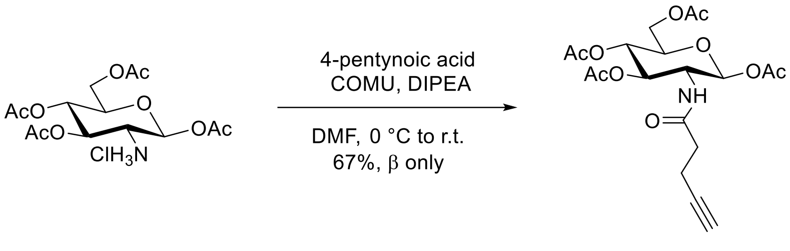

SI-1 **SI-2**

A mixture of **SI-1** (768 mg, 2 mmol), 4-pentynoic acid (196 mg, 2 mmol, 1 eq.) and DIPEA (1.045 mL, 6 mmol, 3 eq.) in DMF (15 mL) was cooled to 0 °C. COMU (1.712 g, 4 mmol, 2 eq.) was added and the reaction mixture stirred at 0 °C for 1 h. The solution was warmed to r. t. and stirred for another 3 h. The mixture was diluted with EtOAc (100 mL), and the organic layer was washed with 1 M aq. HCl (2x50 mL), sat. $NaHCO_3$ (2x50 mL) and brine, dried over $MgSO_4$, filtered, and concentrated. The residue was purified by medium-pressure flash chromatography (25g SNAP-KP-SIL (Biotage); A: cyclohexane, B: EtOAc; 20 CV linear gradient from 20% to 60% B) to give the tetraacetate amide **SI-2** (597 mg, 1.4 mmol, 70%, β-anomer only) as a white solid. ^{1}H NMR (400 MHz, $CDCl_3$) δ 5.70 (d, *J* = 8.8 Hz, 1H), 5.67 (d, *J* = 9.5 Hz, 1H), 5.21 – 5.08 (m, 2H), 4.38 – 4.24 (m, 2H), 4.13 (dd, *J* = 12.5, 2.2 Hz, 1H), 3.81 (m, *J* = 9.4, 4.6, 2.3 Hz, 1H), 2.48 (m, *J* = 6.8, 6.2, 1.8 Hz, 2H), 2.36 – 2.28 (m, 2H), 2.11 (s, 3H), 2.09 (s, 3H), 2.04 (d, *J* = 0.7 Hz, 6H), 1.97 (t, *J* = 2.6 Hz, 1H); ^{13}C NMR (100 MHz, $CDCl_3$) δ 171.3, 171.1, 170.8, 169.7, 169.4, 92.7, 82.6, 77.4, 73.1, 72.6, 69.7, 67.9, 61.8, 53.1, 35.6, 21.1, 20.9 (d, *J* = 1.6 Hz), 20.7, 15.0. MS (ESI) calcd. for $C_{17}H_{22}NO_8^+$ (oxocarbenium ion) 368.13 found 368.2 *m/z*.

2-(4-Pentynamido)-2-deoxy-3,4,6-tri-*O*-acetyl-D-glucopyranose (SI-3)

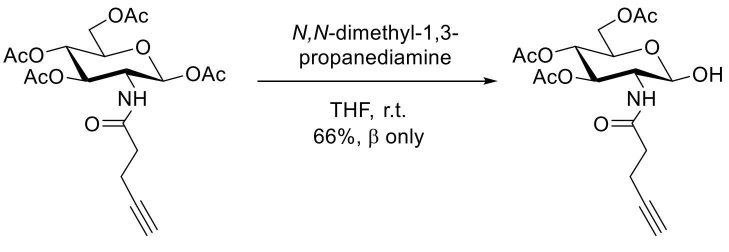

SI-2 **SI-3**

To a stirred solution of tetraacetate **SI-2** (597 mg, 1.4 mmol) in THF (7 mL) was added 3-(*N,N*-dimethylamino)-1-propylamine (0.478 mL, 4.2 mmol, 3 eq.). The reaction was stirred at r.t. for 2 h, diluted with DCM (100 mL) and washed with 1 M aq. HCl (2x50 mL) and brine (50 mL). The organic phase was dried over $MgSO_4$, filtered, and concentrated. The residue was

purified by medium-pressure flash chromatography (25 g SNAP cartridge; A: cyclohexane, B: EtOAc; 20 CV linear gradient from 20% to 70% B) to give triacetate **SI-3** (357 mg, 0.92 mmol, 66%, β-anomer only) as a clear oil. ^{1}H NMR (400 MHz, $CDCl_3$) δ 5.98 (d, *J* = 9.3 Hz, 1H), 5.40 – 5.21 (m, 2H), 5.13 (t, *J* = 9.5 Hz, 1H), 4.33 (m, *J* = 10.8, 9.3, 3.6 Hz, 1H), 4.24 – 4.11 (m, 3H), 2.49 (m, *J* = 6.9, 2.6, 1.1 Hz, 2H), 2.38 (m, *J* = 8.2, 6.4, 2.7 Hz, 2H), 2.10 (s, 3H), 2.03 (s, 3H), 2.02 (s, 3H), 1.99 (t, *J* = 2.6 Hz, 1H); ^{13}C NMR (100 MHz, $CDCl_3$) δ 171.5, 171.2, 171.0, 169.6, 91.8, 82.7, 71.0, 69.6, 68.4, 67.8, 62.2, 52.3, 35.4, 21.0, 20.9, 20.8, 14.9. MS (ESI) calcd. for $C_{17}H_{22}NO_8^+$ (oxocarbenium ion) 368.13 found 368.3 *m/z*.

Bis-*O*-allyl 2-(4-pentynamido)-2-deoxy-3,4,6-tri-*O*-acetyl-α-D-glucopyranosyl phosphate (SI-4)

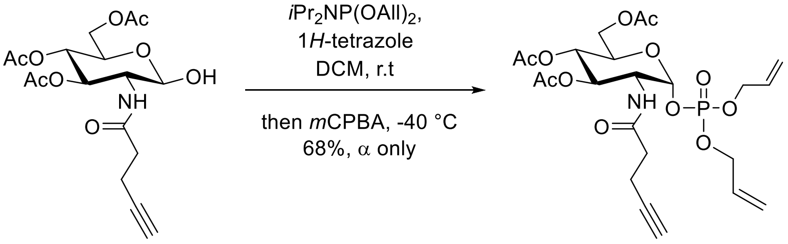

SI-3 **SI-4**

Lactol **SI-3** (0.92 mmol, 356 mg) and 1*H*-tetrazole (276 mg, 8.8 mL of 0.45M solution in ACN, 4 mmol) were co-evaporated with anhydrous toluene (8 mL), suspended in anhydrous toluene (8 mL) and sonicated for 1 h at r. t. in a bath sonicator. The solvent was evaporated, and the residue dissolved in anhydrous DCM (12 mL). The stirred solution was cooled to 0 °C and diallyl *N*,*N*-diisopropylphosphoramidite (400 µL, 1.46 mmol, 1.6 eq.) was added. After 30 min, the solution was cooled to -40 °C, and *m*CPBA (627 mg, 2.76 mmol, 3 eq.) was added. After 30 min, the reaction was quenched with 1 M aq. Na_2SO_3 (28 mL) and warmed to r. t. The mixture was diluted with DCM (40 mL) and the layers were separated. The organic phase was washed with sat. aq. $NaHCO_3$ (10 mL) and the combined aqueous phase was re-extracted with DCM (2x20 mL). The combined organic phase was washed with brine (20 mL), dried over $MgSO_4$, and concentrated. The residue was purified by medium-pressure flash chromatography (10g SNAP-KP-SIL; A: cyclohexane, B: EtOAc; 25 CV linear gradient from 20% to 70%B) to give phosphate **SI-4** (339 mg, 0.62 mmol, 68%, α-anomer only) as a clear oil. ^{1}H NMR (400 MHz, $CDCl_3$) δ 6.12 – 6.06 (m, 1H), 6.02 – 5.87 (m, 2H), 5.68 (dd, *J* = 6.1, 3.3 Hz, 1H), 5.44 – 5.35 (m, 2H), 5.34 – 5.27 (m, 2H), 5.24 (dd, *J* = 10.6, 9.4 Hz, 1H), 5.17 (t, *J* = 9.7 Hz, 1H), 4.64 – 4.54 (m, 4H), 4.48 – 4.40 (m, 1H), 4.24 (dd, *J* = 12.3, 4.1 Hz, 1H), 4.21 – 4.16 (m, 1H), 4.09 (dd, *J* = 12.2, 2.2 Hz, 1H), 2.51 – 2.44 (m, 2H), 2.39 – 2.32 (m, 2H), 2.07 (s, 3H), 2.02 (s, 3H), 2.01 (s, 3H), 1.99 (t, *J* = 2.6 Hz, 1H); ^{13}C

NMR (100 MHz, $CDCl_3$) δ 171.3, 171.2, 170.7, 169.3, 132.3, 132.0, 119.3, 96.2, 82.6, 70.2, 69.8, 69.6, 69.0, 69.0, 68.9, 68.9, 67.6, 61.6, 51.9, 35.2, 20.8, 20.7, 14.7. MS (ESI) calcd. for $C_{17}H_{22}NO_8^+$ (oxocarbenium ion) 368.13 found 369.04 *m/z*.

Uridine 5'-diphospho-2-(4-pentynamido)-2-deoxy-α-D-glucopyranoside ammonium salt (SI-5)

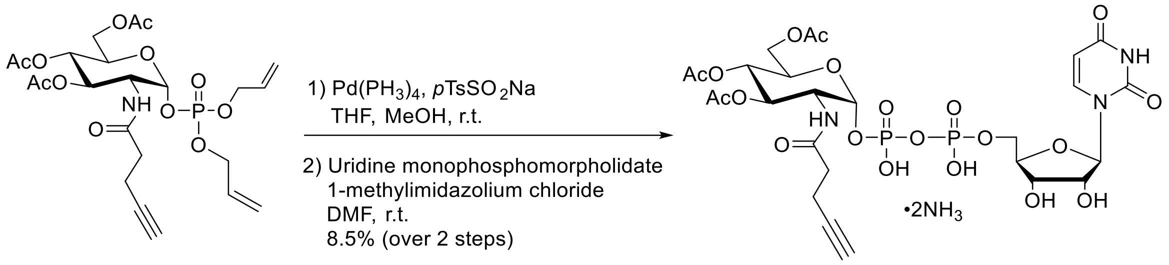

SI-4 **SI-5**

To a stirred solution of diallyl phosphotriester **SI-4** (100 mg, 189 µmol) in THF:MeOH (1:1 v/v, 3.8 mL) were added tetrakis(triphenylphosphine)palladium (22 mg, 19 µmol) and sodium *para*-toluoenesulfinate (137 mg, 756 µmol, 4 eq.). The mixture was stirred at r.t. for 16 h, and the solvents were evaporated. The residue was co-evaporated with anhydrous toluene (2x10 mL) and dissolved in DMF (3.8 mL). Uridine monophosphomorpholidate (208 mg, 308 µmol, 1.6 eq.) and 1-methylimidazolium chloride (122 mg, 104 µmol,) were added and the reaction was stirred at r. t. for 16 h. Additional uridine monophosphomorpholidate (208 mg) and 1- methylimidazolium chloride (122 mg) were added and reaction left to stir at 40 °C for 16 h. After no further progress of the reaction was observed, the mixture was concentrated and purified by medium-pressure flash chromatography (30 g SNAP C18 column; A: 5 mM ammonium acetate, B: MeOH; 15 CV linear gradient from 0% to 60% B, then 5 CV 100% B) followed by size-exclusion chromatography (Bio-Gel P-2, Bio-Rad, USA; 5 mM ammonium acetate: isopropanol (4:1 v/v)). The fractions were concentrated and lyophilized to give pyrophosphate **SI-5** as the intermediary ammonium salt (12.7 mg, 16 µmol, 8.5% over two steps) as a white foam. ^1H NMR (400 MHz, CD_3OD) δ 8.09 (d, J = 8.2 Hz, 1H), 5.96 (t, J = 4.4 Hz, 1H), 5.85 (d, J = 8.1 Hz, 1H), 5.61 (dd, J = 7.2, 3.4 Hz, 1H), 5.28 (t, J = 10.0 Hz, 1H), 5.11 (t, J = 9.8 Hz, 1H), 4.43 – 4.27 (m, 7H), 4.18 (d, J = 12.9 Hz, 2H), 2.53 (d, J = 6.1 Hz, 2H), 2.48 – 2.42 (m, 2H), 2.22 (t, J = 2.6 Hz, 1H), 2.06 (s, 3H), 1.99 (s, 3H), 1.94 (s, 3H); ^{13}C NMR (100 MHz, CD_3OD) δ 174.7, 172.5, 172.0, 171.4, 166.3, 152.6, 127.9, 103.2, 95.9, 90.3, 83.9, 75.8, 73.0, 71.0, 70.1, 69.9, 69.8, 68.8, 65.6, 45.0, 35.8, 21.0, 20.9, 20.7, 20.6, 15.5. MS (ESI) calcd. for $C_{26}H_{35}N_3O_{20}P_2$ (M-H^+) 770.13 found 770.12 *m/z*.

Uridine 5'-diphospho-2-(4-pentynamido)-2-deoxy-α-D-glucopyranoside sodium salt (7)

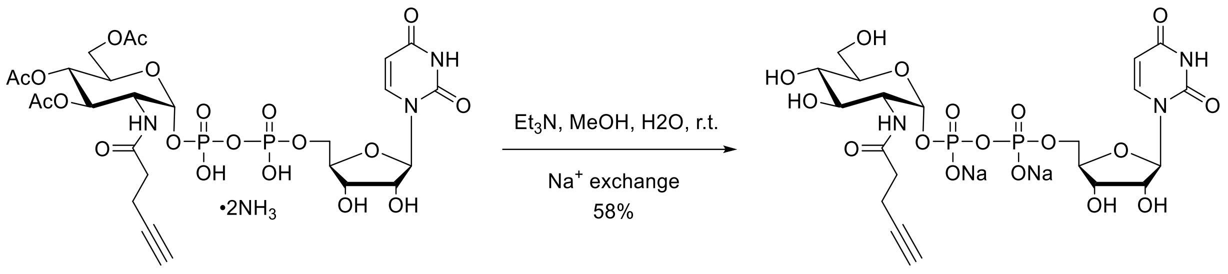

SI-5 UDP-GlcNAlk **(7)**

To a stirred solution of triester **SI-5** (5.6mg, 7.2 μmol) in MeOH:water 5:2 (v/v) (1.5 mL) was added triethylamine (300 μL). The reaction was stirred at r. t. for 16 h. The residue was concentrated and lyophilized repeatedly. The residue was passed through a short (4 g resin) ion exchange column of Dowex 50W X8 Na^+ form (Serva, Heidelberg, Germany), and concentrated. The lyophilized residue was passed through reverse-phase solid-phase extraction (Sep-Pak C18, 5g, Waters), eluted with water and lyophilized to give UDP-GlcNAc analog **7** as the disodium salt (3.4 mg, 4.2 μmol, 58%) as a white solid. Variable temperature NMR (VT NMR) confirmed that peaks at 7.84 ppm and 5.95 ppm are thought to result from tautomerization of the uracil ring. ^{1}H NMR (600 MHz, D_2O) δ 7.97 (d, J = 8.2 Hz, 1H), 6.01 – 5.93 (m, 2H), 5.52 (dt, J = 6.6, 3.3 Hz, 1H), 4.54 – 4.35 (m, 2H), 4.30 (m, J = 5.4, 2.6 Hz, 1H), 4.28 – 4.12 (m, 2H), 4.04 (m, J = 10.1, 3.4 Hz, 1H), 3.95 (m, J = 10.1, 4.3, 2.4 Hz, 1H), 3.88 (dd, J = 12.5, 2.3 Hz, 1H), 3.85 – 3.63 (m, 3H), 3.60 – 3.52 (m, 1H), 2.65 – 2.54 (m, 2H), 2.53 – 2.48 (m, 2H); ^{13}C NMR (151 MHz, D_2O) δ 175.2, 166.7, 152.2, 141.6, 102.7, 94.6, 92.0, 88.5, 83.2 (d, J = 9.4 Hz), 75.9, 73.8, 73.0, 70.9, 69.6, 69.5, 64.9 (d, J = 5.5 Hz), 60.4 (d, J = 19.9 Hz), 53.6, 34.3, 14.3; ^{31}P NMR (162 MHz, D_2O) δ -11.40 (m, J = 23.7, 5.2, 2.5 Hz), -13.22 (m, J = 21.0, 7.1, 3.0 Hz). MS (ESI) calcd. for $C_{20}H_{28}N_3O_{17}P_2$ (M-H$^+$) 645.4 found 645.1 *m/z*.

2-(5-Hexynamido)-2-deoxy-1,3,4,6-tetra-*O*-acetyl-D-glucopyranose (SI-6)

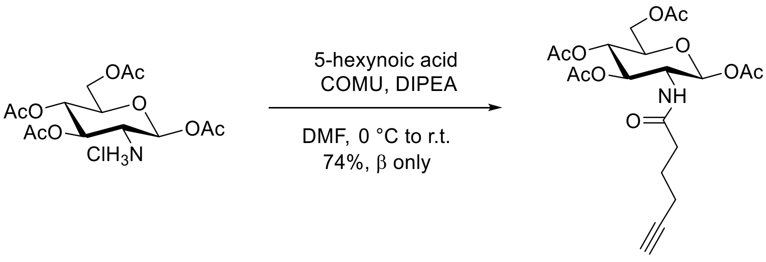

SI-1 **SI-6**

A mixture of **SI-1** (768 mg, 2 mmol), 5-hexynoic acid (224 µL, 2 mmol, 1 eq,) and DIPEA (1.045 mL, 6 mmol, 3 eq.) in DMF (16 mL) was cooled to 0 °C. COMU (1.712 g, 4 mmol, 2 eq.) was added and the reaction mixture stirred at 0 °C for 1 h. The solution was warmed to r. t. and stirred for another 16 h. The mixture was diluted with EtOAc (100 mL), and the organic layer was washed with 1 M aq. HCl (2x50 mL), sat. $NaHCO_3$ (2x50 mL) and brine, dried over $MgSO_4$, filtered, and concentrated. The residue was purified by medium-pressure flash chromatography (25g SNAP-KP-SIL; A: cyclohexane, B: EtOAc; 10 CV linear gradient from 0% to 100% B) to give tetraacetate amide **SI-6** (657 mg, 1.4 mmol, 74%, β-anomer only) as a yellow oil. ^{1}H NMR (400 MHz, $CDCl_3$) δ 5.71 (d, *J* = 8.8 Hz, 1H), 5.56 (d, *J* = 9.5 Hz, 1H), 5.22 – 5.07 (m, 2H), 4.34 – 4.28 (m, 1H), 4.26 (d, *J* = 4.7 Hz, 1H), 4.12 (dd, *J* = 12.5, 2.3 Hz, 1H), 3.81 (m, *J* = 9.6, 4.6, 2.3 Hz, 1H), 2.29 – 2.24 (m, 2H), 2.21 (td, *J* = 6.8, 2.7 Hz, 2H), 2.11 (s, 3H), 2.09 (s, 3H), 2.04 (d, *J* = 1.4 Hz, 6H), 1.96 (t, *J* = 2.7 Hz, 1H), 1.79 (m, *J* = 6.8 Hz, 2H); ^{13}C NMR (100 MHz, $CDCl_3$) δ 172.3, 171.3, 170.8, 169.6, 169.4, 92.7, 83.1, 73.1, 72.6, 69.6, 67.9, 61.8, 53.0, 34.9, 23.9, 21.0, 20.9, 20.8, 20.7, 17.6. MS (ESI) calcd. for $C_{18}H_{24}NO_8^+$ (oxocarbenium ion) 382.15 found 382.3 *m/z*.

2-(5-Hexynamido)-2-deoxy-3,4,6-tri-*O*-acetyl-D-glucopyranose (SI-7)

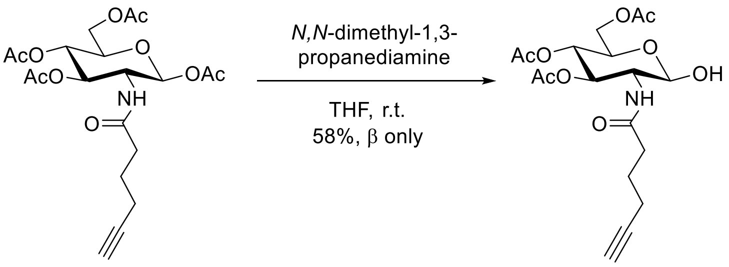

SI-6 **SI-7**

To a stirred solution of tetraacetate **SI-6** (656 mg, 1.49 mmol) in THF (7.5 mL) was added 3-(dimethylamino)-1-propylamine (0.498 mL, 4.47 mmol, 3 eq.). The reaction was stirred at r.t. for 2 h, diluted with DCM (100 mL) and washed with 1 M aq. HCl (2x50 mL) and brine (50

mL). The organic phase was dried over $MgSO_4$, filtered, and concentrated. The residue was purified by medium-pressure flash chromatography (25 g SNAP cartridge; A: cyclohexane, B: EtOAc; 20 CV linear gradient from 20% to 70% B) to give tetraacetate **SI-7** (347 mg, 0.87 mmol, 58%, β-anomer only) as a clear oil. ^{1}H NMR (400 MHz, $CDCl_3$) δ 5.81 (d, *J* = 9.3 Hz, 1H), 5.37 – 5.24 (m, 2H), 5.18 – 5.07 (m, 1H), 4.32 (m, *J* = 10.8, 9.3, 3.6, 1.5 Hz, 1H), 4.24 – 4.18 (m, 2H), 4.16 – 4.11 (m, 1H), 2.30 (td, *J* = 7.3, 3.1 Hz, 2H), 2.26 – 2.20 (m, 2H), 2.10 (d, *J* = 2.1 Hz, 3H), 2.04 (s, 3H), 2.03 (s, 3H), 1.98 (t, *J* = 2.6 Hz, 1H), 1.82 (m, *J* = 7.1, 1.6 Hz, 2H); ^{13}C NMR (100 MHz, $CDCl_3$) δ 172.3, 171.4, 170.8, 169.4, 91.8, 83.1, 70.8, 69.4, 68.2, 67.8, 62.1, 52.1, 34.9, 24.0, 20.8, 20.8, 20.6, 17.7. MS (ESI) calcd. for $C_{18}H_{24}NO_8^+$ (oxocarbenium ion) 382.15 found 382.3 *m/z*.

Bis-*O*-allyl 2-(5-hexynamido)-2-deoxy-3,4,6-tri-*O*-acetyl-α-D-glucopyranosyl phosphate (SI-8)

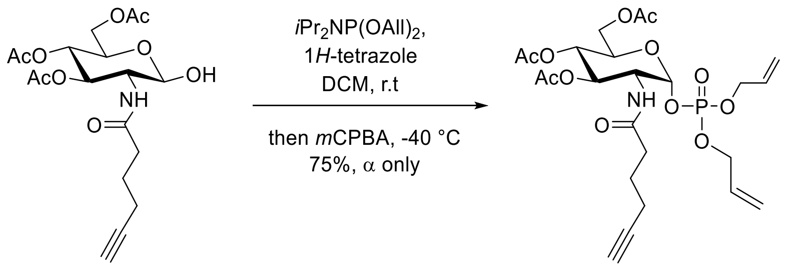

SI-7 SI-8

Lactol **SI-3** (200 mg 0.5 mmol) and 1*H*-tetrazole (5 mL of 0.45M solution in ACN, 4 mmol, 2.5eq.) were co-evaporated with anhydrous toluene (4.5 mL), suspended in anhydrous toluene (4.5 mL) and sonicated for 1 h at r.t. in a bath sonicator. The solvent was evaporated, and the residue dissolved in anhydrous DCM (7.5 mL). The stirred solution was cooled to 0 °C and diallyl *N*,*N*-diisopropylphosphoramidite (215 μL, 0.8 mmol, 1.6 eq.) was added. After 30 min, the solution was cooled to -40 °C, and *m*CPBA (338 mg, 1.5 mmol, 3 eq.) was added. After 30 min, the reaction was quenched with 1 M aq. Na_2SO_3 (20 mL) and warmed to r. t. The mixture was diluted with DCM (50 mL) and the layers were separated. The combined organic phase was washed with sat. $NaHCO_3$ (2x 20 mL) and brine (20 mL), dried over $MgSO_4$, and concentrated. The residue was purified by medium-pressure flash chromatography (10g SNAP-KP-SIL; A: cyclohexane, B: EtOAc; 20 CV linear gradient from 20% to 70%B) to give phosphate **SI-8** (211 mg, 0.38 mmol, 75%, α-anomer only) as a clear oil. ^{1}H NMR (400 MHz, $CDCl_3$) δ 6.03 – 5.88 (m, 2H), 5.83 (d, *J* = 9.1 Hz, 1H), 5.70 (dd, *J* = 6.0, 3.3 Hz, 1H), 5.40 (m, *J* = 17.0, 12.5, 1.5 Hz, 2H), 5.31 (m, *J* = 11.5, 10.4, 1.2 Hz, 2H), 5.24 (dd, *J* = 10.4, 9.4 Hz, 1H), 5.21 – 5.16 (m, 1H), 4.66 – 4.55 (m, 4H), 4.43 (m, *J* = 10.5,

9.1, 3.2 Hz, 1H), 4.25 (dd, *J* = 12.3, 4.1 Hz, 1H), 4.20 – 4.16 (m, 1H), 4.09 (dd, *J* = 12.3, 2.2 Hz, 1H), 2.28 (td, *J* = 7.4, 1.7 Hz, 2H), 2.22 (m, *J* = 7.2, 2.7 Hz, 2H), 2.08 (s, 3H), 2.04 (s, 3H), 2.03 (s, 3H), 1.96 (m, *J* = 2.6 Hz, 1H), 1.88 – 1.74 (m, 2H); ^{13}C NMR (100 MHz, $CDCl_3$) δ 172.4, 171.4, 170.7, 169.3, 132.3 (d, *J* = 6.4 Hz), 132.1 (d, *J* = 6.5 Hz), 119.3, 119.3, 96.3 (d, *J* = 6.3 Hz), 83.2, 70.2, 69.9, 69.5, 69.0 (d, *J* = 5.3 Hz), 68.9 (d, *J* = 5.4 Hz), 67.5, 61.6, 52.0 (d, *J* = 7.9 Hz), 34.9, 24.0, 20.8, 20.8, 20.7, 17.9. MS (ESI) calcd. for $C_{18}H_{24}NO_8^+$ (oxocarbenium ion) 382.15 found 382.3 *m/z*.

Uridine 5'-diphospho-2-(4-hexynamido)-2-deoxy-α-D-glucopyranoside sodium salt (8)

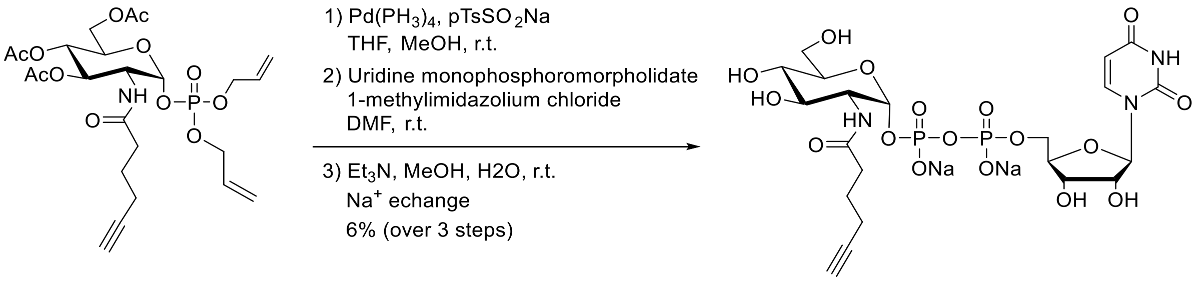

SI-8 UDP-GlcN6yne **(8)**

To a stirred solution of diallyl phosphotriester **SI-8** (100 mg, 189 µmol) in THF:MeOH (1:1 v/v, 3.8 mL) were added tetrakis(triphenylphosphine)palladium (21 mg, 18 µmol) and sodium *para*-toluoenesulfinate (132 mg, 746 µmol, 4 eq.). The mixture was stirred at r.t. for 16 h, and the solvents were evaporated. The residue was co-evaporated with anhydrous toluene (2x10 mL) and dissolved in DMF (3.8 mL). Uridine monophosphomorpholidate (202 mg, 294 µmol, 1.6 eq.) and 1-methylimidazolium chloride (117 mg, 104 µmol) were added and the reaction was stirred at r. t. for 16 h. Additional uridine monophosphomorpholidate (208 mg) and 1-methylimidazolium chloride (117 mg) were added and reaction left to stir at 40 °C for further 16 h. After no further progress of the reaction was observed, the mixture was concentrated and purified by medium-pressure flash chromatography (30 g SNAP C18 column; A: 5 mM ammonium acetate, B: MeOH; 15 CV linear gradient from 0% to 60% B, then 5 CV 100% B) and then by size-exclusion chromatography (Bio-Gel P-2, Bio-Rad, USA; 5 mM ammonium acetate: isopropanol (v/v) 4:1). The fractions containing product were concentrated and lyophilized to give the intermediary triester as an ammonium salt. To a stirred solution of the intermediary triester in methanol: water (5:1 v/v, 1.8 mL) was added triethylamine (347 µL) and the reaction was stirred at r.t. After 16 h, the reaction mixture was concentrated, dissolved in a minimal amount of water and passed through a short (4 g resin) ion exchange column of Dowex 50W X8 Na$^+$ form. The concentrated residue was passed through reverse-phase solid-phase extraction (Sep-Pak C18, 5g, Waters), eluted with water

and lyophilized to give compound **8** (8 mg, 11.4 µmol, 6% over 3 steps) as a white solid. ^{1}H NMR (700 MHz, D_2O) δ 7.98 – 7.80 (m, 1H), 5.97 (m, *J* = 24.0, 19.9, 3.6 Hz, 2H), 5.52 (dt, *J* = 7.6, 3.8 Hz, 1H), 4.43 – 4.16 (m, 5H), 4.06 – 3.78 (m, 6H), 3.56 (dd, *J* = 13.0, 6.7 Hz, 1H), 2.47 (tq, *J* = 12.2, 5.4 Hz, 2H), 2.38 – 2.24 (m, 2H), 1.90 – 1.79 (m, 2H); ^{13}C NMR (176 MHz, D_2O) δ 176.8, 165.5, 160.7, 141.4, 102.8, 94.6, 88.5, 84.5, 83.1, 75.9, 73.8, 73.0, 70.9, 69.6, 69.6, 65.0, 60.3, 53.5, 34.6, 24.2, 17.1. MS (ESI) calcd. for $C_{21}H_{30}N_3O_{17}P_2$ (M-H$^+$) 658.11 found 658.1 *m/z*.

Chemoenzymatic synthesis of chemically modified UDP-GlcNAc analogs

E.coli recombinant inorganic pyrophosphatase PmPpA from *Pasteurella multocida*, NahK from *B. longum* and GalNAc-1-phosphate uridyltransferase GlmU were purchased from Chemily (Peachtree Corners, USA).

Analytical scale (12 or 30 µL) one-pot multienzyme (OPME) reactions with NahK and AGX1 variants were performed to establish the optimal system for the generation of preparative scale UDP- sugars **4, 6, 9** and **10.** Each reaction mixture contained the corresponding GlcNAc analog (GalNAc and compounds **4a**, **6a**, **9a** and **10a**) (2.5 mM), ATP (5 mM), UTP (5 mM), $MgCl_2$ (5 mM), BSA (1 mg/mL), kinase NahK (2.5 µg), PmPpA (0.045U) and either AGX1 WT or one of the 6 recombinantly expressed AGX1 mutants (F381G, F381A, F383G, F383A, F381G/F383G and F381A/F383A) (500 nM) in 100 mM Tris-HCl pH 8. Reactions were run for 3 and 16 hours at 37 °C. At each end point, reactions were stopped by adding equal volume of ice- cold acetonitrile and further cooled on ice for 30 min. The cooled reaction mixtures were centrifugated at 16,200 × g for 30 min at 4 °C to remove the precipitated enzymes. The supernatants were analyzed on UPLC-MS (ACQUITY H-Class qDA, Waters, USA) equipped with ACQUITY UPLC BEH Glycan (1.7 µm 2.1x50 mm) column (Waters, USA) and gradient of 90-55% buffer B over 17 min at flow rate of 0.35 mL/min and column temperature at 50° C; buffer A: 10 mM ammonium formate pH 4.5, buffer B: 10 mM ammonium formate in 90:10 v/v acetonitrile: water). Product formation was monitored by absorption at 260 nm and further confirmed by mass detection in negative mode. Turnover (%) was determined by integration of the product's UV peaks and plotting it against a standard curve of 0-2.5 mM UDP-GlcNAc (Sigma- Aldrich) produced by serial dilution in final assay buffer. Blanks with the above reaction mixture and without enzymes were included in each set of experiments to account for potential noise signal at products' retention time.

Preparative chemoenzymatic synthesis for UDP-sugars **4**, **6**, **9** and **10** was carried out with various amounts of the corresponding sugar analogs (between 6.5 and 42 mg) and the rest of the above reaction mixture content scaled up accordingly.

Compounds **4** and **6** were prepared using NahK and GlmU. Calf-intestine alkaline phosphatase (CIAP) (Invitrogen, USA) (400U/mmol of substrate) was added after 16h and the reaction incubated at 37 °C for another 16 h. The reaction was then stopped by the addition of an equal volume of ice-cold ethanol, followed by further cooling on ice and centrifugation to precipitate the enzymes. The supernatant was collected, the ethanol removed under vacuum and the remaining aqueous solution dried by lyophilization. The dried product was dissolved in a minimal amount of water, passed through P-2 size-exclusion column (Bio-Gel P-2, Bio-Rad, USA) and eluted with 5 mM ammonium acetate: isopropanol 4:1(v/v). Fractions containing sugar were collected, dried, and further purified on HPLC (Perkin Elmer, USA) equipped with C8 column (Zorbax 300SB-C8, Agilent), flow rate 8 mL/min and buffer A: 5mM ammonium acetate, buffer B: acetonitrile. Product was eluted with a gradient of 0% buffer B over 15 min, followed by 0-10% buffer B over 20 min. The lyophilized fractions were passed through a short (4 g resin) ion exchange column of Dowex 50W X8 Na^+ form (Serva, Heidelberg, Germany), and concentrated to give pure **4** and **6** as a white solid with 17% and 21% yield respectively.

Compounds **9** and **10** were prepared using NahK and $AGX1^{F381A/F383A}$ and $AGX1^{F381G}$ respectively. After 24 h, an equal volume of ice-cold ethanol was added, and the reaction was cooled on ice and centrifugated to precipitate the enzymes. The supernatant was collected, the ethanol removed under vacuum and the remaining aqueous solution dried by lyophilization. The dried product was desalted by passing it through a pre-equilibrated 10 g C18 Sep Pak column (Waters, USA) and product eluted with 30% acetonitrile in water (v/v). Fractions containing the product were collected, lyophilized, and further purified on Agilent 1260 Infinity II MDAP system (Agilent Technologies, UK) equipped with HILIC XBridge® BEH Amide OBD™ Prep (130Å, 5 µm, 10 mm x 100 mm) for compound **4** and Agilent 5 Prep-C18 (100Å, 5 µm, 21.2 mm x 50 mm) column for compound **6**. Buffers used were: A: 10 mM ammonium formate at pH 4.5; B: 10 mM ammonium formate in 90:10 (v/v) acetonitrile: water for HILIC purifications and A: water containing 0.1% formic acid and B: acetonitrile containing 0.1% formic acid for C18 column purifications. The lyophilized final products were passed through a short (4 g resin) ion exchange column of Dowex 50W X8 Na^+ form and concentrated to give **9** and **10** as white solids in 30% and 67 % yield, respectively.

Uridine 5’-diphospho-2-((R)-azidopopionamido)-2-deoxy-α-D-glucopyranoside sodium salt (4)

white solid, 17%; ^{1}H NMR (400 MHz, D_2O) δ 7.96 (d, *J* = 8.1 Hz, 1H), 6.04 – 5.87 (m, 2H), 5.53 (dd, *J* = 7.1, 3.3 Hz, 1H), 4.54 – 4.35 (m, 2H), 4.29 (m, *J* = 5.0, 2.7 Hz, 1H), 4.27 – 4.11 (m, 3H), 4.02 (m, *J* = 10.5, 2.9 Hz, 1H), 3.95 (m, *J* = 10.1, 4.3, 2.3 Hz, 1H), 3.90 – 3.79 (m, 3H), 3.56 (m, *J* = 10.1, 9.1, 6.4 Hz, 1H), 1.49 (dd, *J* = 7.0, 3.5 Hz, 3H); ^{13}C NMR (151 MHz, D_2O) δ 173.9, 166.6, 152.1, 141.6, 102.5, 94.3, 88.5, 83.1, 73.7, 73.0, 70.7, 69.6, 69.5, 64.9, 60.3, 58.2, 53.8, 16.4. MS (ESI) calcd. for $C_{18}H_{26}N_6O_{17}P_2$ (M-H$^+$) 661.39 found 661.2 *m/z*.

Uridine 5’-diphospho-2-(4-azidobutanamido)-2-deoxy-α-D-glucopyranoside sodium salt (6)

white solid, 21%; ^{1}H NMR (400 MHz, D_2O) δ 7.97 (d, *J* = 8.1 Hz, 1H), 6.03 – 5.91 (m, 2H), 5.52 (dd, *J* = 7.1, 3.3 Hz, 1H), 4.54 – 4.08 (m, 5H), 4.02 (dt, *J* = 10.5, 3.1 Hz, 1H), 3.94 (m, *J* = 10.1, 4.3, 2.3 Hz, 1H), 3.88 (dd, *J* = 12.5, 2.4 Hz, 1H), 3.85 – 3.78 (m, 2H), 3.61 – 3.51 (m, 1H), 3.37 (td, *J* = 6.8, 2.5 Hz, 2H), 2.45 (dd, *J* = 8.4, 6.5 Hz, 2H), 1.95 – 1.85 (m, 2H); ^{13}C NMR (100 MHz, D_2O) δ 176.4, 166.4, 151.9, 141.6, 102.6, 94.6, 88.5, 83.2, 73.8, 73.0, 70.9, 69.6, 64.9, 60.3, 53.6, 53.5, 50.4, 32.8, 24.5. MS (ESI) calcd. for$C_{19}H_{28}N_6O_{17}P_2$ (M-H$^+$) 675.11 found 675.2 *m/z*.

Uridine 5’-diphospho-2-((*S*)-2-azidobutanamido)-2-deoxy-α-D-glucopyranoside sodium salt (9)

white solid, 30%; ^{1}H NMR (400 MHz, D_2O) δ 7.96 (d, *J* = 8.1 Hz, 1H), 6.07 – 5.88 (m, 2H), 5.55 (dd, *J* = 7.0, 3.3 Hz, 1H), 4.41 – 4.35 (m, 2H), 4.29 (d, *J* = 7.4 Hz, 1H), 4.26 – 4.17 (m, 1H), 4.13 (dd, *J* = 7.6, 5.7 Hz, 1H), 4.06 (dt, *J* = 10.6, 3.1 Hz, 1H), 3.96 (m, *J* = 10.2, 4.2, 2.3 Hz, 1H), 3.88 (dd, *J* = 12.5, 2.3 Hz, 1H), 3.86 – 3.79 (m, 2H), 3.63 – 3.52 (m, 1H), 1.95 – 1.76 (m, 2H), 0.99 (m, *J* = 7.4 Hz, 4H); ^{13}C NMR (151 MHz, D_2O) δ 173.4, 166.7, 152.1, 141.6, 102.7, 94.6, 88.4, 83.2, 83.2, 73.8, 73.1, 70.8, 69.7, 65.0, 63.9, 60.3, 53.6, 25.1, 9.1. MS (ESI) calcd. for $C_{19}H_{29}N_6O_{17}P_2$ (M-H$^+$) 675.11 found 675.12 *m/z*.

Uridine 5'-diphospho-2-(4-azidopentanamido)-2-deoxy-α-D-glucopyranoside sodium salt (10)

white solid, 67%; ^{1}H NMR (400 MHz, D_2O) δ 7.97 (d, *J* = 8.1 Hz, 1H), 6.04 – 5.92 (m, 2H), 5.52 (dd, *J* = 7.1, 3.2 Hz, 1H), 4.43 – 4.14 (m, 5H), 4.02 (dt, *J* = 10.5, 3.1 Hz, 1H), 3.95 (m, *J* = 10.2, 4.4, 2.4 Hz, 1H), 3.88 (dd, *J* = 12.5, 2.4 Hz, 1H), 3.85 – 3.78 (m, 2H), 3.56 (m, *J* = 10.0, 8.6, 6.5 Hz, 1H), 3.35 (dd, *J* = 7.3, 5.8 Hz, 2H), 2.40 (td, *J* = 7.1, 2.4 Hz, 2H), 1.77 – 1.56 (m, 4H); ^{13}C NMR (100 MHz, D_2O) δ 178.7, 166.8, 152.2, 141.6, 102.6, 94.6, 90.5, 83.1, 83.0, 73.7, 73.0, 70.8, 69.6, 65.0, 60.3, 53.5, 50.7, 35.0, 27.4, 22.4. MS (ESI) calcd. for $C_{20}H_{31}N_6O_{17}P_2$ (M-H$^+$) 689.13 found 689.15 *m/z*.

NMR characterization

^{1}H NMR, D_2O, 400 MHz

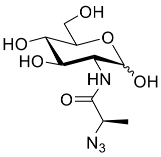

4a

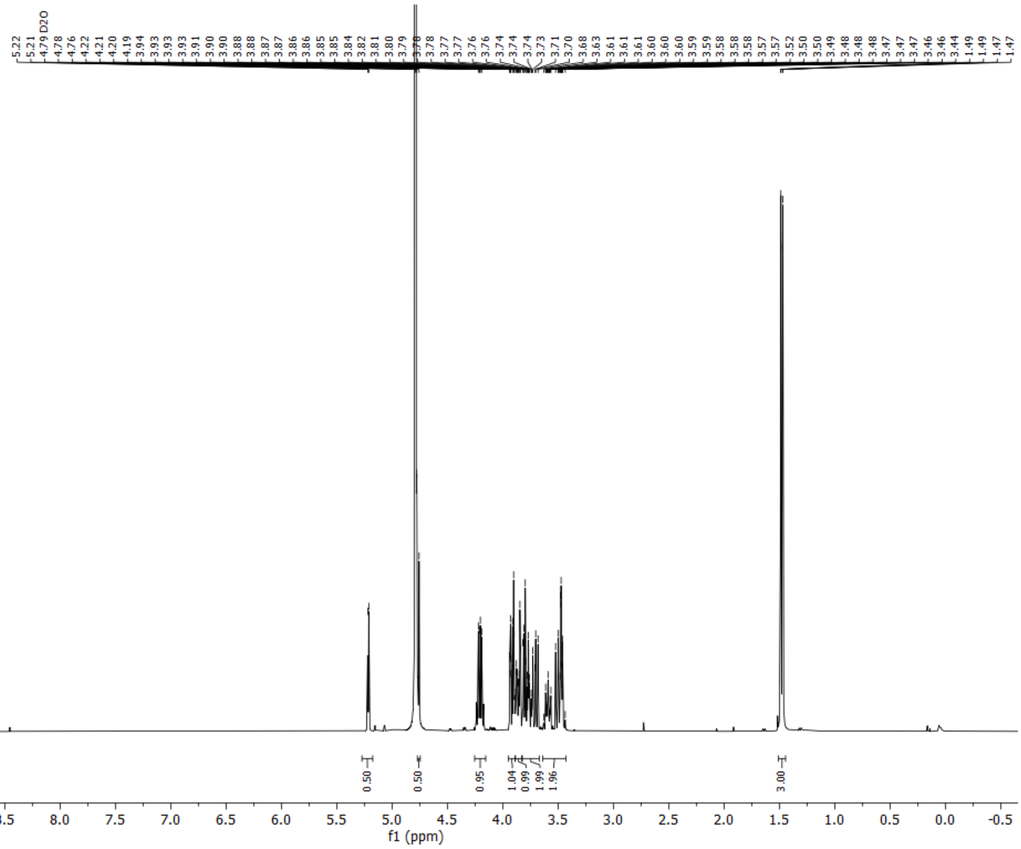

^{13}C NMR, D_2O, 600 MHz

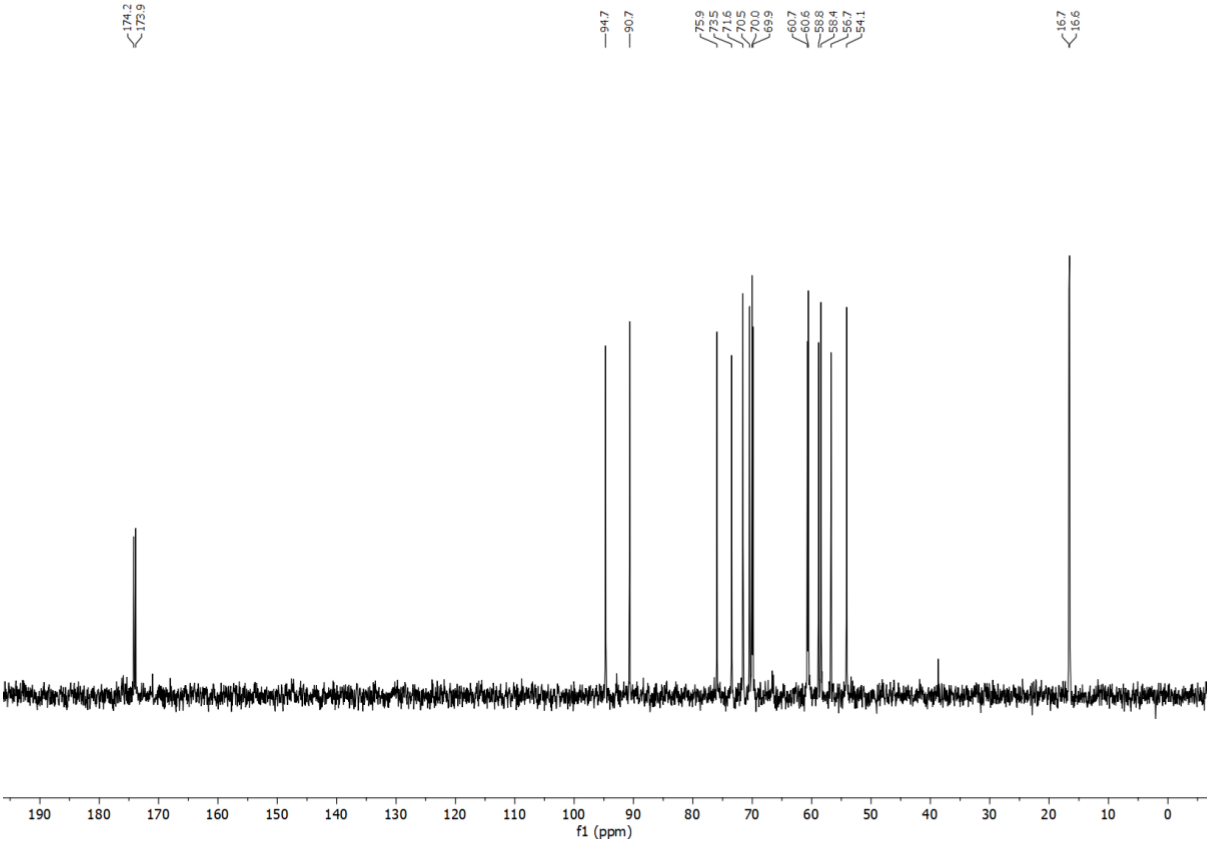

^{1}H NMR, D_2O, 400 MHz

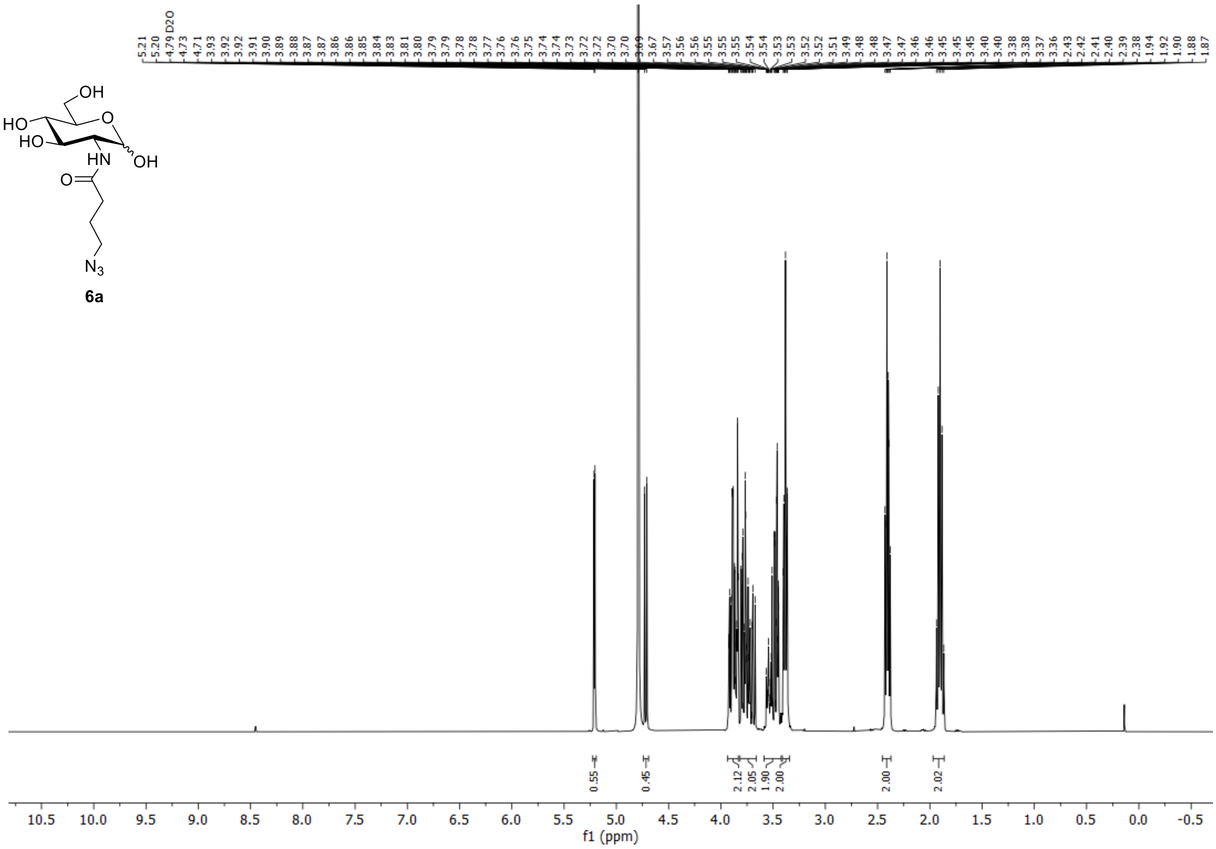

6a

^{13}C NMR, D_2O, 400 MHz

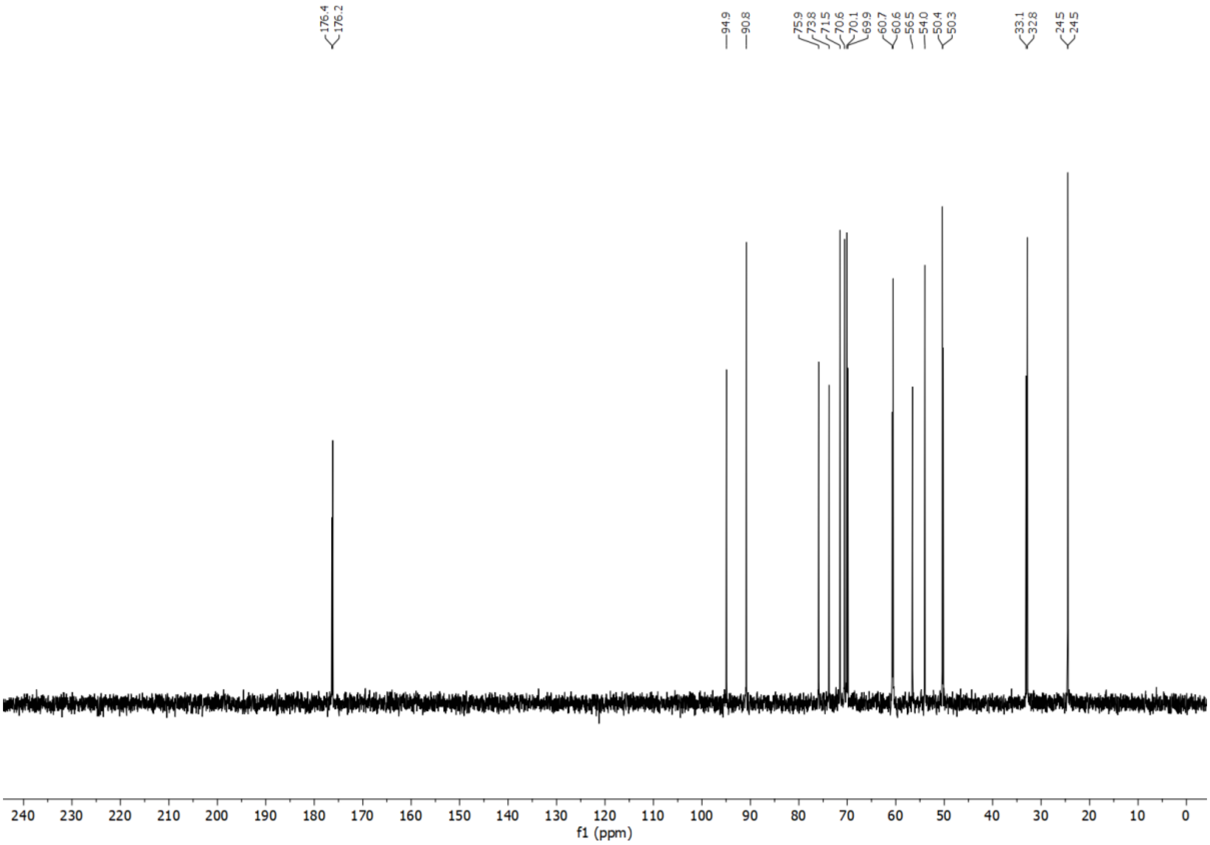

^{1}H NMR, $CDCl_3$ with TMS, 400 MHz

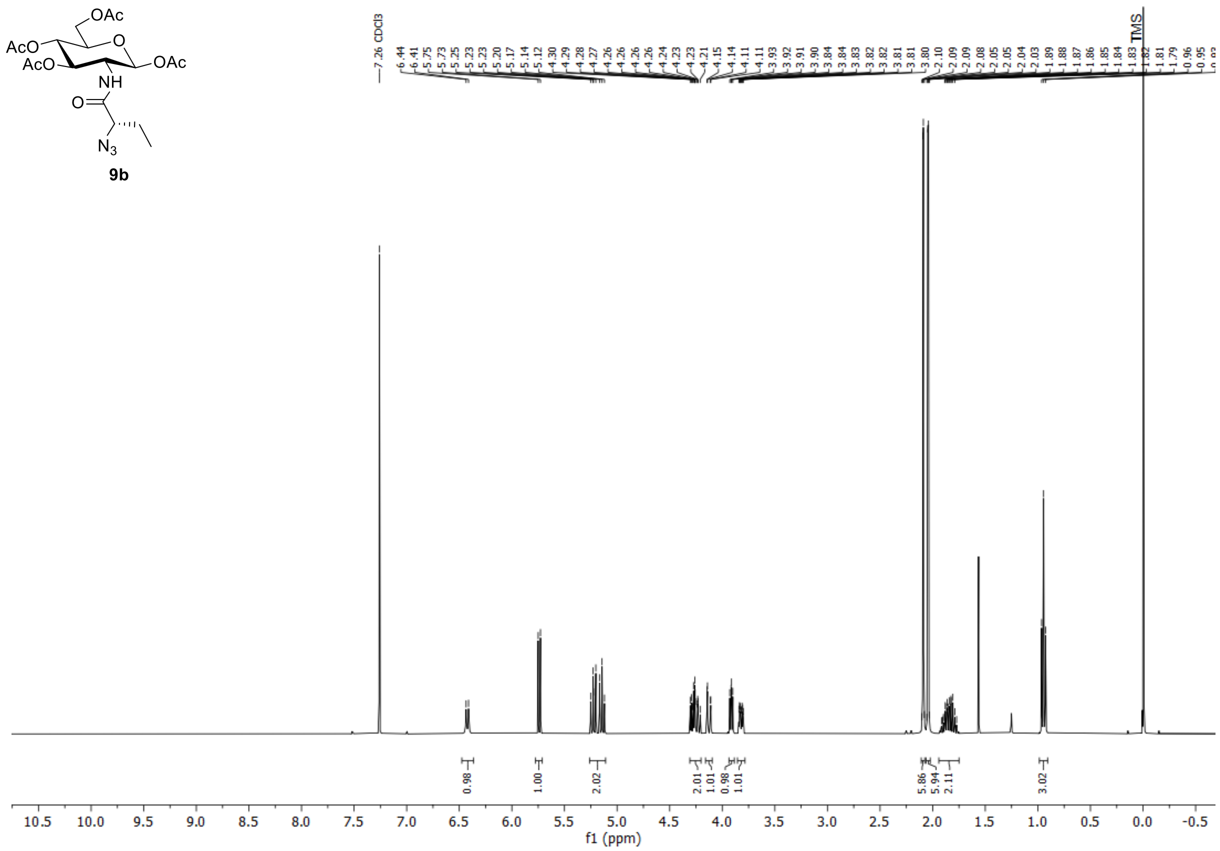

^{13}C NMR, $CDCl_3$ with TMS, 400 MHz

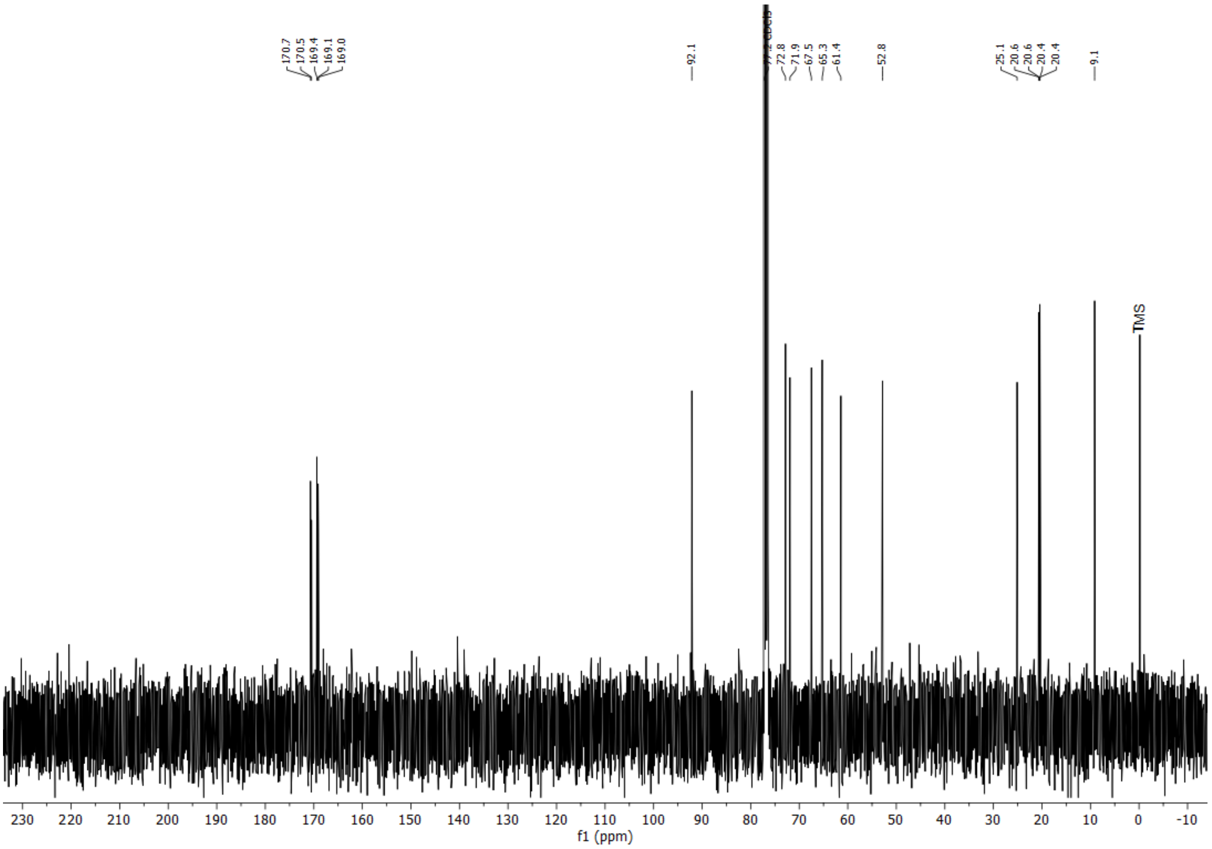

^{1}H NMR, CD_3OD, 400 MHz

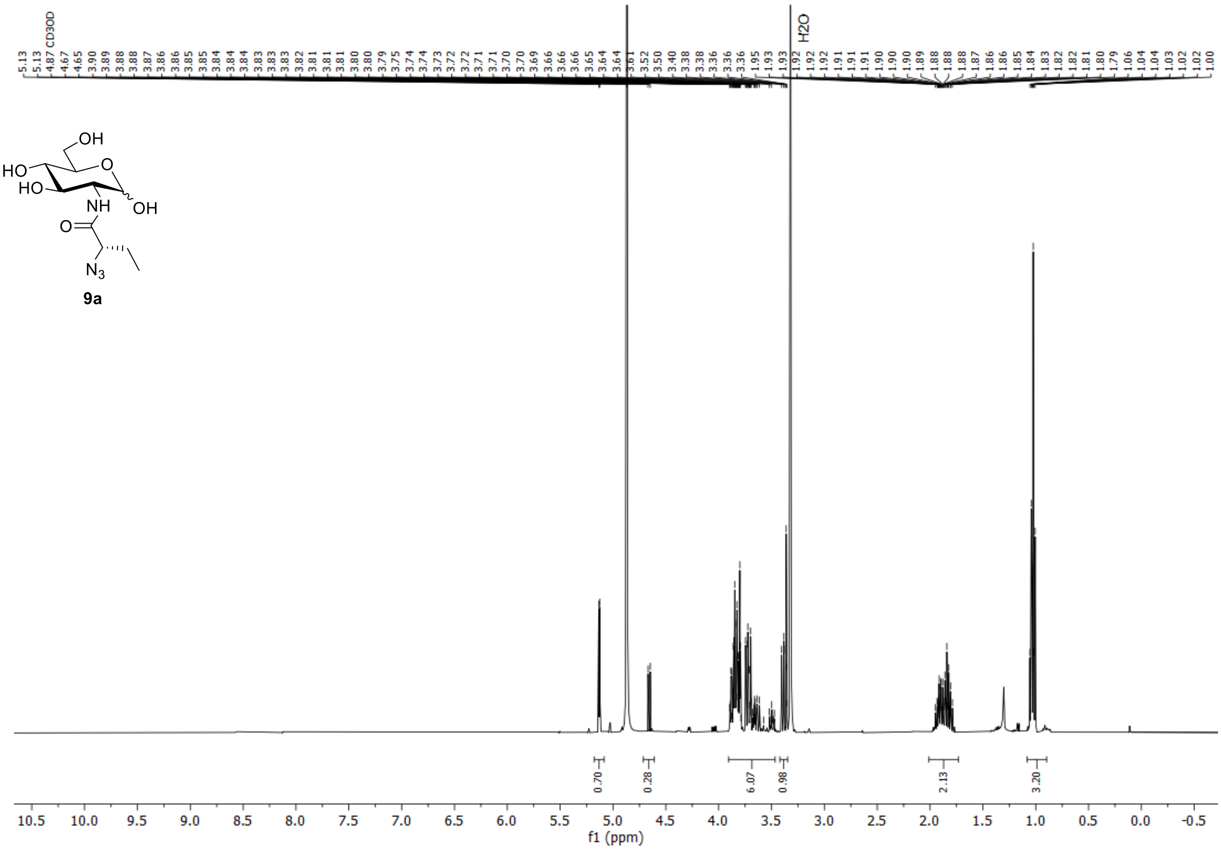

^{13}C NMR, CD_3OD, 400 MHz

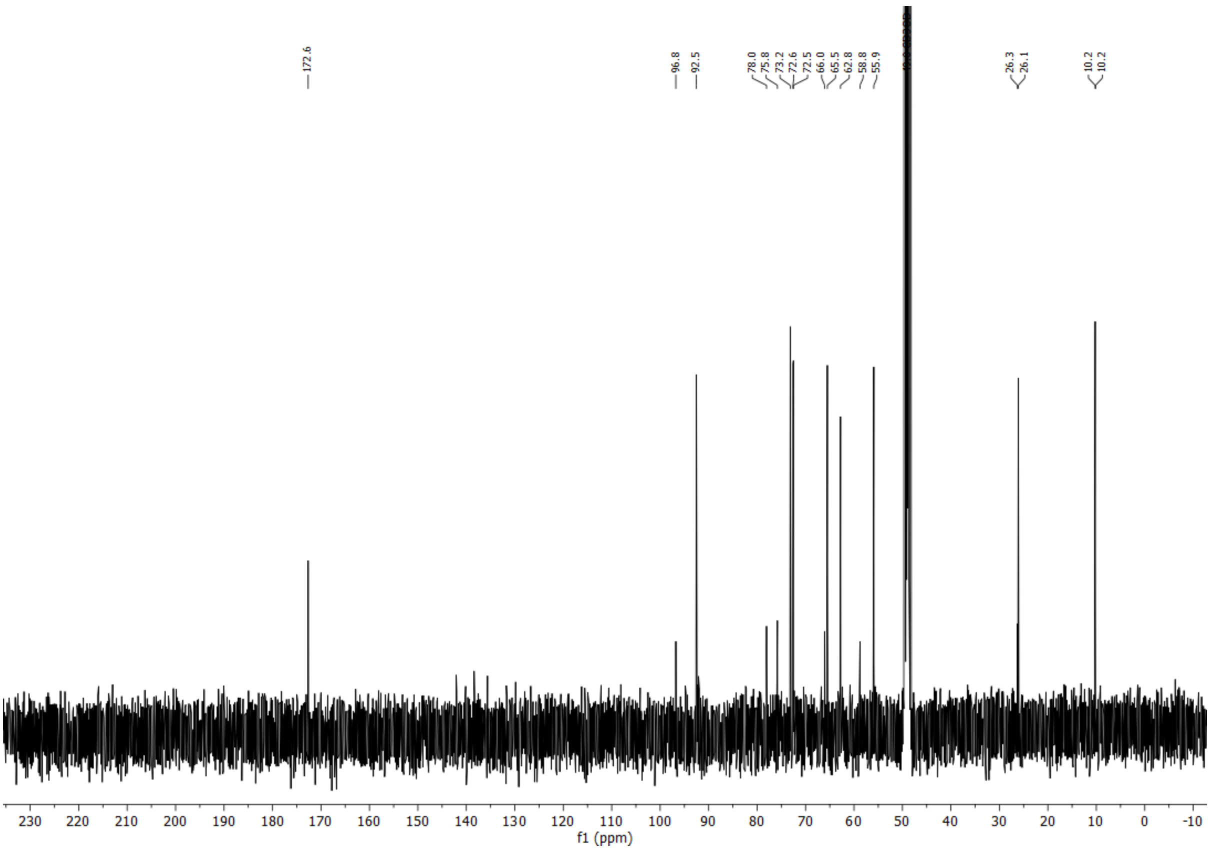

^{1}H NMR, D_2O, 400 MHz

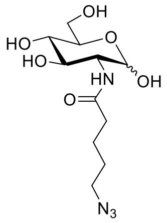

10a

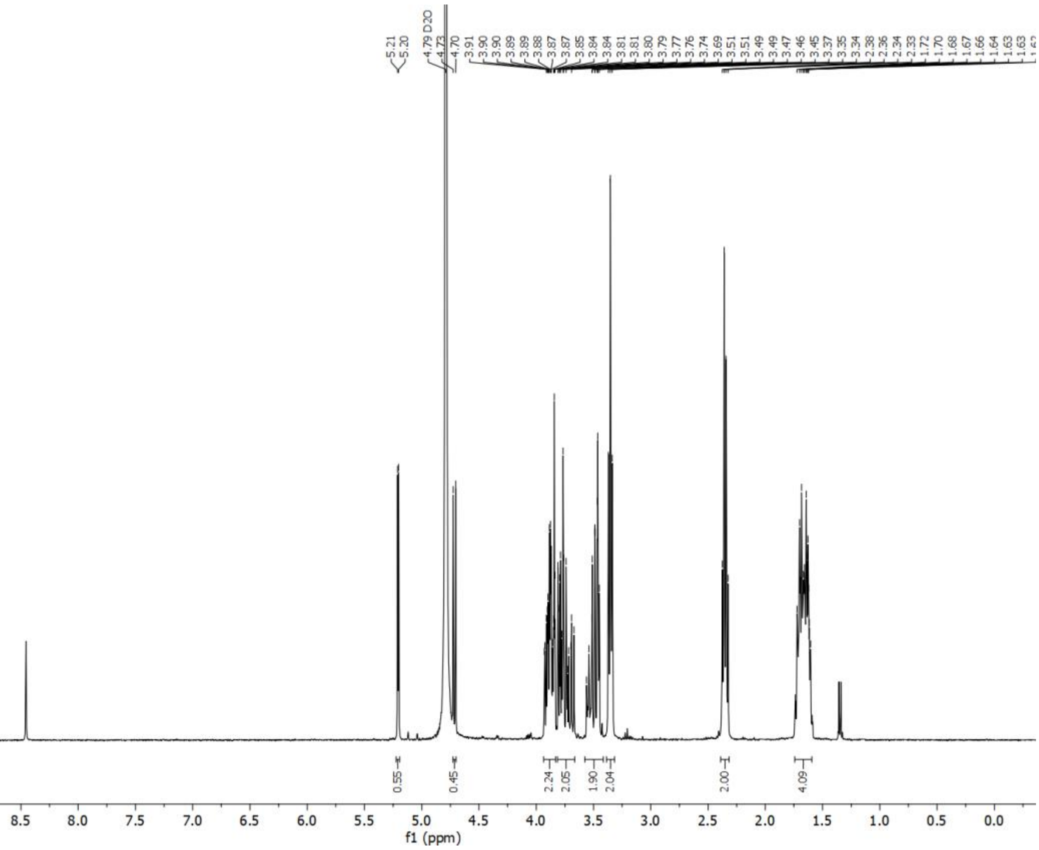

^{13}C NMR, D_2O, 400 MHz

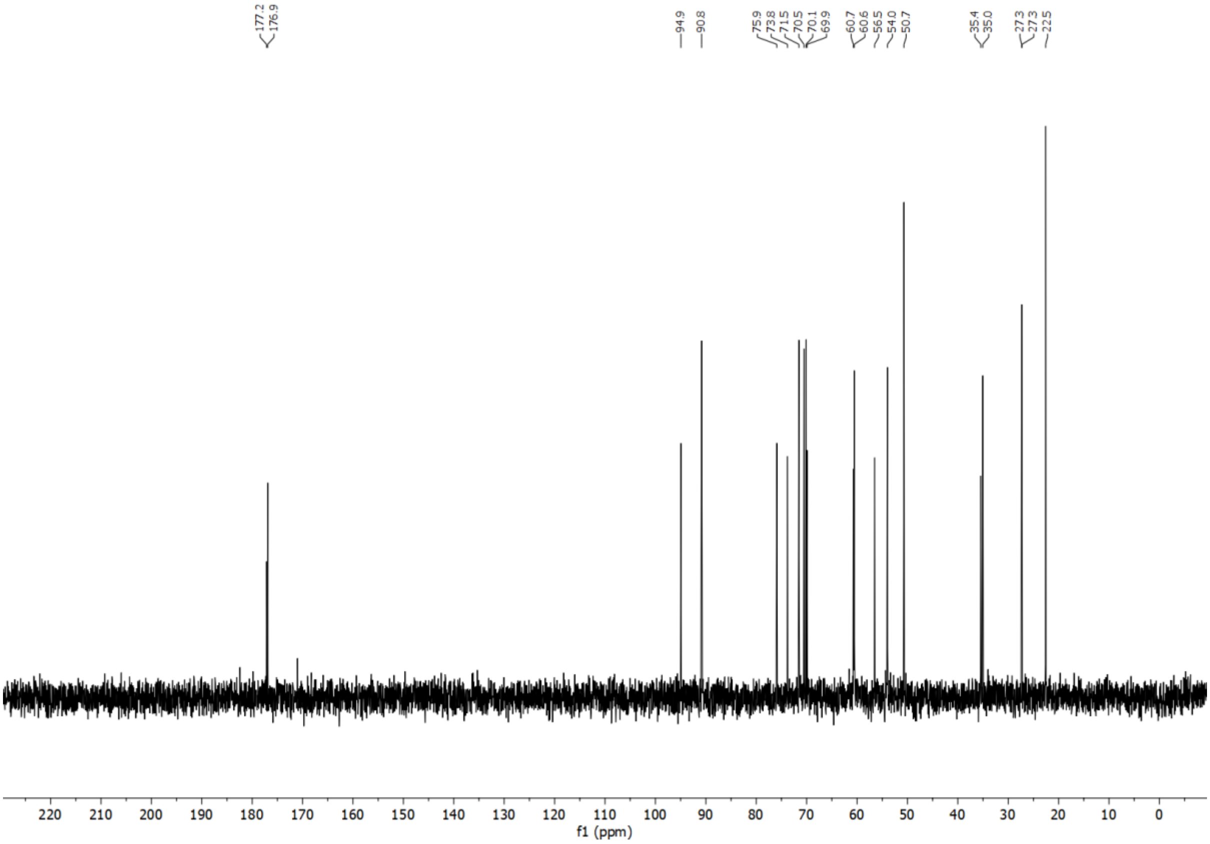

^{1}H NMR, $CDCl_3$, 400 MHz

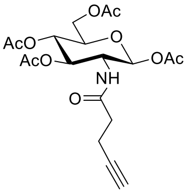

SI-2

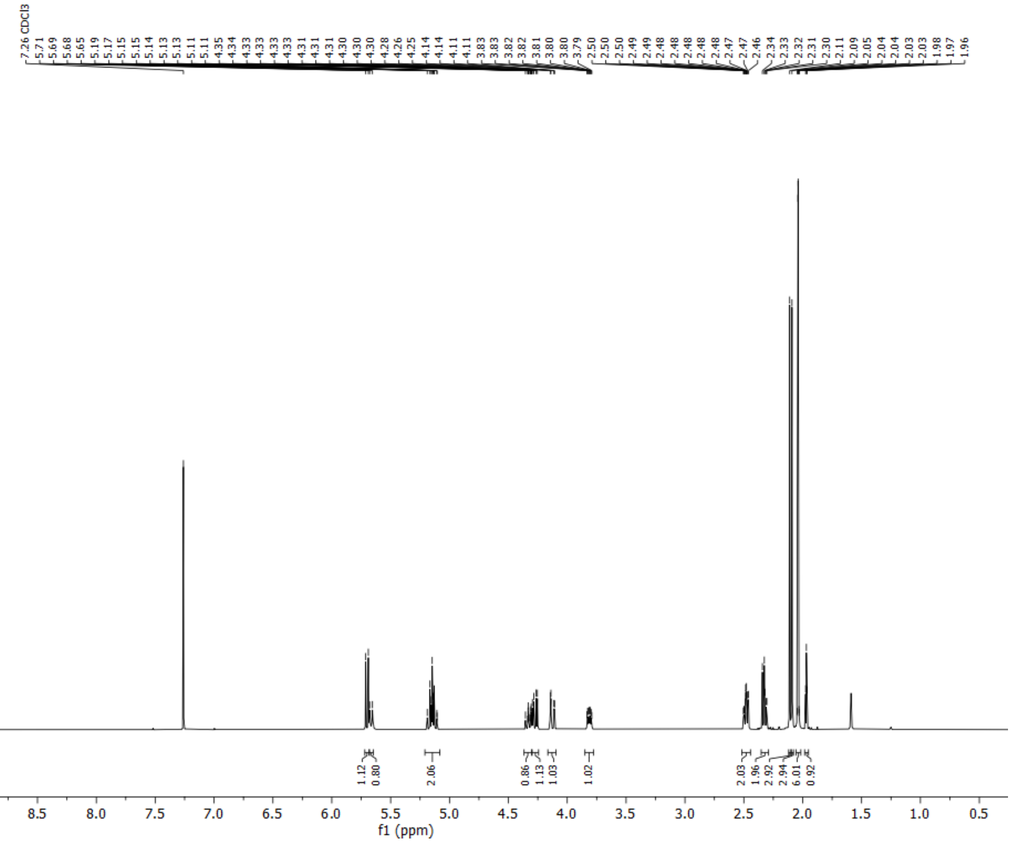

^{13}C NMR, $CDCl_3$, 400 MHz

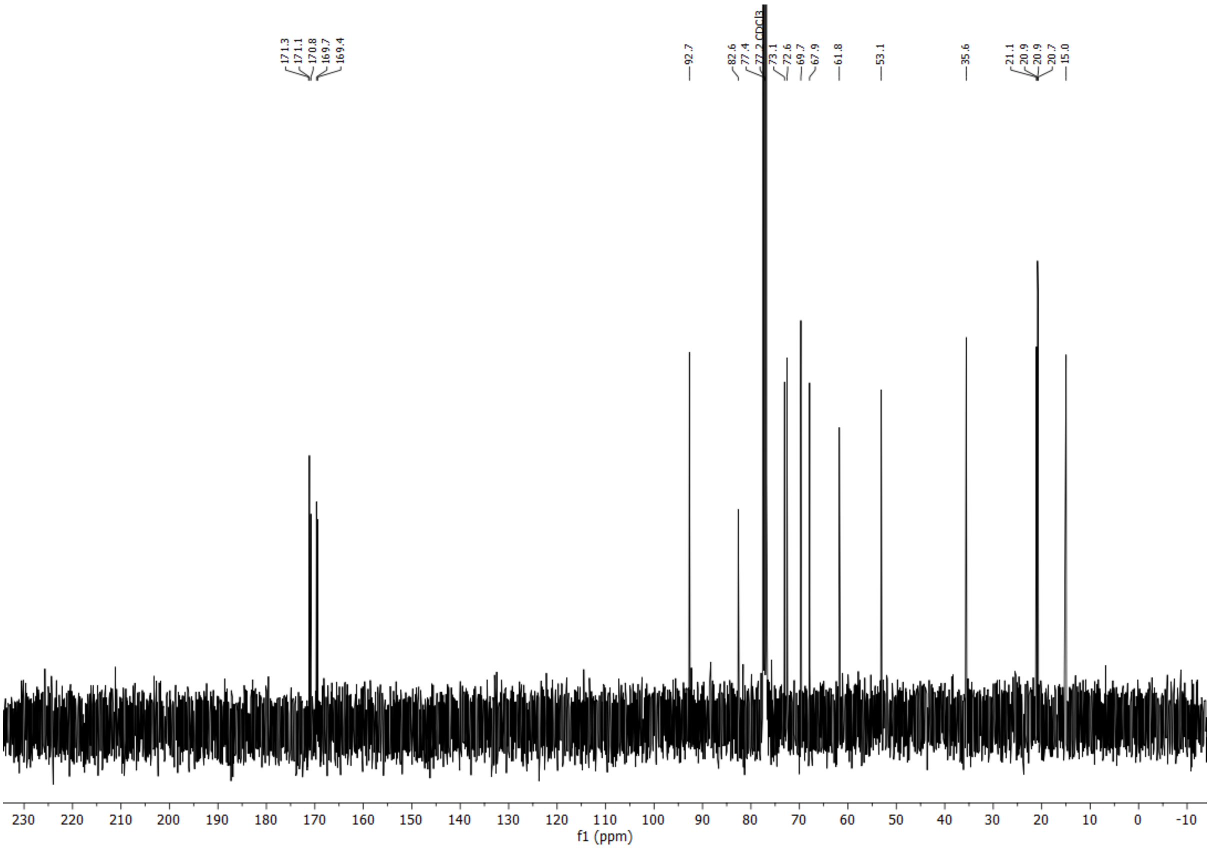

^{1}H NMR, $CDCl_3$, 400 MHz

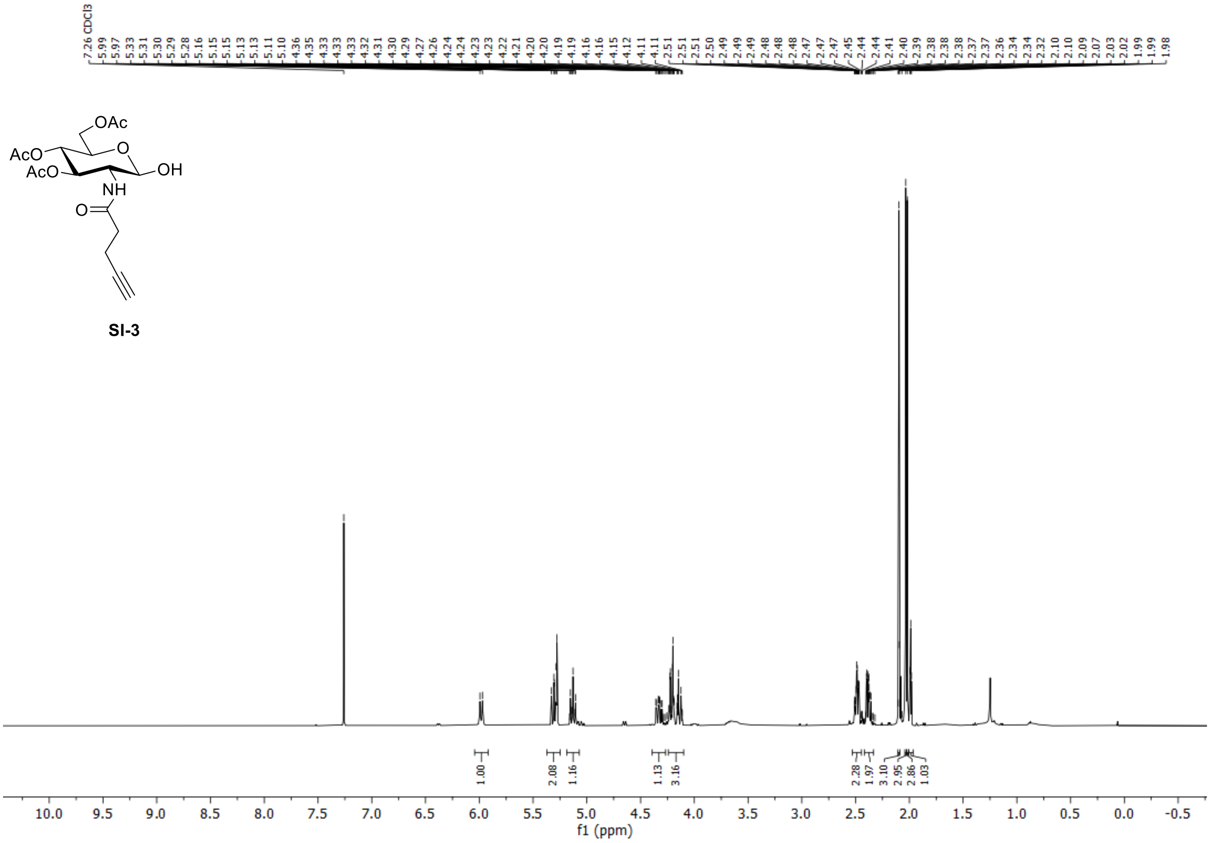

^{13}C NMR, $CDCl_3$, 400 MHz

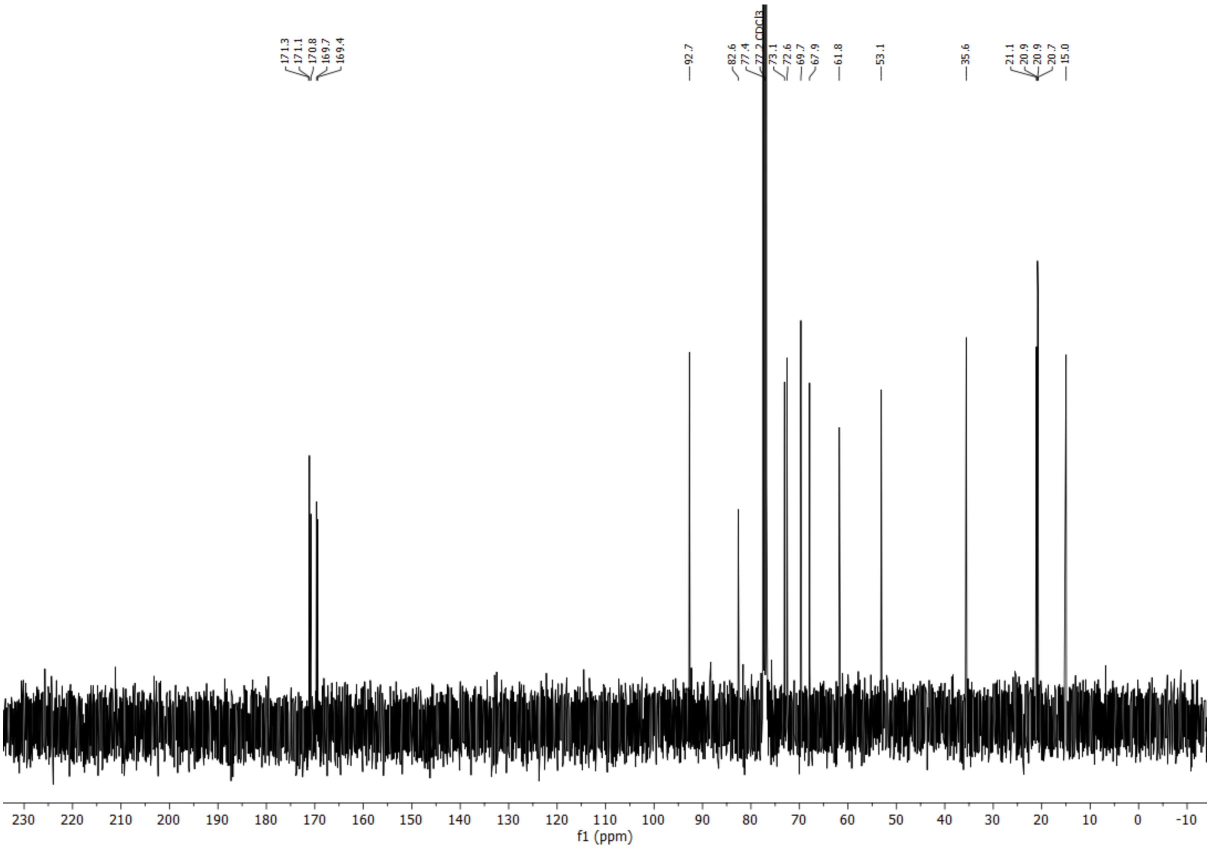

^{1}H NMR, $CDCl_3$, 400 MHz

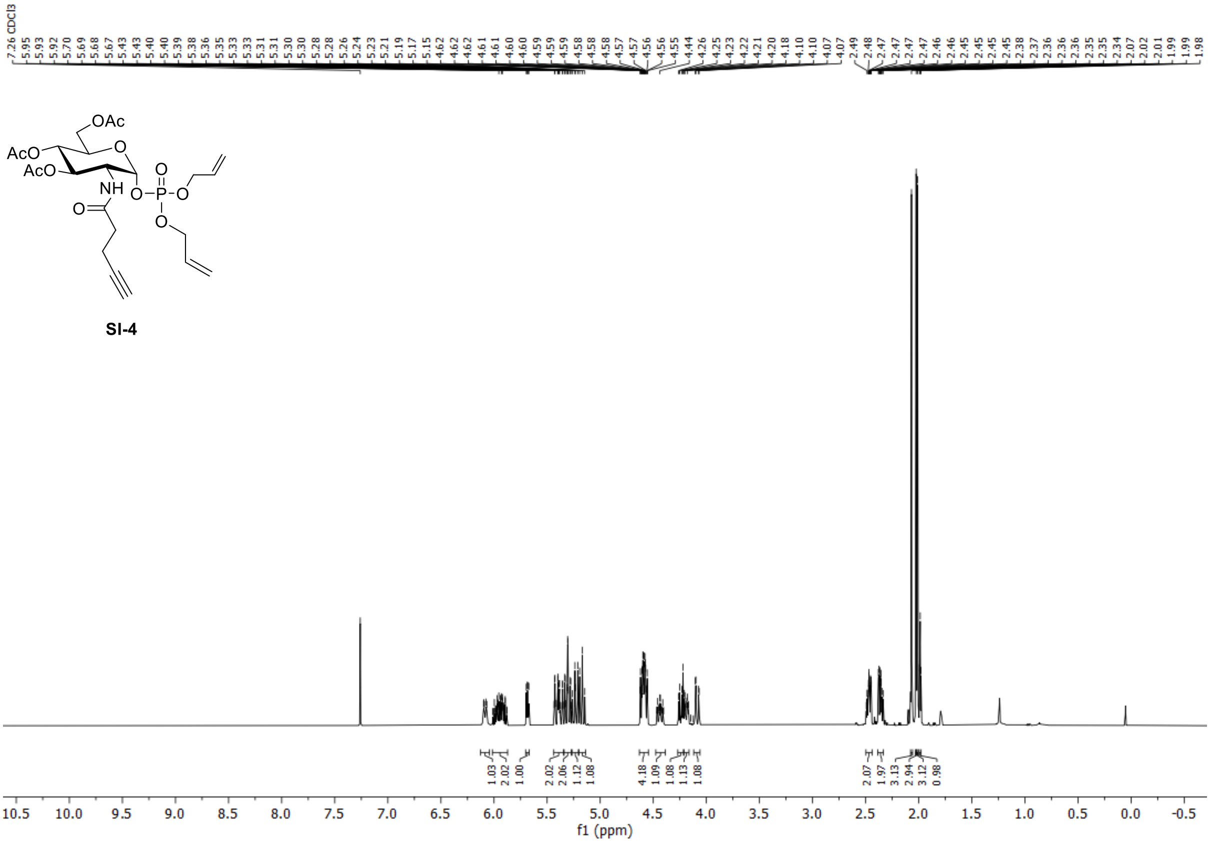

^{13}C NMR, $CDCl_3$, 400 MHz

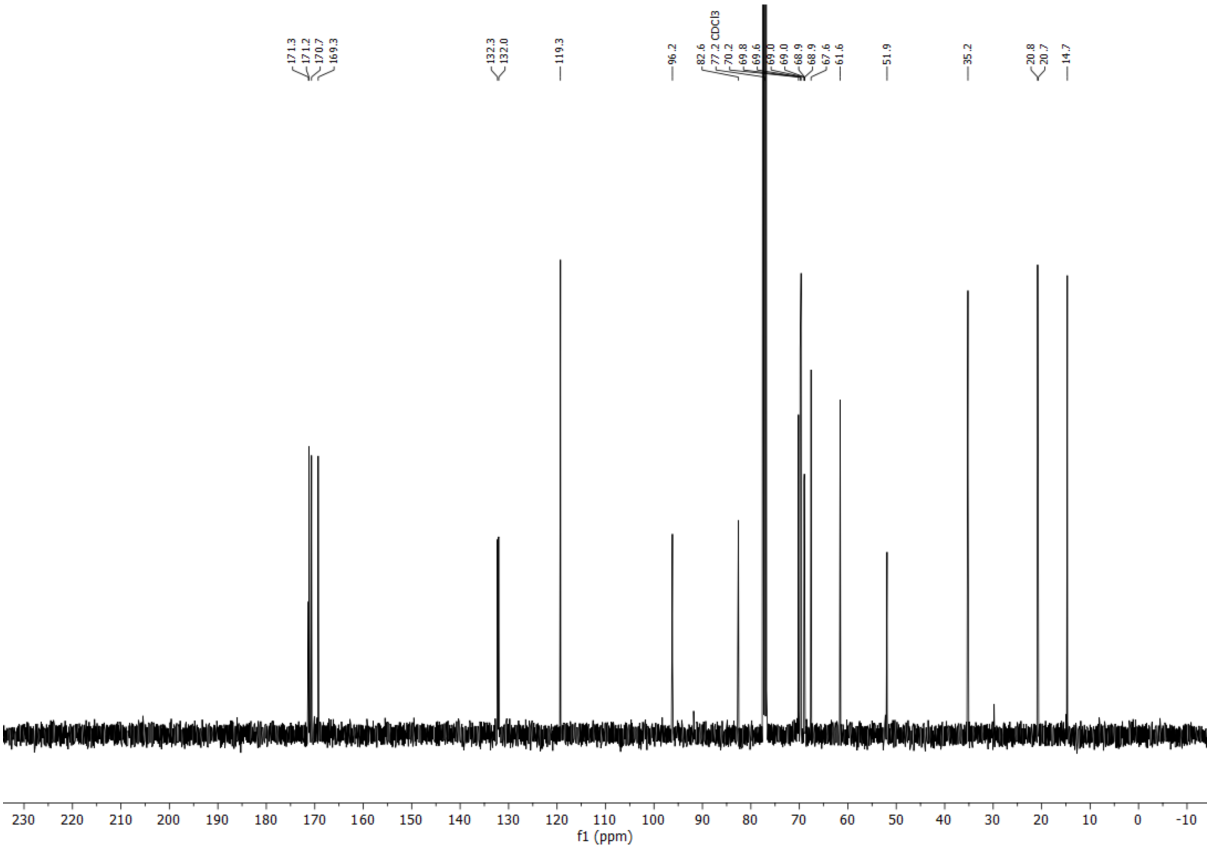

^{1}H NMR, CD_3OD, 400 MHz

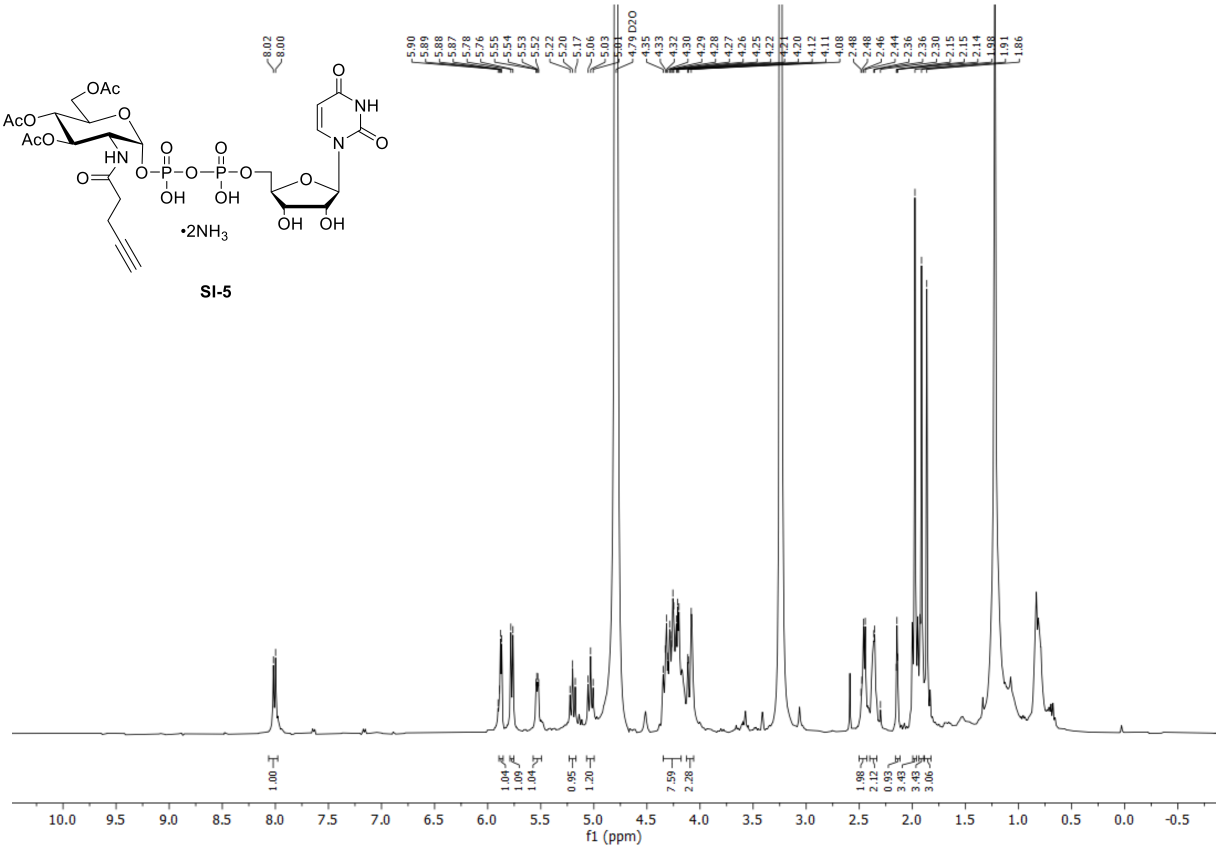

SI-5

^{13}C NMR, CD_3OD, 400 MHz

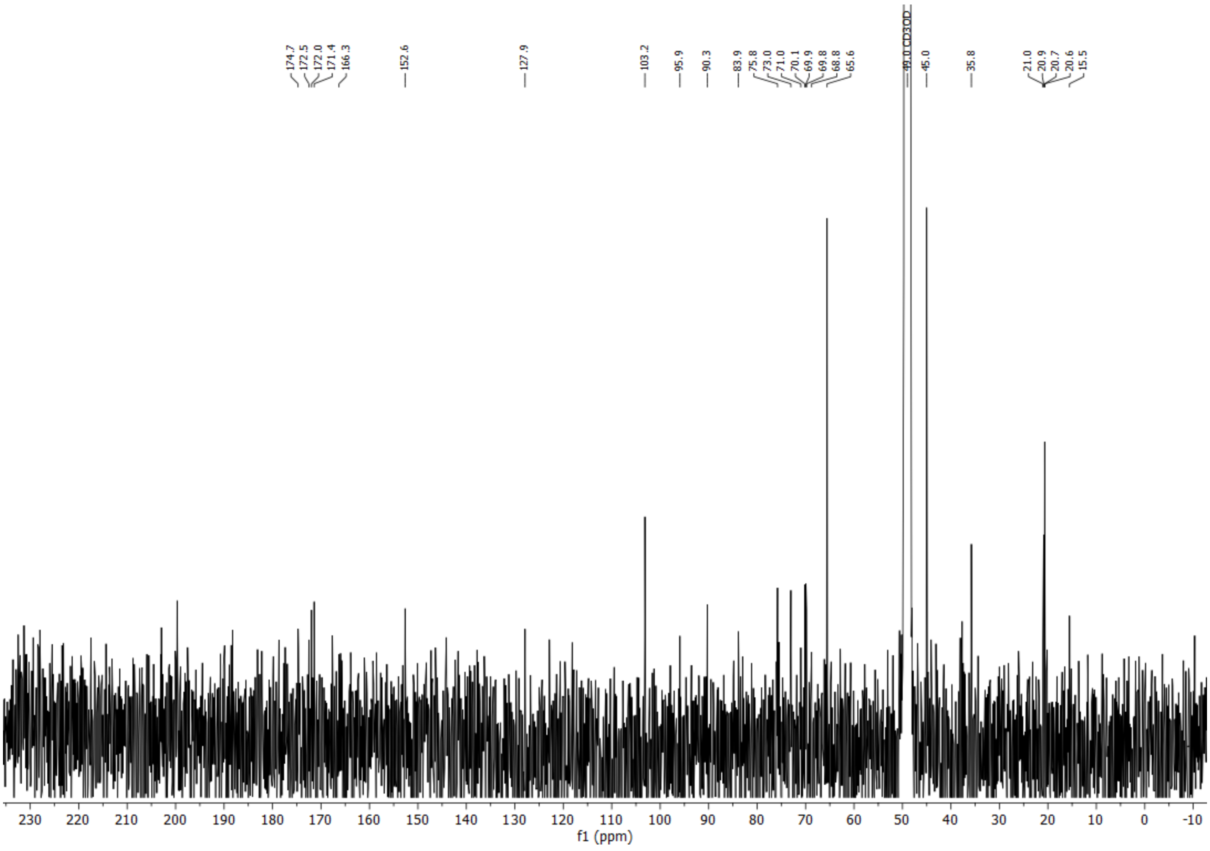

^{1}H NMR, D_2O, 600 MHz

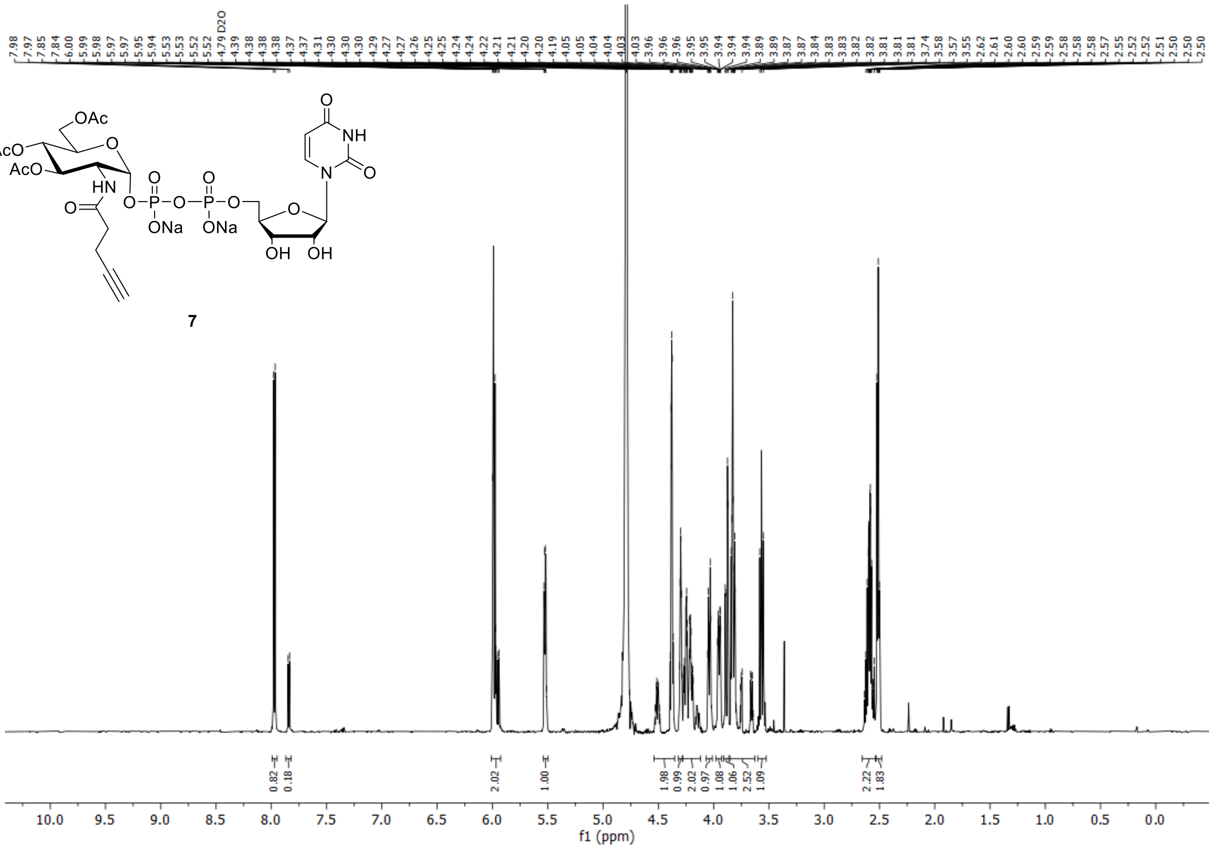

^{13}C NMR, D_2O, 600 MHz

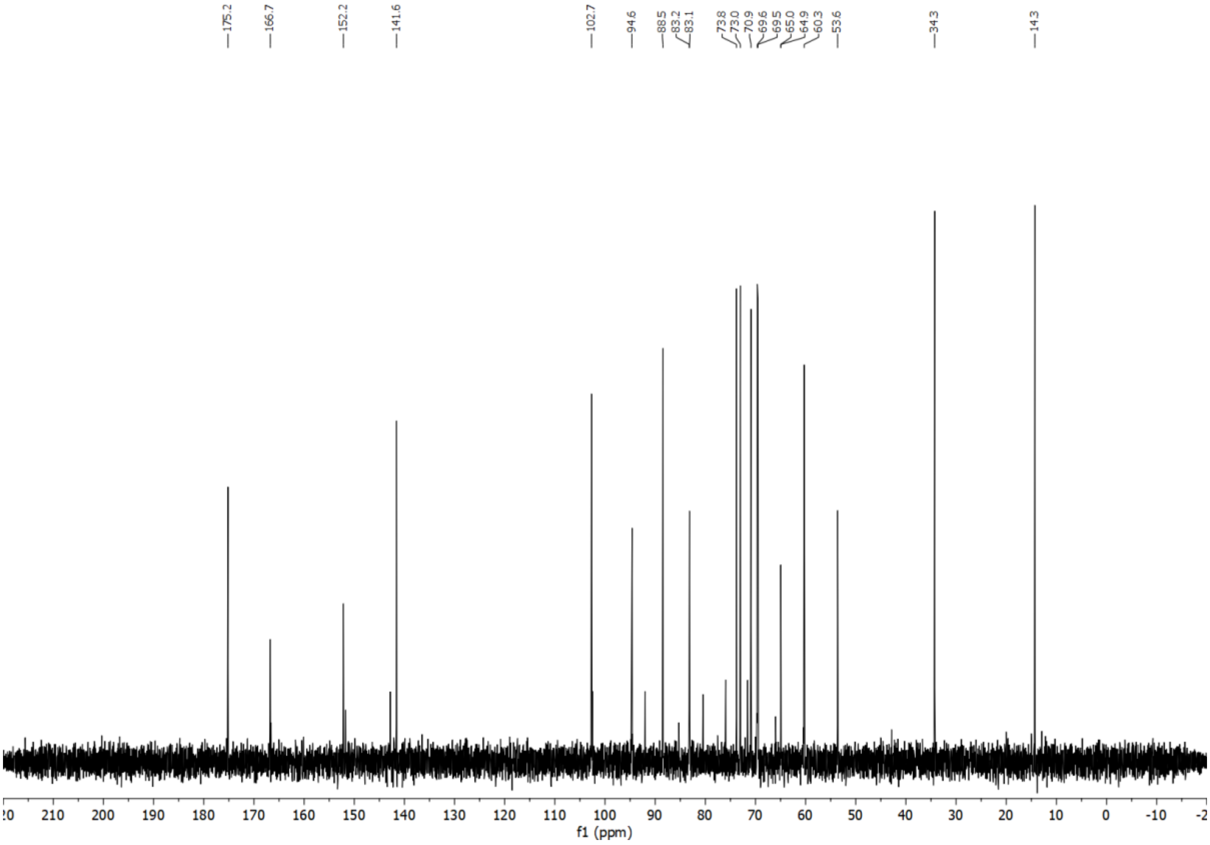

^{31}P NMR, D_2O, 400 MHz

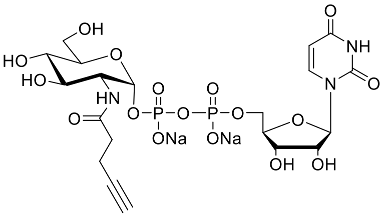

7

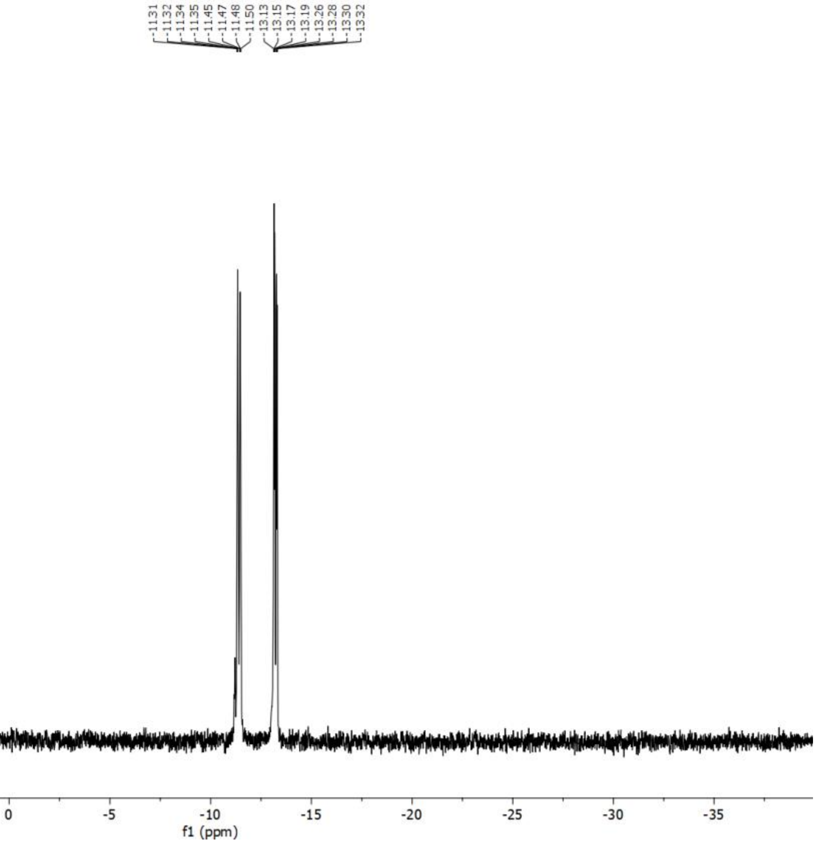

VT ^1H NMR, D_2O, 600 MHz

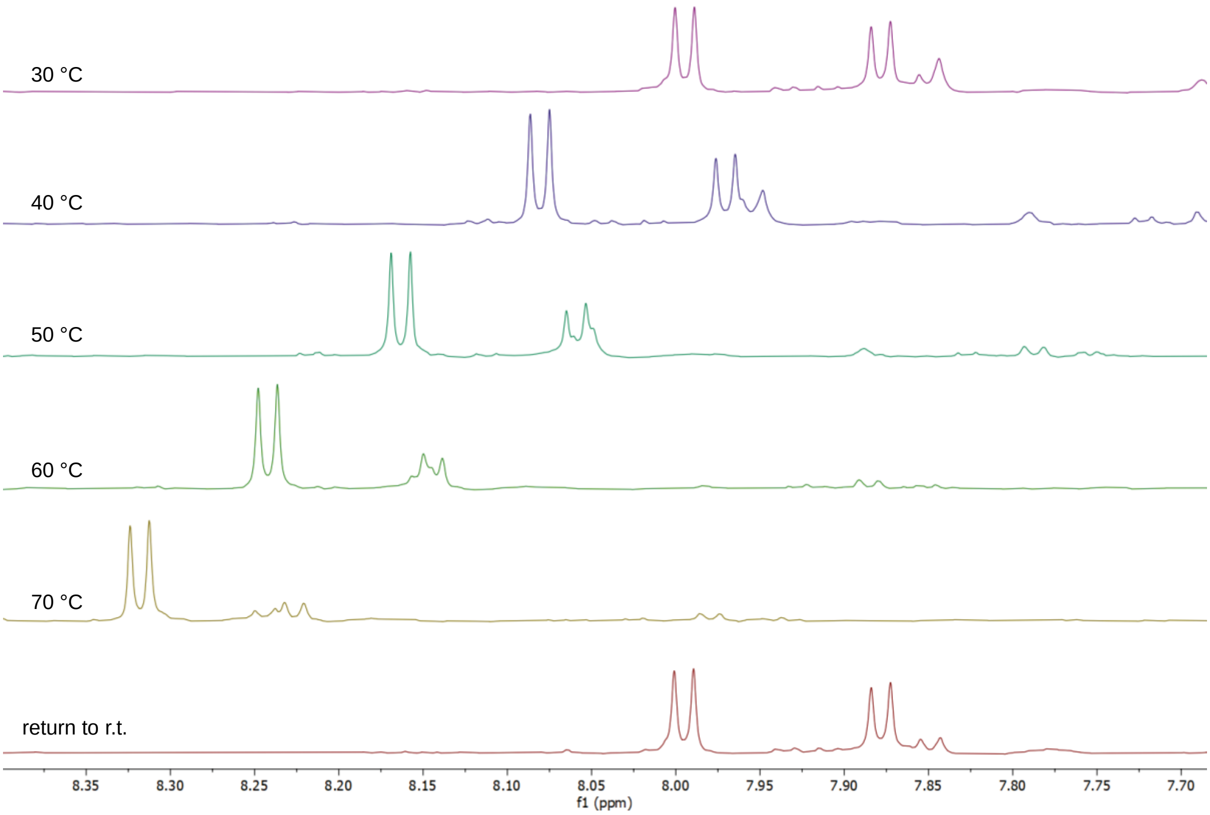

^{1}H NMR, $CDCl_3$, 400 MHz

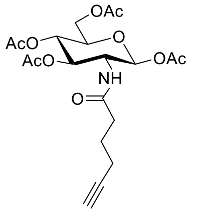

SI-6

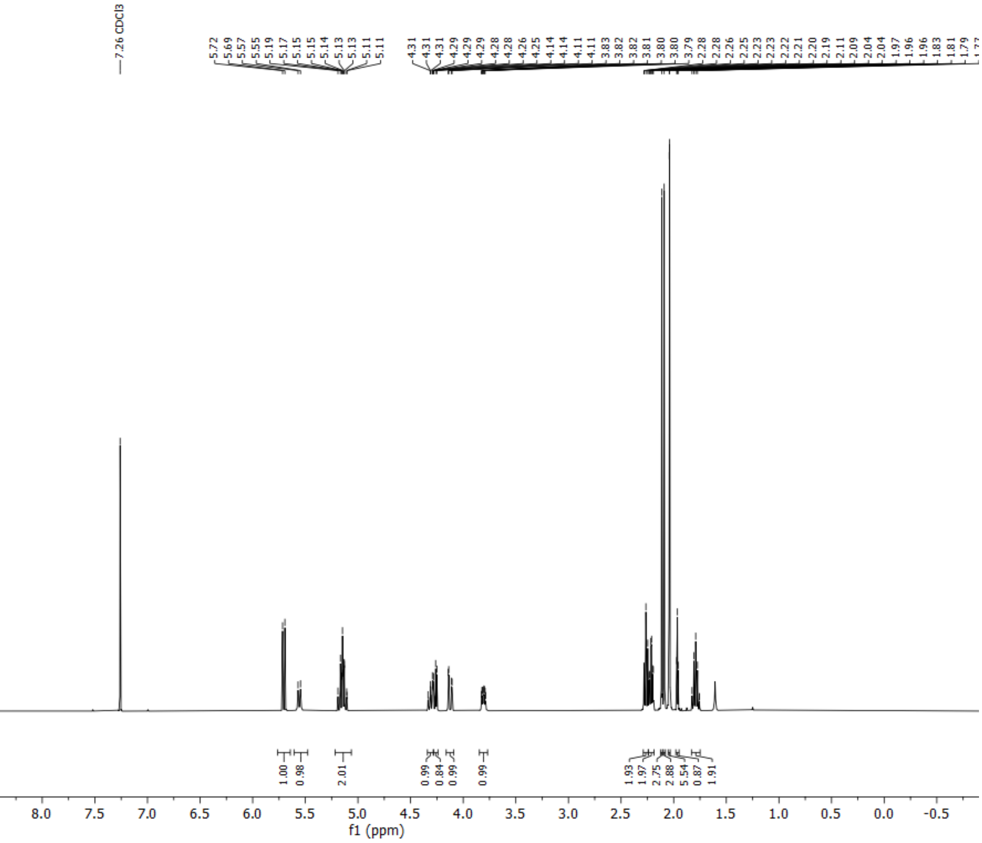

^{13}C NMR, $CDCl_3$, 400 MHz

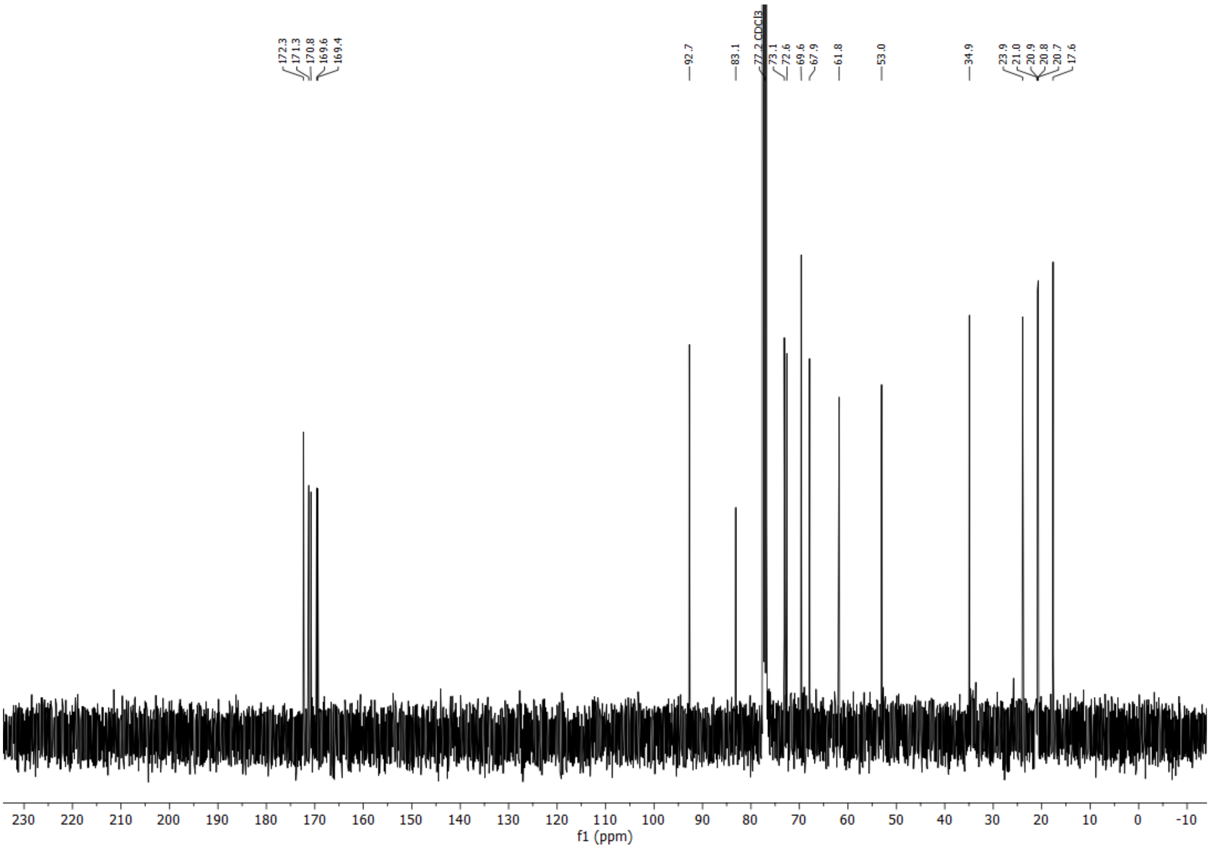

^{1}H NMR, $CDCl_3$, 400 MHz

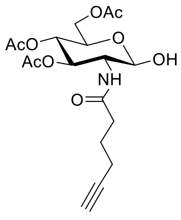

SI-7

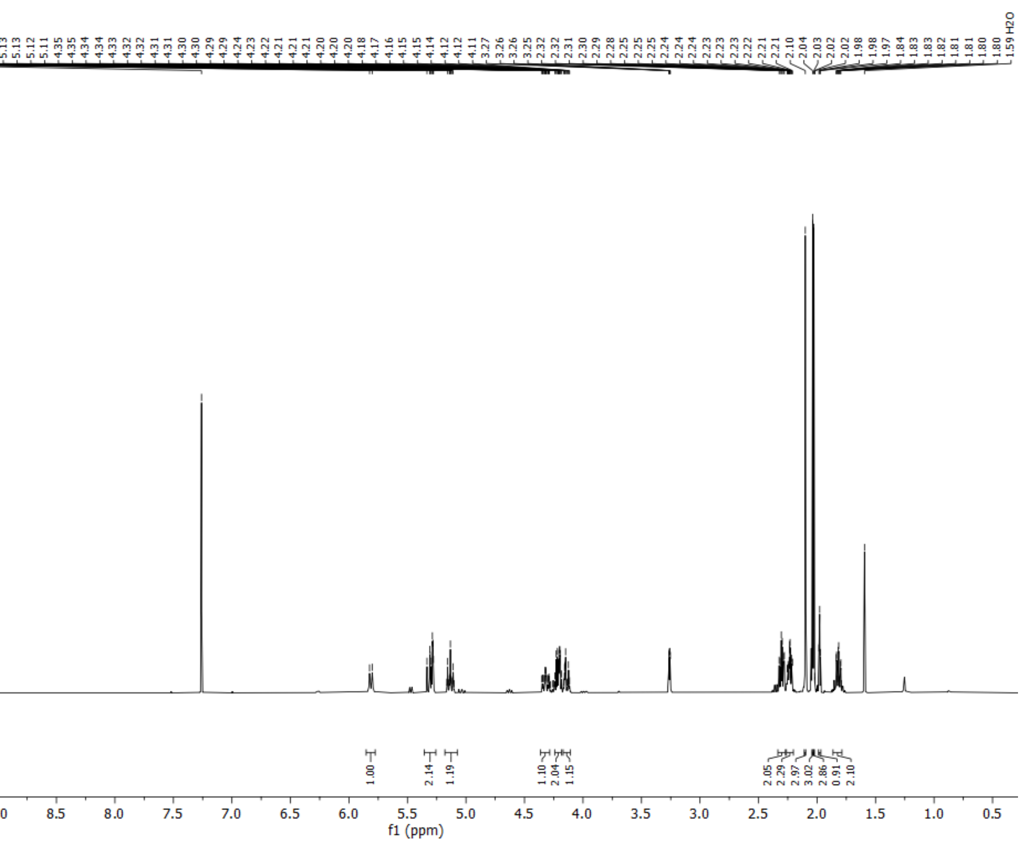

^{13}C NMR, $CDCl_3$, 400 MHz

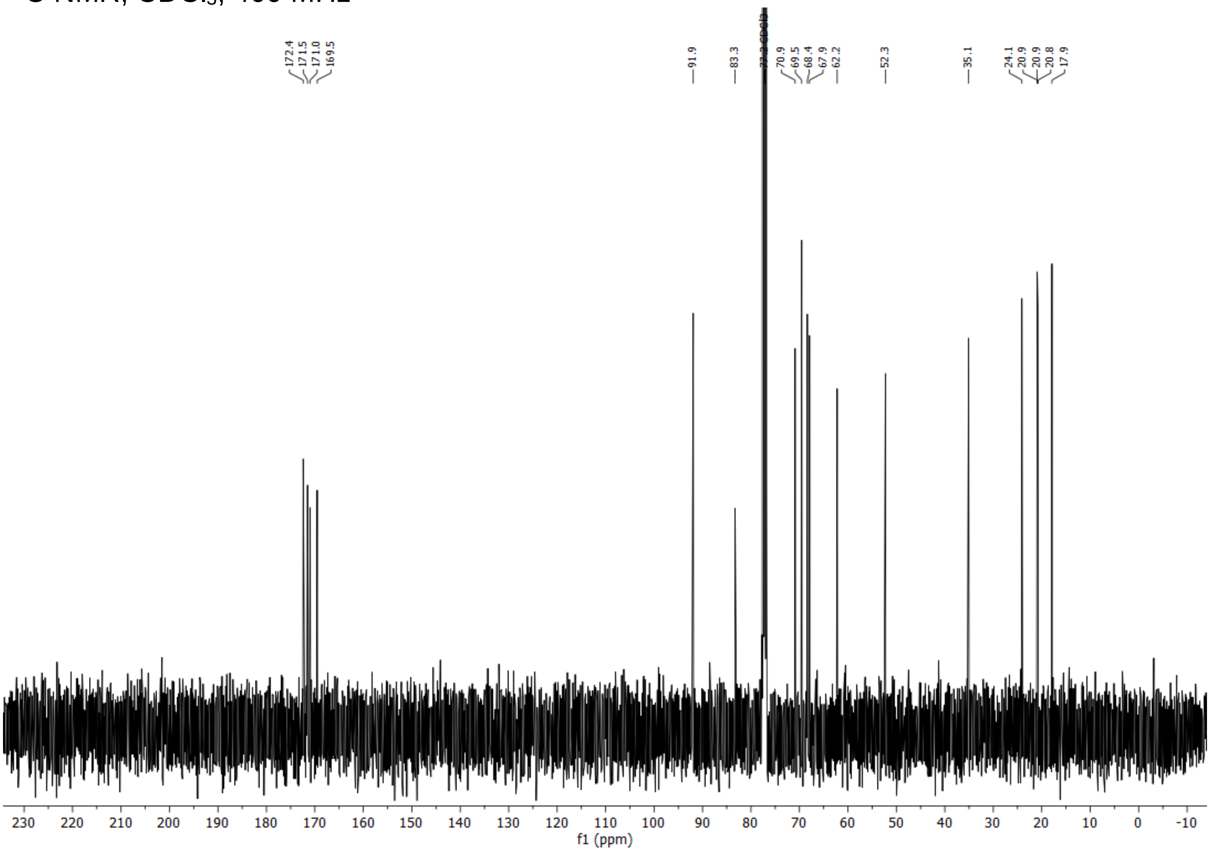

^{1}H NMR, CDCl3, 400 MHz

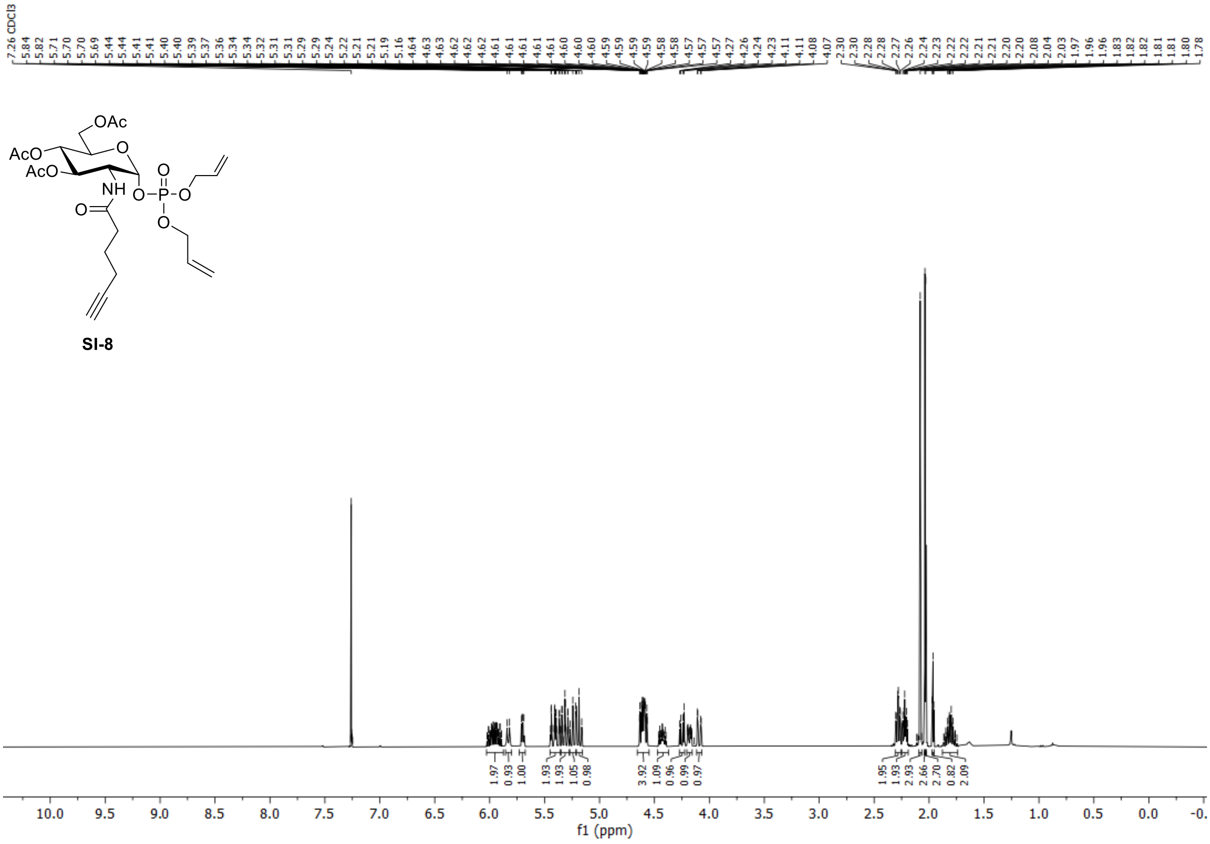

^{13}C NMR, CDCl3, 400 MHz

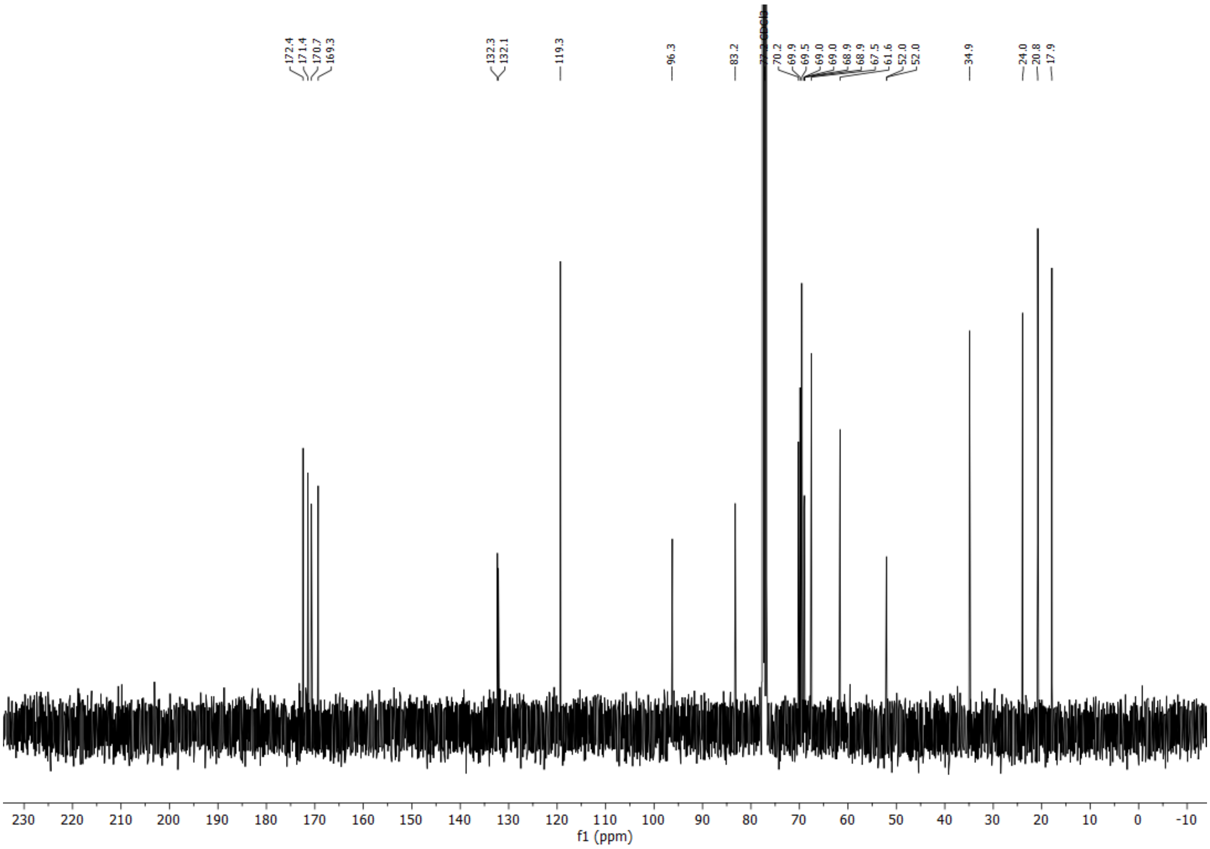

^{1}H NMR, D_2O, 700 MHz

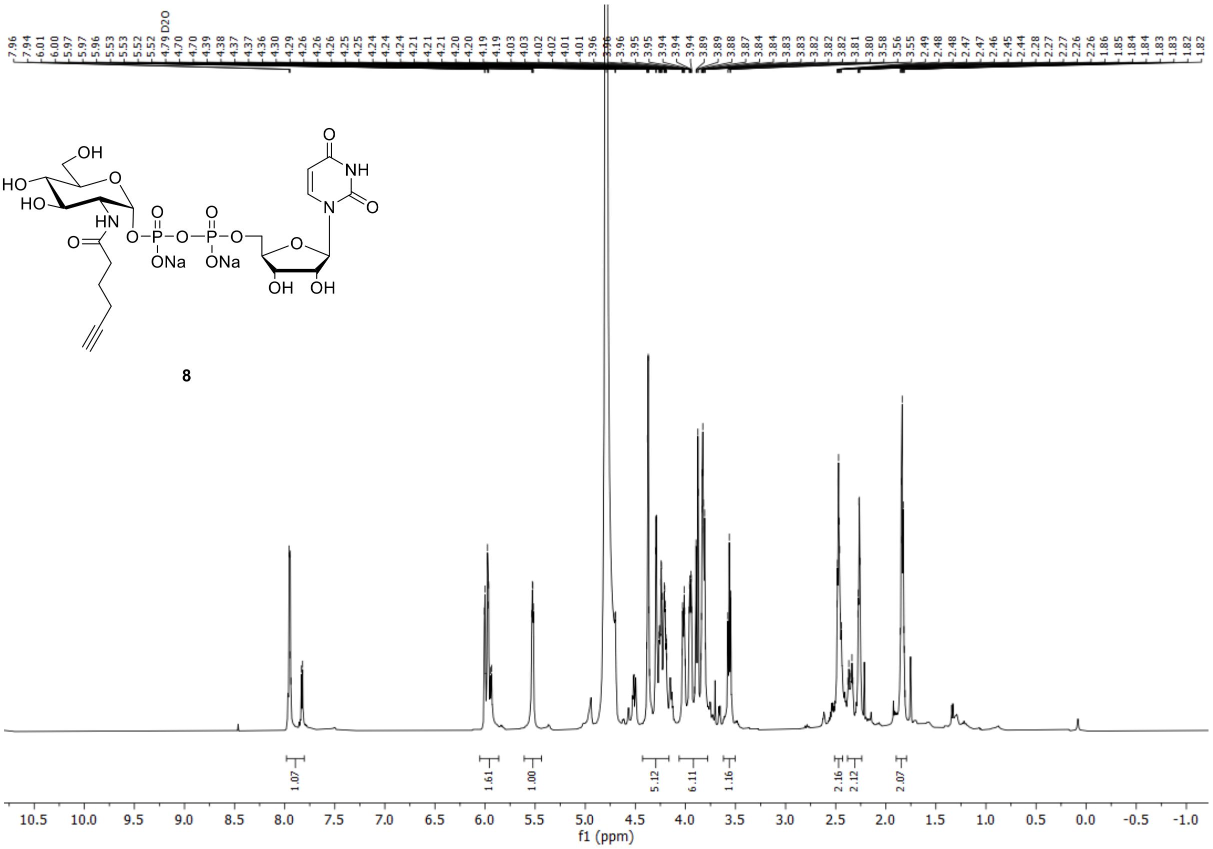

^{13}C NMR, D_2O, 700 MHz

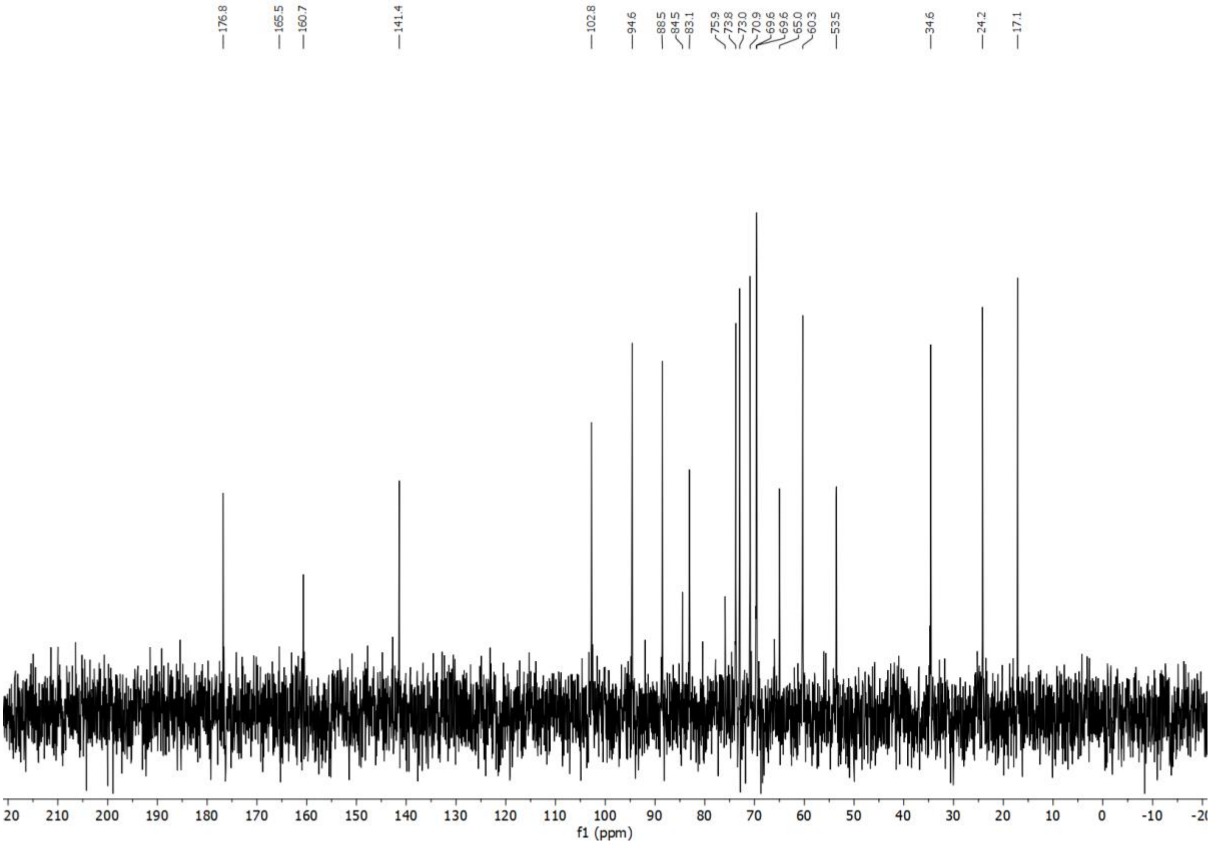

^{1}H NMR, D_2O, 400 MHz

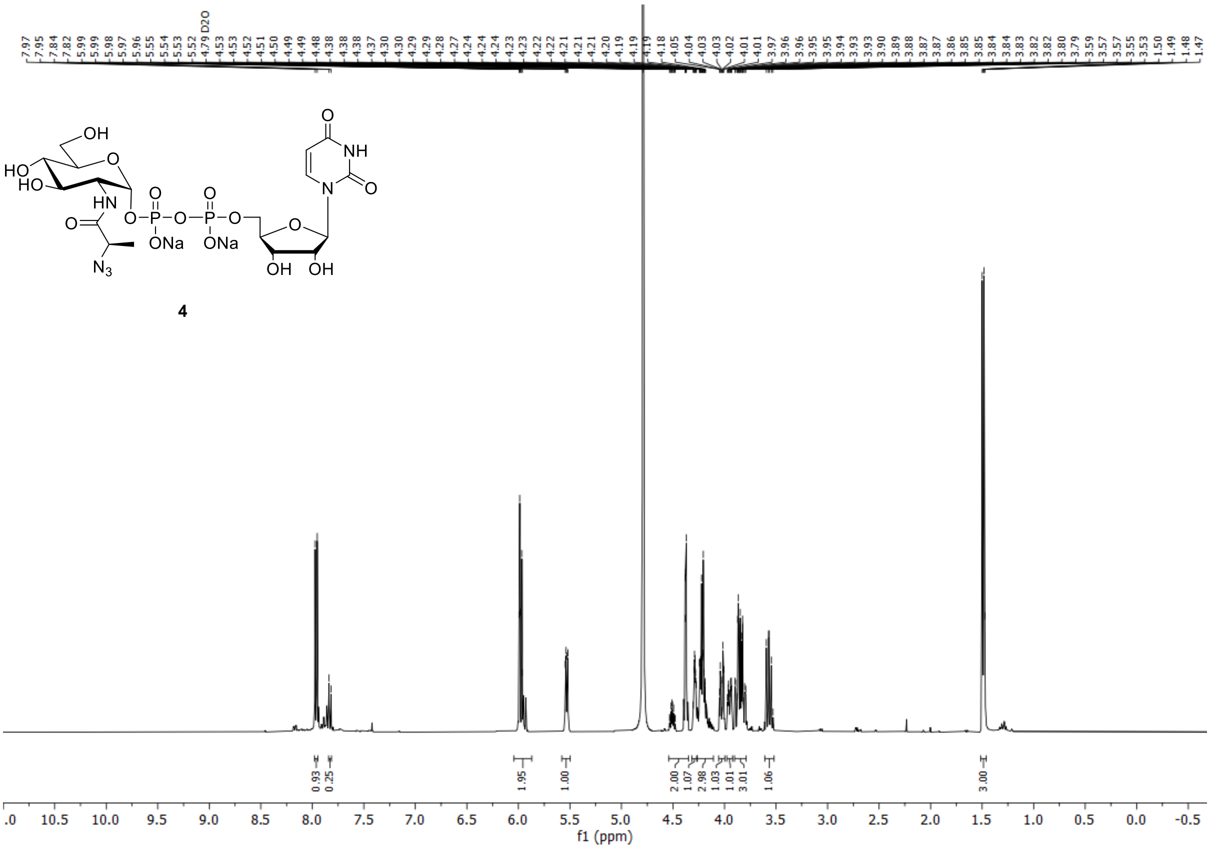

^{13}C NMR, D_2O, 700 MHz

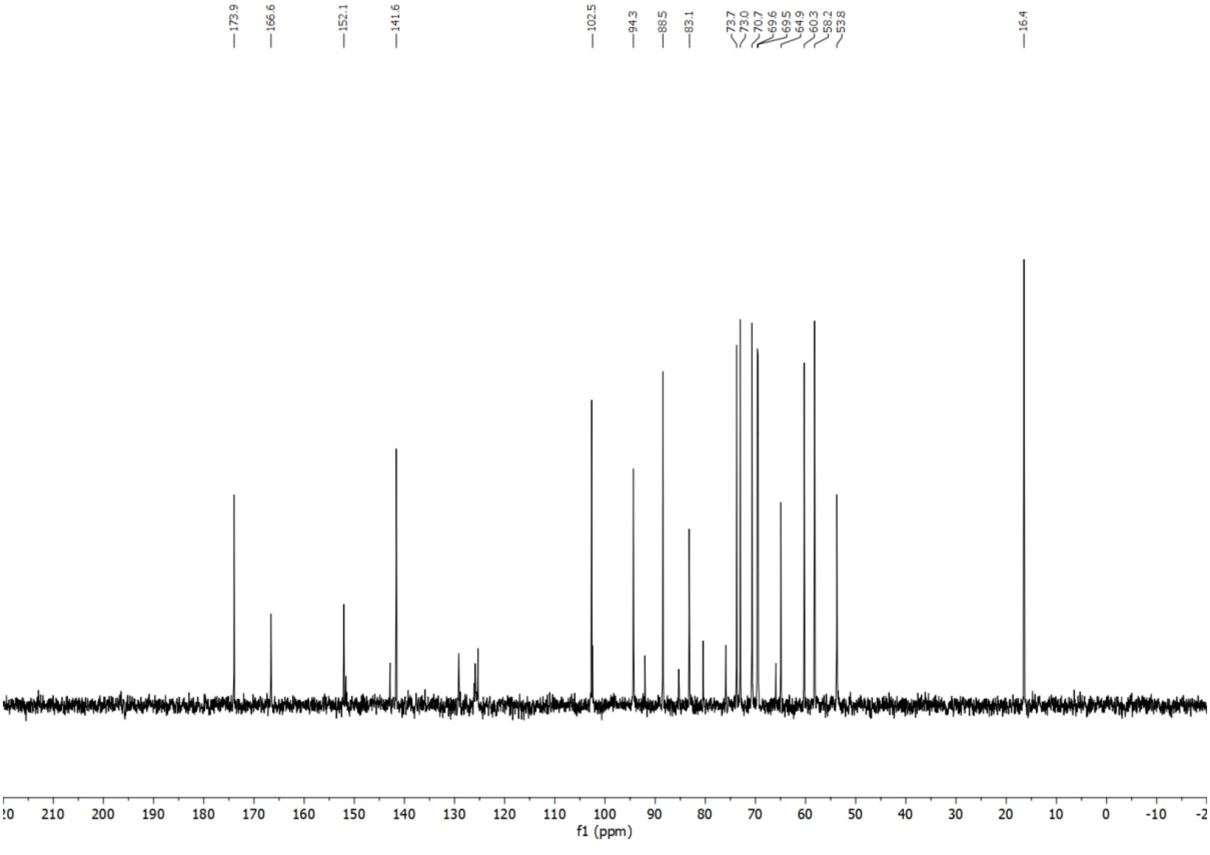

^{1}H NMR, D_2O, 400 MHz

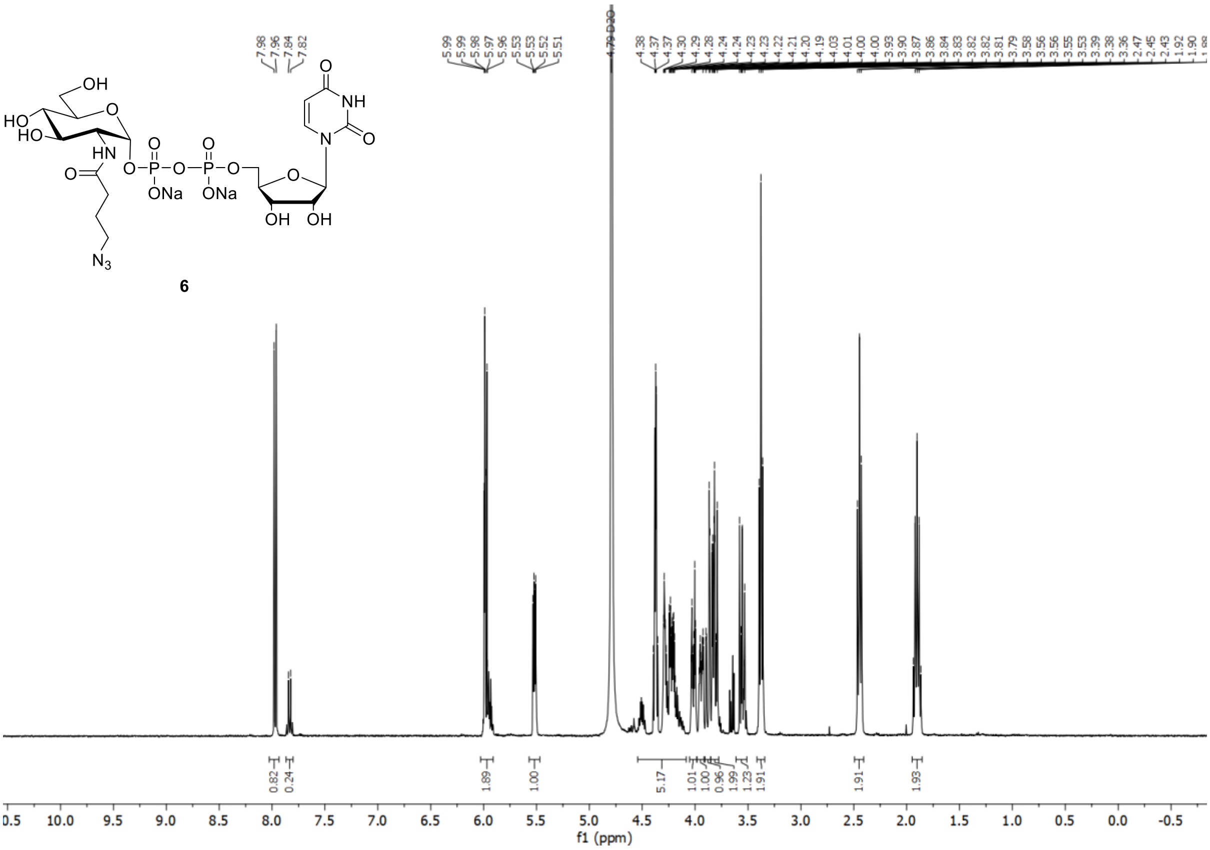

^{13}C NMR, D_2O, 400 MHz

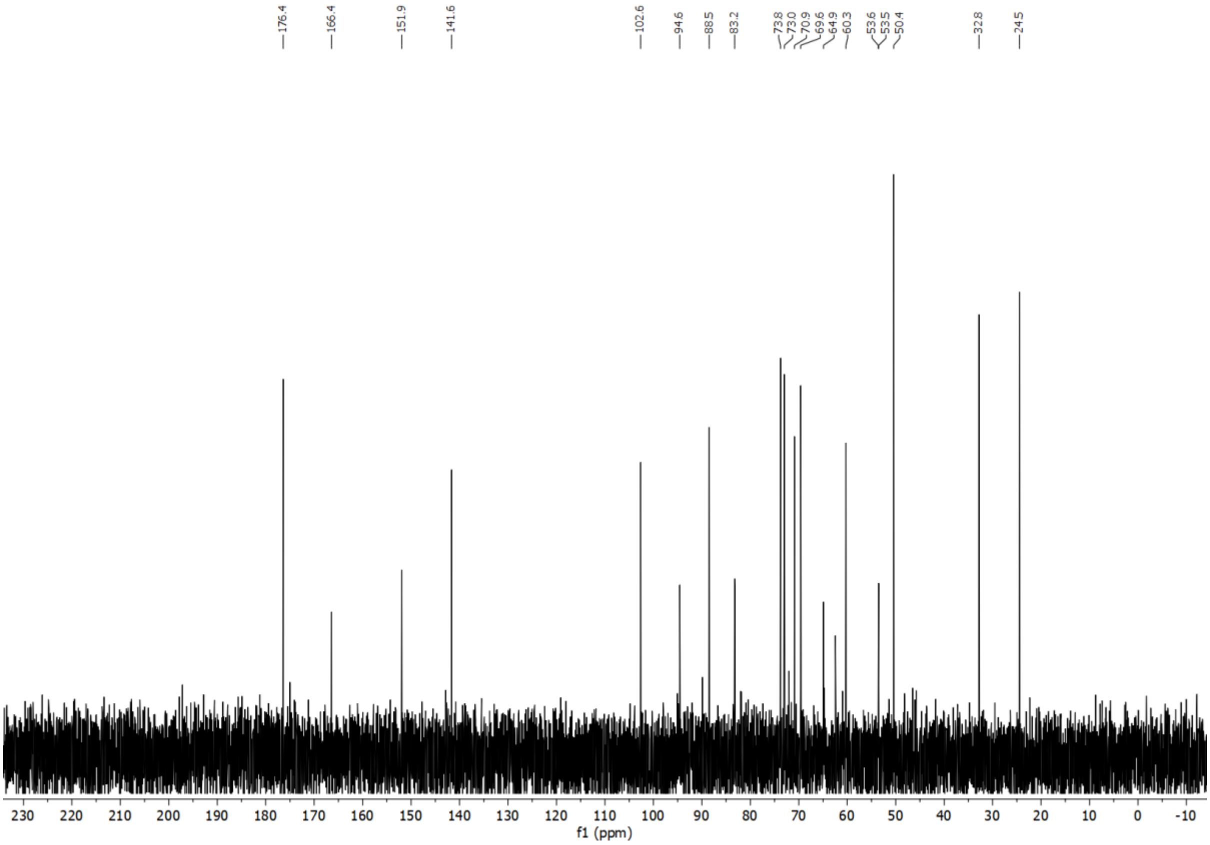

^{1}H NMR, D_2O, 400 MHz

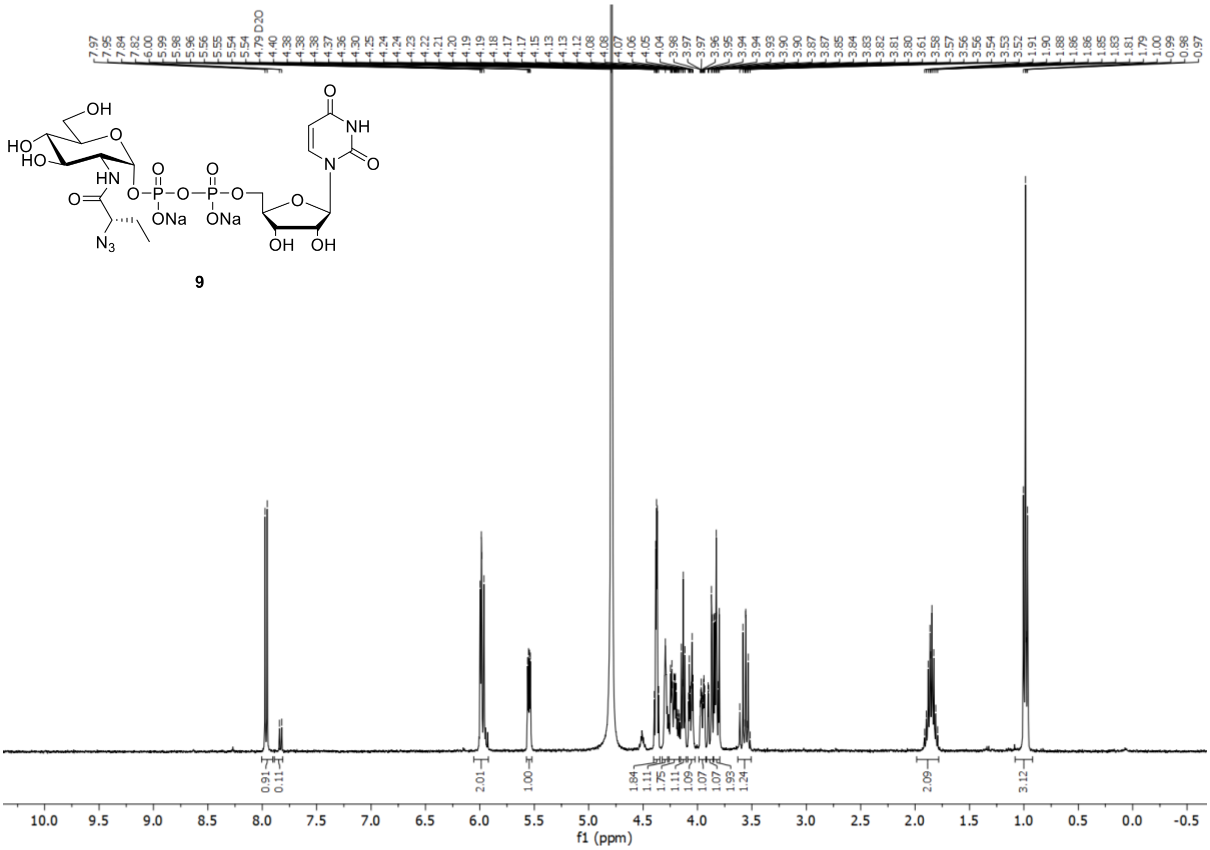

9

^{13}C NMR, D_2O, 600 MHz

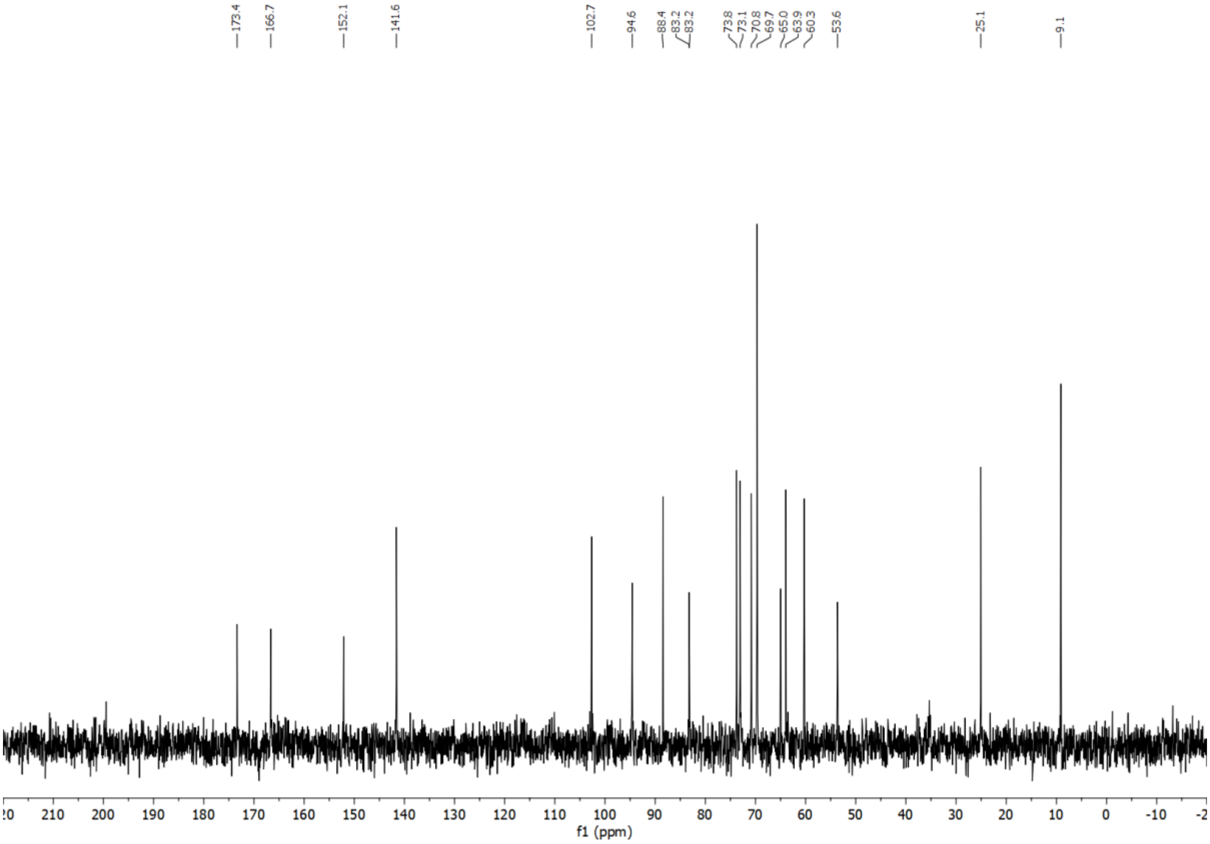

^{1}H NMR, D_2O, 400 MHz

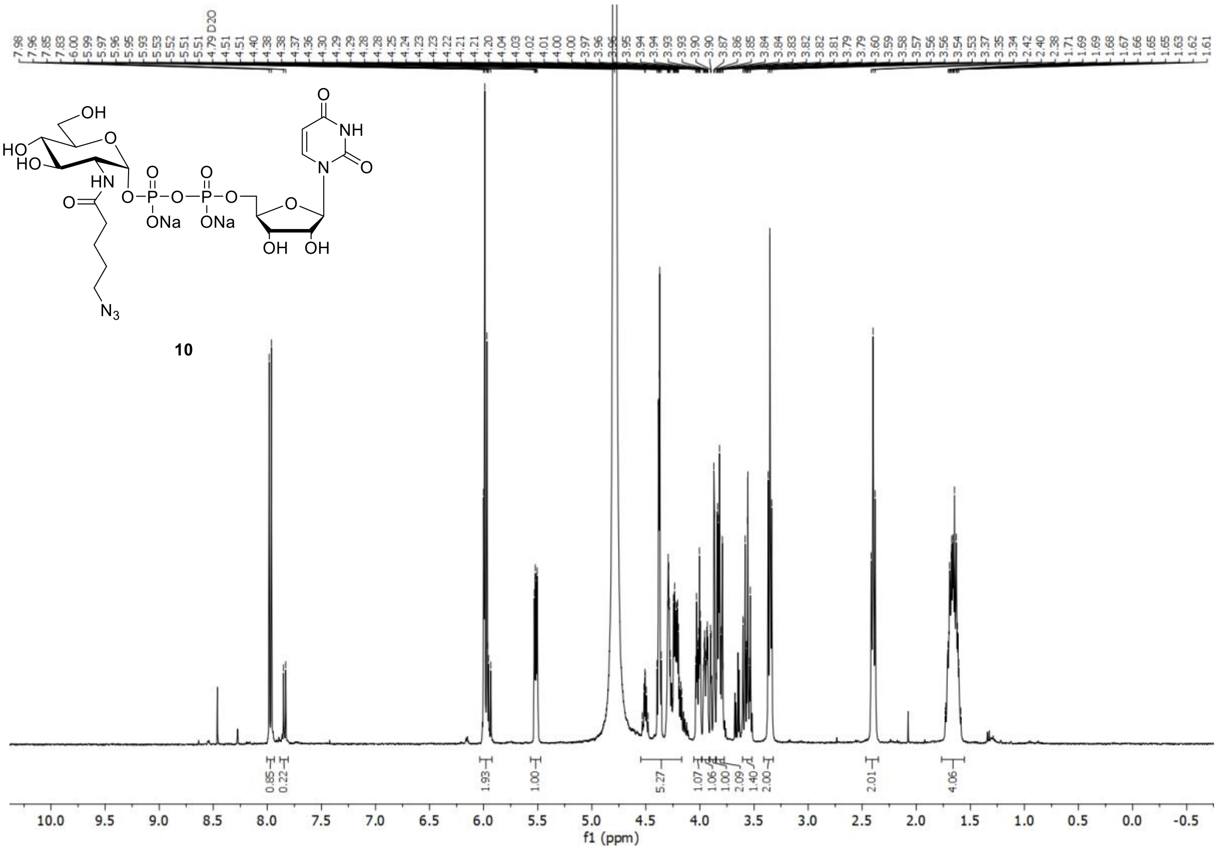

^{13}C NMR, D_2O, 400 MHz

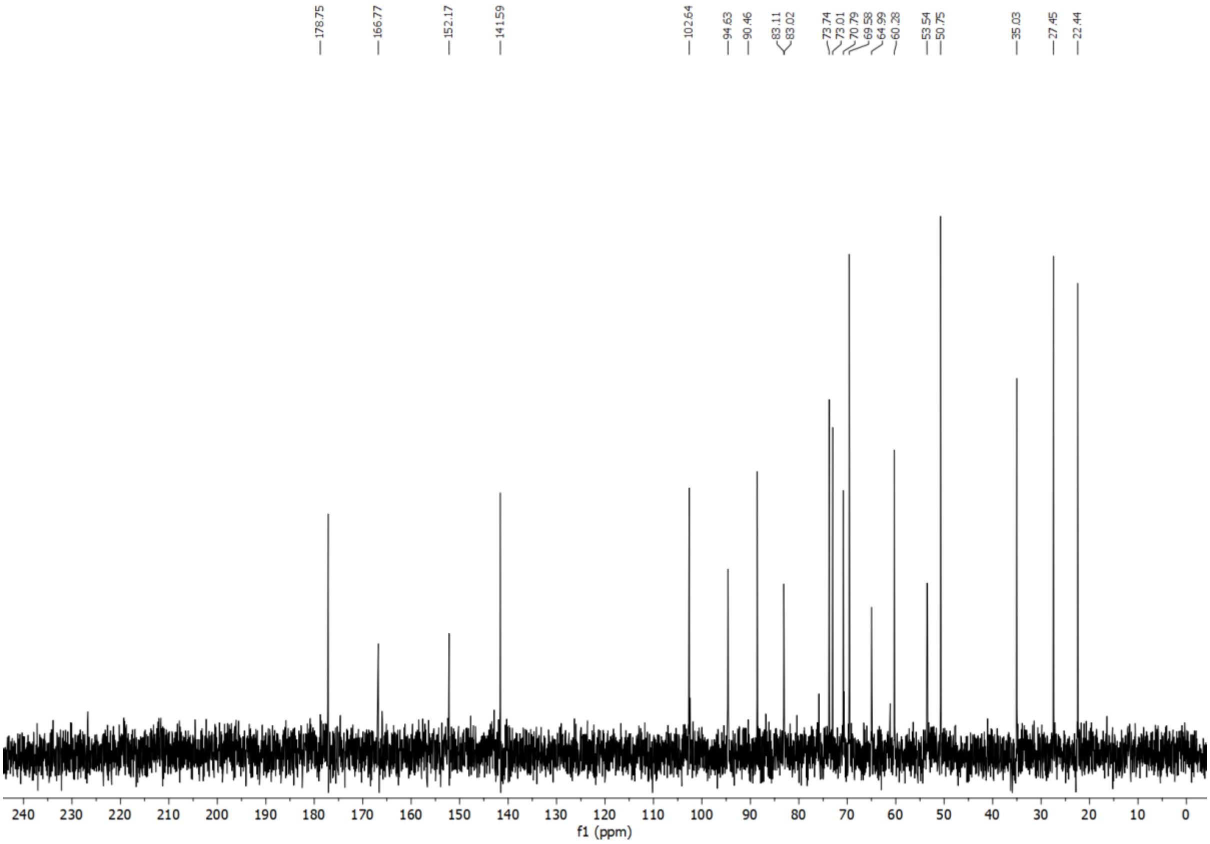

References

(1) Cioce, A.; Calle, B.; Rizou, T.; Lowery, S. C.; Bridgeman, V. L.; Mahoney, K. E.; Marchesi, A.; Bineva-Todd, G.; Flynn, H.; Li, Z.; Tastan, O. Y.; Roustan, C.; Soro-Barrio, P.; Rafiee, M. R.; Garza-Garcia, A.; Antonopoulos, A.; Wood, T. M.; Keenan, T.; Both, P.; Huang, K.; Parmeggian, F.; Snijders, A. P.; Skehel, M.; Kjær, S.; Fascione, M. A.; Bertozzi, C. R.; Haslam, S. M.; Flitsch, S. L.; Malaker, S. A.; Malanchi, I.; Schumann, B. Cell-Specific Bioorthogonal Tagging of Glycoproteins. *Nat. Commun.* **2022,** *13* (1), 1–18. DOI: 10.1038/s41467-022-33854-0.

(2) Debets, M. F.; Tastan, O. Y.; Wisnovsky, S. P.; Malaker, S. A.; Angelis, N.; Moeckl, L. K. R.; Choi, J.; Flynn, H.; Wagner, L. J. S.; Bineva-Todd, G.; Antonopoulos, A.; Cioce, A.; Browne, W. M.; Li, Z.; Briggs, D. C.; Douglas, H. L.; Hess, G. T.; Agbay, A. J.; Roustan, C.; Kjaer, S.; Haslam, S. M.; Snijders, A. P.; Bassik, M. C.; Moerner, W. E.; Li, V. S. W.; Bertozzi, C. R.; Schumann, B. Metabolic Precision Labeling Enables Selective Probing of O-Linked N-Acetylgalactosamine Glycosylation. *Proc. Natl. Acad. Sci. U. S. A.* **2020,** *117* (41), 25293–25301. DOI: 10.1073/pnas.2007297117.

(3) Moremen, K. W.; Ramiah, A.; Stuart, M.; Steel, J.; Meng, L.; Forouhar, F.; Moniz, H. A.; Gahlay, G.; Gao, Z.; Chapla, D.; Wang, S.; Yang, J. Y.; Prabhakar, P. K.; Johnson, R.; Rosa, M. Dela; Geisler, C.; Nairn, A. V.; Seetharaman, J.; Wu, S. C.; Tong, L.; Gilbert, H. J.; Labaer, J.; Jarvis, D. L. Expression System for Structural and Functional Studies of Human Glycosylation Enzymes. *Nat. Chem. Biol.* **2018,** *14* (2), 156–162. DOI: 10.1038/nchembio.2539.

(4) Darby, J. F.; Gilio, A. K.; Piniello, B.; Roth, C.; Blagova, E.; Hubbard, R. E.; Rovira, C.; Davies, G. J.; Wu, L. Substrate Engagement and Catalytic Mechanisms of N-Acetylglucosaminyltransferase v. *ACS Catal.* **2020,** *10* (15), 8590–8596. DOI: 10.1021/acscatal.0c02222.

(5) Bieniossek, C.; Imasaki, T.; Takagi, Y.; Berger, I. MultiBac: Expanding the Research Toolbox for Multiprotein Complexes. *Trends Biochem. Sci.* **2012,** *37* (2), 49–57. DOI: 10.1016/j.tibs.2011.10.005.

(6) Trowitzsch, S.; Bieniossek, C.; Nie, Y.; Garzoni, F.; Berger, I. New Baculovirus Expression Tools for Recombinant Protein Complex Production. *J. Struct. Biol* **2010,** *172* (1), 45–54. DOI: 10.1016/j.jsb.2010.02.010.

(7) Stanley, P.; Caillibot, V.; Siminovitch, L. Selection and Characterization of Eight Phenotypically Distinct Lines of Lectin-Resistant Chinese Hamster Ovary Cells. *Cell* **1975,** *6* (2), 121–128. DOI: 10.1016/0092-8674(75)90002-1.

(8) Weinstein, J.; Sundaram, S.; Wang, X.; Delgado, D.; Basil, R.; Stanley, P. A Point Mutation Causes Mistargeting of Golgi GlcNAc-TV in the Lec4A Chinese Hamster Ovary Glycosylation Mutant. *J. Biol. Chem.* **1996,** *271* (44), 27462–27469. DOI: 10.1074/jbc.271.44.27462.

(9) Makrydaki, E.; Donini, R.; Krueger, A.; Royle, K.; Moya Ramirez, I.; Kuntz, D. A.; Rose, D. R.; Haslam, S. M.; Polizzi, K. M.; Kontoravdi, C. Immobilized Enzyme Cascade for Targeted Glycosylation. *Nat. Chem. Biol.* **2024,** 1–10. DOI: 10.1038/s41589-023-01539-4.

(10) North, S. J.; Huang, H. H.; Sundaram, S.; Jang-Lee, J.; Etienne, A. T.; Trollope, A.; Chalabi, S.; Dell, A.; Stanley, P.; Haslam, S. M. Glycomics Profiling of Chinese Hamster Ovary Cell Glycosylation Mutants Reveals N-Glycans of a Novel Size and Complexity. *J. Biol. Chem.* **2010,** *285* (8), 5759–5775. DOI: 10.1074/jbc.M109.068353.

(11) Winn, M. D.; Ballard, C. C.; Cowtan, K. D.; Dodson, E. J.; Emsley, P.; Evans, P. R.; Keegan, R. M.; Krissinel, E. B.; Leslie, A. G. W.; McCoy, A.; McNicholas, S. J.; Murshudov, G. N.; Pannu, N. S.; Potterton, E. A.; Powell, H. R.; Read, R. J.; Vagin, A.; Wilson, K. S. Overview of

the CCP4 Suite and Current Developments. *Acta Crystallogr., Sect. D: Biol. Crystallogr.* **2011,** *67 (4),* pp 235–242. DOI: 10.1107/S0907444910045749.

(12) Evans, P. R.; Murshudov, G. N. How Good Are My Data and What Is the Resolution? *Acta Crystallogr., Sect. D: Biol. Crystallogr.* **2013,** *69* (7), 1204–1214. DOI: 10.1107/S0907444913000061.

(13) Winter, G.; Lobley, C. M. C.; Prince, S. M. Decision Making in Xia2. *Acta Crystallogr., Sect. D: Biol. Crystallogr.* **2013,** *69* (7), 1260–1273. DOI: 10.1107/S0907444913015308.

(14) Winter, G.; Waterman, D. G.; Parkhurst, J. M.; Brewster, A. S.; Gildea, R. J.; Gerstel, M.; Fuentes-Montero, L.; Vollmar, M.; Michels-Clark, T.; Young, I. D.; Sauter, N. K.; Evans, G. DIALS: Implementation and Evaluation of a New Integration Package. *Acta Crystallogr., Sect. D: Struct. Biol.* **2018,** *74* (2), 85–97. DOI: 10.1107/S2059798317017235.

(15) Murshudov, G. N.; Skubák, P.; Lebedev, A. A.; Pannu, N. S.; Steiner, R. A.; Nicholls, R. A.; Winn, M. D.; Long, F.; Vagin, A. A. REFMAC5 for the Refinement of Macromolecular Crystal Structures. *Acta Crystallogr., Sect. D: Biol. Crystallogr.* **2011,** *67* (4), 355–367. DOI: 10.1107/S0907444911001314.

(16) Emsley, P.; Lohkamp, B.; Scott, W. G.; Cowtan, K. Features and Development of Coot. *Acta Crystallogr., Sect. D: Biol. Crystallogr.* **2010,** *66* (4), 486–501. DOI: 10.1107/S0907444910007493.

(17) Chen, V. B.; Arendall, W. B.; Headd, J. J.; Keedy, D. A.; Immormino, R. M.; Kapral, G. J.; Murray, L. W.; Richardson, J. S.; Richardson, D. C. MolProbity: All-Atom Structure Validation for Macromolecular Crystallography. *Acta Crystallogr., Sect. D: Biol. Crystallogr.* **2010,** *66* (1), 12–21. DOI: 10.1107/S0907444909042073.

(18) Šali, A.; Blundell, T. L. Comparative Protein Modelling by Satisfaction of Spatial Restraints. *J. Mol. Biol.* **1993,** *234* (3), 779–815. DOI: 10.1006/jmbi.1993.1626.

(19) Gordon, J. C.; Myers, J. B.; Folta, T.; Shoja, V.; Heath, L. S.; Onufriev, A. H++: A Server for Estimating PKas and Adding Missing Hydrogens to Macromolecules. *Nucleic Acids Res.* **2005,** *33* (Web Server issue), W368-371. DOI: 10.1093/nar/gki464.

(20) Maier, J. A.; Martinez, C.; Kasavajhala, K.; Wickstrom, L.; Hauser, K. E.; Simmerling, C. Ff14SB: Improving the Accuracy of Protein Side Chain and Backbone Parameters from Ff99SB. *J. Chem. Theory Comput.* **2015,** *11* (8), 3696–3713. DOI: 10.1021/acs.jctc.5b00255.

(21) He, X.; Man, V. H.; Yang, W.; Lee, T. S.; Wang, J. A Fast and High-Quality Charge Model for the next Generation General AMBER Force Field. *J. Chem. Phys.* **2020,** *153* (11), 114502. DOI: 10.1063/5.0019056.

(22) Kirschner, K. N.; Yongye, A. B.; Tschampel, S. M.; González-Outeiriño, J.; Daniels, C. R.; Foley, B. L.; Woods, R. J. GLYCAM06: A Generalizable Biomolecular Force Field. Carbohydrates. *J. Comput. Chem.* **2008,** *29* (4), 622–655. DOI: 10.1002/jcc.20820.

(23) Case, D. A.; Aktulga, H. M.; Belfon, K.; Cerutti, D. S.; Cisneros, G. A.; Cruzeiro, V. W. D.; Forouzesh, N.; Giese, T. J.; Götz, A. W.; Gohlke, H.; Izadi, S.; Kasavajhala, K.; Kaymak, M. C.; King, E.; Kurtzman, T.; Lee, T. S.; Li, P.; Liu, J.; Luchko, T.; Luo, R.; Manathunga, M.; Machado, M. R.; Nguyen, H. M.; O'Hearn, K. A.; Onufriev, A. V.; Pan, F.; Pantano, S.; Qi, R.; Rahnamoun, A.; Risheh, A.; Schott-Verdugo, S.; Shajan, A.; Swails, J.; Wang, J.; Wei, H.; Wu, X.; Wu, Y.; Zhang, S.; Zhao, S.; Zhu, Q.; Cheatham, T. E.; Roe, D. R.; Roitberg, A.; Simmerling, C.; York, D. M.; Nagan, M. C.; Merz, K. M. AmberTools. *J. Chem. Inf. Model.* **2023,** *63* (20), 6183–6191. DOI: acs.jcim.3c01153.

(24) Choi, J.; Wagner, L. J. S.; Timmermans, S. B. P. E.; Malaker, S. A.; Schumann, B.; Gray, M. A.; Debets, M. F.; Takashima, M.; Gehring, J.; Bertozzi, C. R. Engineering Orthogonal

Polypeptide GalNAc-Transferase and UDP-Sugar Pairs. *J. Am. Chem. Soc.* **2019,** *141* (34), 13442–13453. DOI: 10.1021/jacs.9b04695.

(25) Hernández, H.; Robinson, C. V. Determining the stoichiometry and interactions of macromolecular assemblies from mass spectrometry. **2007,** *Nat. Protoc. 2* (3), 715-726. DOI: 10.1038/nprot.2007.73.
